# Social prescribing to improve health and well-being of patients presenting with non-medical health related social needs in primary care: Study protocol of a multi-center randomized controlled pragmatic feasibility trial

**DOI:** 10.1371/journal.pone.0322372

**Published:** 2025-05-23

**Authors:** Hendrik Napierala, Niklas Jeske, Stephanie Roll, Juliane Köberlein-Neu, Wolfram J. Herrmann

**Affiliations:** 1 Institute of General Practice and Family Medicine, Charité – Universitätsmedizin Berlin, Berlin, Germany; 2 Institute of Social Medicine, Epidemiology and Health Economics, Charité – Universitätsmedizin Berlin, Berlin, Germany; 3 Schumpeter School of Business and Economics, Bergische Universität Wuppertal, Wuppertal, Germany; PLOS: Public Library of Science, UNITED KINGDOM OF GREAT BRITAIN AND NORTHERN IRELAND

## Abstract

**Background:**

Non-medical health related social problems are highly prevalent in primary care. Even though there is a wide range of non-clinical support and services available in German communities, which can potentially meet the needs of the affected patients, there are no formalized connections between these services and primary care. One potential solution is social prescribing (SP) providing general practitioners (GPs) with a non-medical referral option, which can accompany existing treatments to improve health and well-being. The goal of this trial is to test the feasibility of a randomized controlled trial and potential benefit of SP in the primary care setting in Germany.

**Methods:**

This trial is designed as a multi-center, two-armed, open, exploratory, individually randomized (2:1) controlled, pragmatic, feasibility trial. 300 adult patients presenting with one or more non-medical health-related social needs will be recruited in GP practices in Berlin and Brandenburg, Germany. Participants in the intervention group will receive a referral to a link worker. The link worker assesses the social needs, agrees on a goal and sets up an action plan together with the patient. The link worker then connects the patient to suitable non-clinical support and services in the community (e.g., volunteering opportunities). The GP is informed about the actions taken through a feedback form. Participants in the control group will receive treatment-as-usual plus a brochure with information about local offers of non-clinical support and services in the community. Follow-up per patient is 6 months with measurements at 3 and 6 months. Primary endpoint is the feasibility of the trial measured by (1) proportion of participants who have at least one appointment with the link worker (intervention arm only), (2) proportion of participants that drop out of the trial before the 6-month follow-up (both arms). Secondary endpoints include further feasibility endpoints (acceptability, practicality, demand), clinical endpoints such the health status and wellbeing, and a goal-based outcome for the intervention group. The trial is accompanied by a process evaluation, including qualitative episodic interviews with patients and stakeholders. Furthermore, a description of selected economic consequences of SP and its intervention costs will be conducted.

**Discussion:**

This trial will assess the feasibility of implementing SP in the German primary care setting and will provide information and data necessary to plan a confirmatory trial. Implementing SP could be an adequate solution to address psychosocial problems in primary care.

**Trial registration:**

German Clinical Trials Registry, DRKS-ID: DRKS00034654. Registered on August 27th.2024. https://drks.de/search/en/trial/DRKS00034654

## Introduction

### Background and rationale

Non-medical health related social problems are highly prevalent in primary care [1]. They can significantly influence the incidence of mental and somatic conditions [2–4], but can also be caused by (chronic) diseases. They are associated with relevant economic costs, such as sick leave and long-term absence from work [5–7]. Non-medical health related social problems are operationalized through the International Classification in Primary Care 3nd Edition (ICPC-3) in chapter ZC “Social Problems” [8] and include problems such as loneliness, problems in the family and at work, as well as financial difficulties [1,9]. These problems disproportionally affect people with a lower socioeconomic status and increase health inequalities [10].

Although non-medical health related social problems are common in general practice, General Practitioners (GPs) can target only a few problems during their consultations [11]. Although there is an extensive range of non-clinical support and services available in the communities in Germany, there are no formalized connections between these services and primary care [12]. Thus, it is difficult for both GPs and patients to choose the right point of entry when problems arise. This calls for low threshold, cooperative solutions [1].

Several potential solutions have been suggested, including integrated primary care centres, provision of social work in primary care, and social prescribing (SP) [13]. SP equips GPs with a non-medical referral option, which can accompany existing treatments to improve health and well-being [14]. SP was developed in the United Kingdom and has become widely established in the National Health Service in recent years. Globally, SP schemes have been implemented in several countries such as Canada and Singapore, but also Austria and the Netherlands [15]. In a recent survey we could show that there is a high interest in Social Prescribing among German GPs [13,16].

From the existing evidence, the need for randomized controlled trials to assess the efficacy of SP - especially outside of the United Kingdom, is evident [17]. However, as there is limited experience with establishing SP in Germany, a feasibility study is a necessary first step to clarify aspects for a future randomized trial. Important aspects are the acceptability of the trial by potential participants, loss to follow-up, the number of appointments between patients and link workers needed, the feasibility of endpoint measurements as well as the acceptance of the link worker role in the German health care system. This evidence is essential before implementing Social Prescribing into routine care in Germany [18].

### Objectives

The goal of this trial is to test the feasibility of a randomized controlled trial and potential benefit of SP in the primary care setting in Germany.

### Trial design

**Fig 1:**
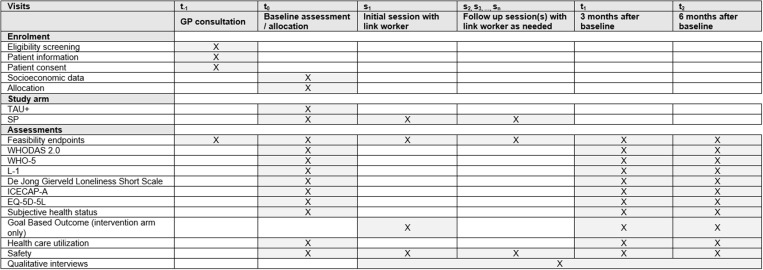
SPIRIT schedule of enrolment, interventions and assessments for the trial.

**Fig 2:**
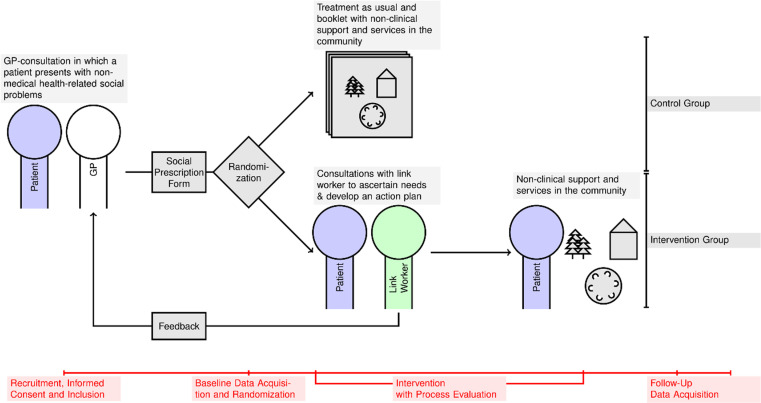
Design of the feasibility trial.

The trial is designed as a multi-center, two-armed open, exploratory, individually randomized controlled, pragmatic, feasibility trial. The study procedures follow the guideline for good clinical practice E6 (R2). The study protocol used the SPIRIT reporting guidelines [19]. A SPRIT schedule can be found in Fig 1. An overview of the study design can be found in Fig 2.

SP is compared with treatment-as-usual plus a brochure with information about local offers for non-clinical support and services in the community (TAU+). We decided on a multi-center approach to reach the necessary sample size and allow the results to be more generalizable. This also allows for more flexibility in the delivery of the intervention, which will be analyzed in the process evaluation. In this feasibility trial, study centers are GP practices in Berlin and Brandenburg.

**Fig 3:**
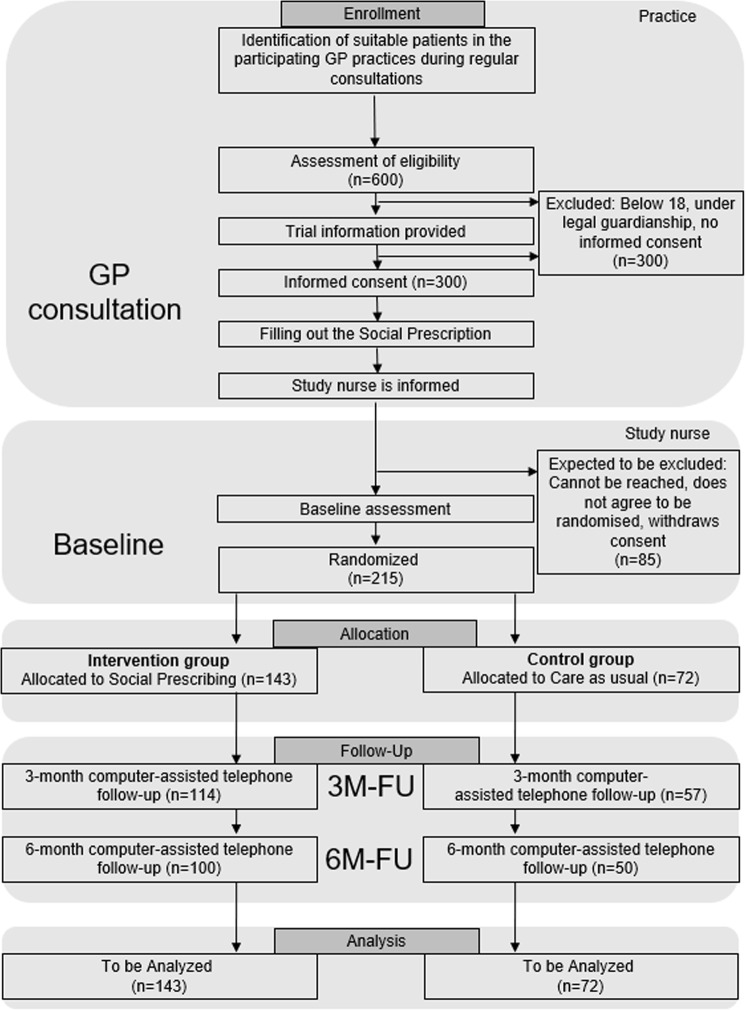
Expected participant flow.

We decided to perform individual randomization in a 2:1 allocation ratio (intervention:control). A randomized design was chosen to assess the feasibility of individual randomization in the primary care setting. We decided for a 2:1 allocation ratio to ensure that enough participants are in the intervention arm for the main feasibility endpoints. A control group is needed to provide information on the feasibility of the proposed control group design and trial processes especially regarding loss to follow-up in the control group. Cross-contamination will be analyzed in the qualitative process evaluation to assess the feasibility of individual randomization compared to cluster randomization. A highly pragmatic approach was chosen because the intention of the future confirmatory trial will be to inform real world clinical choices of implementing SP into routine primary care [20,21]. Fig 3 shows the expected patient flow following the CONSORT flow diagram.

## Methods: Participants, interventions and outcomes

### Study setting

The trial is conducted in the primary care setting. Study centers are nine GP practices in Berlin and Brandenburg. After recruitment in the GP practice and issuing of a social prescription, the Link worker sees the patients preferably in the GP practice, alternatively as a home visit or by telephone. The study centers are the following:

Dr. med. Kathrin Hecker

Landsberger Allee 44

10249 Berlin

Study site initiated 25.10.2024

Ärzte am Hermannplatz

Gerd Michels

Kottbusser Damm 72

10967 Berlin

Study site initiated 12.09.2024

MVZ Praxis Korok

Rheinsteinstr. 1

10318 Berlin

Study site initiated 18.09.2024

Hausarztpraxis Lichtenberg

Iris Boehmer

Möllendorffstr. 45

10367 Berlin

Study site initiated 19.09.2024

Gesundheitszentrum Haus Nazareth

Dr. med. Burghard Storm

Wrangelstr. 6/7

12165 Berlin

Study site initiated 04.11.2024

Hausarztpraxis Dashti

Dr. med. Hiwa Dashti

Am Paschenberg 30

16225 Eberswalde

Study site initiated 09.09.2024

Hausarzt Dr. Zerbaum & Kollegen MVZ

Dr. med. Mario Zerbaum

Petersilienstraße 12

14776 Brandenburg

Study site initiated 16.09.2024

Hausarztpraxis Neukölln

Jihan Saeed

Hermannstraße 52

12049 Berlin

Study site initiated 24.02.2025

Praxis Weichselstraße

Dr. med. Aliona Cuadrado

Weichselstr. 59

12045 Berlin

Study site initiated 18.02.2025

### Eligibility criteria

Following the concept of pragmatic trials, we will keep the inclusion and exclusion criteria to a minimum to improve generalizability and enable the implementation of the intervention into routine care. Eligibility criteria for the GP practices were chosen to allow generalizability to the German primary care system.

#### Eligibility criteria for principal investigators in the study sites (GP practices).

Inclusion criteria:

- Family doctor (specialized in general practice or internal medicine; Hausärzt:in und Fachärzt:in für Allgemeinmedizin or Innere Medizin): Study validity and generalizability for the German primary care system
- Training in “basic psychosomatic care”: Basic qualification in mental health – patient safety

Exclusion criteria

- Practices with more than 50% care volume in specialized care (e.g., SAPV, infectiology, substance abuse therapy, psychotherapy): Generalizability for the German primary care system

#### Eligibility criteria for patients.

Inclusion criteria:

- Adult (18 years or older): The broad inclusion criterion is intentional and shall equip GPs with the potential to refer patients with a wide variety of needs to the link worker
- One or more non-medical health related social needs (ICPC-3, chapter ZC)

Exclusion criteria:

- Under legal guardianship for health care matters
- Member of the same household has been recruited for the study

A non-medical health related social need comprises a problem from the following list (International classification of primary care 3^rd^ edition, chapter ZC Social Problems influencing health status [8]):

- Problems associated with finances (incl. poverty)
- Social welfare problem (incl. lack of social benefits, problems with inability to work)
- Work problems
- Unemployment problem
- Loneliness, social isolation
- Partner, child, parent, family member or other person relationship problem (incl. mobbing)
- Loss or death of partner, child, parent or family member problem
- Illness of partner, child parent or family member problem
- Education problem (incl. illiteracy, lack of school leaving certificate)
- Problem associated with discrimination
- Problem due to residence status/residence permit
- Violence problem
- Housing problem (incl. homelessness or imminent homelessness)
- Legal problem (imminent prison sentence or similar)
- Health care system related problem (i.e., nursing care)
- Other specified social problem influencing health status

### Who will take informed consent?

Screening of potential study participants will be carried out in the study centers (GP practices) by the general practitioners based on the inclusion and exclusion criteria. Written informed consent will be obtained at the study centers by the GPs.

### Additional consent provisions for collection and use of participant data and biological specimens

The patients can consent to being contacted by the study team for participation in semi-structured interviews as part of the qualitative process evaluation. Participating GPs, practice staff, link workers, study team and representatives of local community services will also be asked to participate in qualitative interviews.

### Ethics approval and consent to participate

Ethics approval has been granted by the Institutional Review Board at Charité – Universitätsmedizin Berlin, reference number: EA1/104/24 (July 11^th^ 2024) and the ethics commission of the state medical association Brandenburg, reference number: 2024–94-BO (August 6^th^ 2024). The study will be carried out in accordance with the Helsinki Declaration and German Law. Written, informed consent to participate will be obtained from all participants.

## Interventions

### Explanation for the choice of comparators

To be as pragmatic as possible we chose treatment-as-usual as comparator. However, to motivate persons in the control group to adhere to study procedures, we added a physical brochure to be sent out to the participants in the control group. These brochures contain information on local support to the TAU+ participants. In some of the communities, such brochures already exist. In communities without such an offer, we compiled a brochure together with local stakeholders.

### Intervention description

Through Social Prescribing a GP can refer patients with non-medical health-related social needs to a so called “link worker”. A specifically trained professional with a background in a health or social profession. In the first step, the link worker assesses the needs of the patients and agrees with the patient on a goal. The link worker develops an action plan together with the patient. This action plans includes referrals to existing non-clinical support and services in the community. The link worker supports the patient to access the service agreed on. At the end of the intervention, the link worker provides written feedback to the prescribing GP [22]. The contact between link worker and patient usually involves several consultations over the course of several weeks (Table 1). The conceptual model for the intervention was adapted from Muhl et al. [14].

**Table 1:** Description of the intervention using the TIDieR framework [23].

Item		Justification
**Brief name**	Social Prescribing	
**Why**	Social Prescribing allows patients with non-medical health-related social needs to be referred to non-clinical support and services in the community.	This draws on already existing services.
**What**	For each community/neighborhood, the link worker will set up a database which contains information and contact details on service provision needed for social prescribing. This database builds on information provided by the brochures used in the control arm. The database will be iteratively extended during the intervention.	The database will help to build up a sustainable intervention.
	1. The GP fills out a custom social prescription depicting needs and highlighting the main need of the patient. 2. The GP or medical practice assistant (in German: Medizinische Fachangestellte or “MFA”) enters the social prescription into a secure online form provided by RedCap. 3. The study nurse conducts baseline assessment and allocation to the intervention arm. Then the study nurse sets up the first appointment between patient and link worker. 4. The link worker has several appointments with the patient. They first assess the patient’s needs, set a main goal together and develop and action plan to which local service providers to connect the patient. 4. The link worker supports the patient accessing the agreed on local service providers. 5. The link worker informs the GP about where the patient was referred to through a standardized form (Feedback).	This setup builds on the definition of SP provided by Muhl et al. [14] and existing SP programs in the UK. The intervention is tailored to the German primary care context by involving “MFA”.
**Who provides**	The link worker will be a specifically trained professional holding at least a bachelor’s degree majoring either in a health, health care, social or social care field, or a vocational training in a health, health care, social or social care profession or a training in psychological counselling.	Link Workers need a basic understanding of health and social care.
**How**	Individual service provision, preferably face to face or alternatively by phone.	As needs are highly individual, there must be tailored service provision.
**Where**	Preferably in the GP practice, alternatively as a home visit or by telephone.	This allows for more flexibility fitting the needs of the patients.
**When and How Much**	The first consultation should take place two weeks after study inclusion at the latest. The first consultation will last for up to 45 minutes. The link worker and patient schedule as many appointments as necessary, usually in a time frame of four to twelve weeks after study inclusion.	For the vulnerable patient group, it is most important to follow up, if they reach the fitting local services. Thus, a follow-up must be an integral part.

Link workers will be trained before the intervention starts according to the SP link worker workforce development framework [24]. Link workers will have time to get to know the communities and present themselves not only to the GP practices, but also to the institutions providing local services. Link workers will build up an electronic database with local services and organizations where patients can potentially be referred to. Additionally, link workers will receive a communication training by an experienced staff member of the Institute of General Practice and Family Medicine and will have the opportunity of continuous supervision. There will also be case reviews each week.

Each study center will receive a training workshop to prepare for inclusion of participants and study procedures. Study nurses will receive training regarding baseline and follow-up data assessment to ensure a standardized assessment.

### Criteria for discontinuing or modifying allocated interventions

Participants may withdraw from the programme at any time if they wish (even without giving reasons). GPs can withdraw patients at any time if they suspect that harm is inflicted though the intervention. Based on the monitoring reports made available to the Data Safety Monitoring Board (DSMB), it may recommend early termination of recruitment for the overall study or selected intervention groups.

### Strategies to improve adherence to interventions

The alignment of the intervention delivery with the study protocol is verified in weekly case reviews with the link workers.

### Relevant concomitant care permitted or prohibited during the trial

In both arms of the trial, medical care as usual by the GP and other providers will occur without any restrictions. At follow-up, we will ascertain health and social care provision to determine an influence of the intervention on care as usual in the control group and evaluate if individual randomization is feasible for a confirmatory trial.

### Provisions for post-trial care

To our best knowledge, there are no reports of damages resulting from SP interventions. Therefore, we did not anticipate provisions for post-trial care.

### Outcomes

The **primary outcome** is the feasibility of the trial measured by

(1) The proportion of participants who have at least one appointment with the link worker (intervention arm only) and
(2) The proportion of participants that drop out of the trial before the 6-month follow-up (attrition rate, both arms).

**Secondary feasibility outcomes** measured through questionnaires at 6-month follow-up and semi-structured interviews, as well as the trial documentation:

*Acceptability:*

- Satisfaction of GPs (questionnaire after last patient out) and of patients (6-month follow-up, intervention arm only) with the intervention;
- Acceptance of the intervention by patients (intervention arm only), GPs, link workers and local service providers as stated in qualitative interviews (subgroup).

*Practicality:*

- Proportion of eligible patients of all patients seen in the practice;
- Proportion of eligible patients excluded because of a language barrier;
- Proportion of eligible patients consenting to the randomized controlled trial;
- Proportion of consenting patients randomized and starting the intervention;
- Proportion of patients adhering to the appointments with link worker of these (intervention-arm only);
- Estimated time spent by GPs;

*Demand/Utilization:*

- Non-medical health related social problems assessed by GP:
- Non-medical health related social problems assessed by link worker;
- Frequency of link worker consultations per patient;
- Length of link worker visits per patient;
- Delivery mode of the link worker interaction (in person (home visit, in GP practice), phone);

Services recommended by the link worker

- Utilization of recommended non-clinical support and services by the patient.

**Secondary clinical outcome measures** (patient reported outcomes, PROMs) are assessed by computer assisted telephone interview at 3-months follow-up and 6-months follow-up.

Following the definition of SP by Muhl et al. [15], clinical endpoints are health (measured by WHODAS 2.0), well-being (measured by WHO-5, ICECAP-A and L-1) and community connection (assessed qualitatively). Patient questionnaires are kept to a minimum to avoid attrition. They consist of the following outcome measures (further information on content, validity, and reliability can be found in chapter *Plans for assessment and collection of outcomes*):

- Health status (WHODAS 2.0) [25,26];
- Subjective health status [27];
- Mental wellbeing (WHO-5) [28,29];
- Capability Wellbeing (ICECAP-A) [30];
- Health-related quality of life (EQ-5D-5L) [31];
- General life satisfaction (Short scale L-1 for recording general life satisfaction) [32,33];
- Loneliness (De Jong Gierveld Loneliness Short Scale) [34];
- Goal based outcome (GBO) [35–37];
- Resource use (relevant parts of PECUNIA RUM and EHIS-GeDA, decision on items was made referring to current systematic reviews [38–41]);
- Safety (Mortality, emergency department visits, unplanned hospital admissions, suicidality, suicide attempts, other adverse events).

Topics of the qualitative interviews are: Acceptance, practicability, need/use, implementation of the study, potentials and hurdles. For the patients, the integration into the neighbourhood will also be discussed.

### Participant timeline

Patients will be recruited to the study during a GP consultation (t-1, cf. Fig 1, Fig 2). After baseline assessment and study arm allocation (t0), patients in the intervention arm will receive sessions with a link worker (s1-sn). 3 and 6 months after baseline we will conduct follow-up data assessments (t1, t2).

### Sample size

Because this is an exploratory feasibility study without confirmatory hypothesis testing, we performed no formal sample size calculation. From prior experience, we assume that analyzing 215 participants in total (2:1 ratio: 143 in the SP arm and 72 in the TAU + arm) is sufficient to descriptively determine the feasibility and acceptance aspects of the trial and to obtain further information for the planning of the subsequent confirmatory trial.

Feasibility outcomes will be assessed depending on the type of outcome (e.g., n = 215 for attrition rates). In this patient group, a drop out of about 30% is expected, so that follow-up data for secondary clinical endpoints of 150 (n = 100 in the SP and n = 50 in the TAU + arm) patients can be assessed at 6-months follow-up (Fig 3).

For the qualitative study part, a total of n = 30–40 qualitative, semi-structured episodic interviews will be conducted with patients from the intervention and control groups (n = 15–20), as well as participating GPs, practice staff, link workers, study staff and representatives of local services (n = 15–20).

### Recruitment

Based on the commitments of the participating GP practices, we expect it to be feasible to recruit more than 570 patients during a 6-month recruitment period. Thus, even considering lower recruitment due to external factors such as influenza epidemics, it is very well possible to recruit the 300 estimated patients in this feasibility trial.

Participating GP practices receive a compensation of 50 € per recruited patient.

## Assignment of interventions: allocation

### Sequence generation

All patients who give consent for participation and who fulfil the inclusion criteria will be randomized.

The randomization code will be generated by an independent person (not otherwise involved in the study) as a block randomization (with variable block length) stratified by center (GP practice) with 2:1 allocation ratio. Thus, allocation will be conducted without any influence of the principal investigators, doctors, or link workers to obtain comparable treatment groups.

### Concealment mechanism

Randomization codes will be obtained from the RedCap (Research Electronic Data Capture, version 14.0 or higher) database after entry of participant data, with the allocation being concealed.

### Implementation

Participants will be enrolled by the GPs in the participating practices and will be allocated to the study groups by the study nurses.

## Assignment of interventions: Blinding

### Who will be blinded

This is an open study. Due to the nature of the study intervention, blinding is not possible for treating physicians, link workers, or patients. Due to the 2:1 allocation blinding of the biometrician is also not feasible.

### Procedure for unblinding if needed

There is no blinding.

## Data collection and management

### Plans for assessment and collection of outcomes

Data assessment is performed by study nurses independent from the intervention delivery and not located at the practices to minimize measurement bias. Computer-assisted telephone interviews by trained study nurses allow for decentralized data collection that caters to the needs of the vulnerable patient group which might not be capable to fill out questionnaires by themselves.

Feasibility outcomes will be collected through process parameters in the eCRF and trial documentation as well as qualitative interviews. Satisfaction is assessed through two questions for each group (General satisfaction with the service, recommendation of the service to friends and family).

The PROMS consist of the following questionnaires

- *Health status (WHODAS 2.0)*

The WHO Disability Assessment Schedule 2.0 is a generic patient reported outcome measure for health status. The domains of WHODAS 2.0 are based on the conceptual framework of the WHO’s International Classification of Functioning, Disability and Health (ICF), which has been the international standard for describing and measuring health and disability since 2001. All six ICF domains (Cognition, Mobility, Self-care, Social interaction, Life activities and Social participation) are covered by the WHODAS 2.0 tool. It shows high internal consistency, a high test-retest reliability as well as high validity in comparison with other instruments [25,26]. A computer adaptive form with 12 item version is available in German.

- *Mental wellbeing (WHO-5)*

The World Health Organization-Five Well-Being Index (WHO-5) is a short self-reported measure of current mental wellbeing. The scale has an adequate validity as an outcome measure in clinical trials [28] and normative data is available for a German version [29].

- *General life satisfaction (Short scale L-1 for recording general life satisfaction):*

The short scale L-1 for recording general life satisfaction consists of the item formulation established in SOEP [32,33]. The scale contains only one item with the following wording: “How satisfied are you at present, all in all, with your life”. The tool has been validated with a good reliability in German and normative data is available [33].

- *Loneliness (De Jong Gierveld Loneliness Short Scale):*

The short version of the De Jong Gierveld Loneliness Scale comprises 6 items and includes two subscales on emotional and social loneliness [34]. The questionnaire is valid and reliable. A validated German translation is available.

- *Capability Wellbeing (ICECAP-A):*

The ICEpop CAPability measure for Adults is a measure of well-being for the general adult population (aged 18 and over) for use in health economic evaluation. A validated German translation exists [30].

- *Health-related quality of life (EQ-5D-5L):*

The European Quality of Life 5 Dimensions 5 Level Version is a general instrument that can be used to assess the quality of life of patients regardless of their illness. It has been extensively validated, including in the German healthcare context [31].

- *Subjective health status*

Based on the European Health Interview and Examination Survey (EHIS) [27], the state of health is asked as follows: How is your health in general?

- *Goal based outcome (GBO):*

Link worker and participant agree on a main goal to be achieved through SP. The progress towards achieving this goal is measured using GBOs (“On a scale of zero to ten, please tell me the number that best describes how close you are to your goal today. Remember: zero means that you are as far away from your goal as you have ever been, and ten means that you have reached your goal completely.”) GBOs are proposed by two articles that assess appropriate outcomes for SP [42,43]. It is comparable to the Measure Yourself Concerns and Wellbeing (MYCaW®) outcome measure, which was validated in cancer care [35]. This outcome is highly relevant to patients because it is chosen by themselves and thus reflects the patient’s individual goals and values [36]. GBOs, such as MYCaW®, were assessed in several project reports identified in the systematic review by Napierala et al. [17], but no project provided enough data for further analysis. We found only one GBO in a feasibility study for children with autism spectrum disorder where it showed high levels of completion [37]. GBO will be measured in the intervention arm only.

- *Resource use (relevant parts of PECUNIA RUM and EHIS-GeDA):*

The PECUNIA Resource Use Measurement instrument (PECUNIA RUM) is a globally standardized, harmonized, and validated self-report tool for measuring resource use across various sectors, designed to facilitate costing from a societal viewpoint in the adult population [44]. This tool covers several relevant areas, including health and social care, education, justice, productivity losses, and informal care. In this study, we specifically employ selected items from this instrument that are pertinent to our examination of health and social care and productivity losses [38–41]. Where possible, the culturally adapted version from EHIS-GeDA is used for Germany.

The interviews of the qualitative study part will take place outside of everyday care at a later date on the basis of a separate invitation and consent. The interviews are conducted by trained members of the study team.

### Plans to promote participant retention and complete follow-up

We expect to screen about 600 adult patients with at least one psychosocial need. Of these, about 300 patients (50%) will fulfil the eligibility criteria and consent to the trial. We assume that about 215 (around 70%) of these patients will be randomized (SP: 143 and TAU + : 72), as some participants cannot be reached, or withdraw their consent.

The population in question is a highly vulnerable group and the literature shows high rates of attrition [18]. Thus, the assumed rate of loss to follow-up is about 30% between baseline and 6-month follow-up (primary feasibility outcome), with around 2/3 of the loss-to-follow up attributed to the 3-month follow-up (Fig 3). For this study, loss to follow-up (and reasons for loss to follow up) are an essential part to assess the feasibility and will be assessed in detail if possible.

### Data management

We will comply with relevant data protection laws (GDPR, local data protection laws). Data management is carried out in accordance with the specifications of the local quality management manual. The data is only stored and processed for the respective purpose.

In the informed consent form to participate in the study, the study participant authorizes the recording, processing and storage of his/her personal and medical data.

The collected data will be recorded and stored in the REDCap online database (REDCap = Research Electronic Data Capture, version 14.0 or higher) on a server of the Charité – Universitätsmedizin Berlin IT infrastructure in Germany.

The databases are only accessible to data management and authorised study personnel with individualised login. Personal identifying data of participants will be accessible only via 2-factor authentication.

The study data and study documents are archived for 10 years after the end of the study and all personally identifiable documents and data are then deleted. All research data will be anonymised 10 years after the end of the study.

Qualitative interviews are digitally recorded and transcribed using external transcription service providers. Tape recordings, transcribed protocols, questionnaires and data entry forms are stored on a protected area of the Charité server. Only authorized members of the study team have access.

### Confidentiality

Personal identifying data (PiD) is entered into REDCap by the practice staff. The research data (RD) is collected and stored separately from the PiD in another independent REDCap project; no PiD is transmitted to the survey project.

The data is recorded in REDCap using pseudonyms, which consist of consecutive numbers without initials or dates of birth of the participants. The PiD database is the re-identification list for the pseudonymised FD entered into REDCap by the study personnel.

Pseudonymized health data will be entered online by the study staff into a REDCap database via an individualized link and a secure HTTPS Internet connection using encrypted transmission (SSL encryption). The data will be transmitted to the servers in encrypted form (via an HTTPS connection) immediately after completing the questionnaire and will not be stored on the computer or tablet.

Prior to enrolment in the study, study participants must sign a written informed consent for to the data collection, the transfer and the inspection by domestic supervisory authorities and authorized representatives who are bound to confidentiality (including study monitors and auditors).

Audio files and transcripts of the qualitative interviews are stored pseudonymously in a database on the protected server area of Charité - Universitätsmedizin Berlin, which is password-protected and only accessible to persons authorized by the principal investigator. Pseudonyms consist of consecutive numbers without initials or date of birth.

### Plans for collection, laboratory evaluation and storage of biological specimens for genetic or molecular analysis in this trial/future use {33}

No biological specimens are collected as part of this study.

## Statistical methods

### Statistical methods for primary and secondary outcomes

In this feasibility trial, all results will be interpreted exploratively. Details of the statistical analyses (including definition of analysis populations, subgroups, sensitivity analyses, etc.) will be pre-specified in a statistical analysis plan (SAP). Analyses will be performed using the Full Analysis Set (FAS), which is based on the intention-to-treat principle with all available data (i.e., missing data are not imputed).

The per-protocol population is defined as patients who adhere to the protocol (details are defined in the SAP). No interim analysis is planned

#### Primary outcome.

The proportion of participants in the intervention group who attended at least one appointment with the link worker and the proportion of all participants who drop out of the study before the 6-month follow-up (dropout rate) are analysed descriptively, with frequencies and percentages, incl. 95% confidence interval per treatment group. The comparison of the dropout rate between the treatment groups is analysed descriptively and using logistic regression to determine the possible influencing factors.

#### Secondary outcomes and analyses.

Secondary endpoints are analysed descriptively (frequencies/percentages, mean/standard deviation or median/interquartile range depending on scale and distribution, each with corresponding 95% confidence interval). The association between goal-based endpoints and questionnaire type endpoints will be assessed.

Comparisons between treatment groups are performed using logistic regression (binary endpoints) or analysis of covariance (continuous endpoints, adjusted for the respective baseline value, if available), taking the centre into account.

#### Safety.

Adverse events are analysed descriptively (frequencies and percentages per treatment group).

#### Qualitative interviews.

The interviews from qualitative study part will be audio-transcribed. The transcripts will be coded thematically according to Flick [45] or using framework analysis [46,47].

#### Health economic evaluation.

The feasibility study is accompanied by a health economic evaluation that utilises the quantitative data collected in the study. The aim of this is, on the one hand, to examine the acceptance of the survey instruments on well-being (ICECAP-A), quality of life (EQ-5D-5L) and resource utilisation (PECUNIA RUM/EHIS-GeDA). In addition, the documentation of the link workers is used to extract which services were provided to patients and to determine parameters concerning the patient-link worker interaction (e.g., number and duration of meetings). The recorded service utilisation is also evaluated in a group comparison in order to identify service areas with a significant group difference. The exploratory analyses use the 5% and 10% significance levels. The findings will be used to revise the survey instruments for a follow-up study, i.e., to remove aspects of the PECUNIA RUM instrument that are less significant in the ‘social prescription’ application context and to include the range of services provided outside the healthcare system for standardised documentation and determination of standard costs.

In addition to these research process-related results, the data collected on service utilisation, quality of life and well-being are evaluated in terms of content. For the monetary evaluation of resource utilisation, the standard costs for all (privately purchased or prescribed) services and goods are calculated in euros. The cost-effectiveness of the intervention is determined using the net benefit approach. The statistical analysis of the data will be carried out using parametric or non-parametric methods, depending on the distribution of the data. Finally, relevant subgroups will be identified from a health economic perspective and statements will be made on the power of the results achieved in order to support the case number estimates for a potential follow-up study.

### Interim analyses

No interim analysis is planned

### Methods for additional analyses (e.g., subgroup analyses)

Sensitivity analyses include per-protocol-analysis based on patients complying with the protocol (as defined in the SAP) and subgroup analyses (i.e., gender; underlying psychosocial problem(s); age groups).

### Methods in analysis to handle protocol non-adherence and any statistical methods to handle missing data

The analyses using the Full Analysis Set (FAS) are performed according to the intention-to-treat principle with all available data (missing data are not imputed).

The per-protocol population is defined as patients who adhere to the protocol (details are defined in the SAP).

### Plans to give access to the full protocol, participant level-data and statistical code

An anonymized dataset will be made available in an open data repository (planned repository: Zenodo). The full study protocol can be found in the DRKS registry in German (DRKS00034654).

## Oversight and monitoring

### Composition of the coordinating centre and trial steering committee

#### Coordinating centre.

The coordinating centre is the Charité – Universitätsmedizin Berlin. This is an investigator-initiated trial (IIT) which means the Charité – Universitätsmedizin Berlin is responsible for funding, study design, management, analyses, interpretation and publishing of data.

The project will be implemented and lead by the Institute of General Practice and Family Medicine (PI and Sponsor representative: WJH; Co-PI: HN, Coordinator: NJ). The data management and statistical analysis is led by the Institute of Social Medicine, Epidemiology and Health Economic (Principal Biostatistician: SR). Charité – Universitätsmedizin Berlin is responsible for data protection aspects, such as the creation and implementation of a data protection concept.

#### Health economics evaluation.

The Center for Health Economics and Health Services Research, Schumpeter School of Business and Economics, University of Wuppertal, Wuppertal, Germany (JKN) will provide their health economics expertise during the development and analysis of the trial.

#### Advisory board.

Independent experts from British and German universities as well as an Austrian competence center will form an independent advisory board for the trial. They will monitor and supervise the trial progress, as well as provide guidance during the development of the link worker training. The independent experts will also provide the sponsor (DFG, German Research Foundation) with information and advice.

#### Steering committee.

The steering committee consists of members of Institutes of General Practice at three German universities (Berlin, Hamburg, Freiburg) that plan to implement a confirmatory trial if the feasibility trial is successful.

### Composition of the data monitoring committee, its role and reporting structure

The study will be monitored by two members of staff of the sponsor institution (Institute of General Practice and Family Medicine, Charité Universitätsmedizin Berlin): The continuous monitoring of the data received via REDCap will be performed by the study coordinator, the on-site monitoring visits will be performed by an additional member of staff, who is not directly involved in the study. The monitors will regularly send monitoring reports to the Data Safety Monitoring Board, in which they inform about the current recruitment status, deviations identified during the data and on-site monitoring, adverse events, and other issues in the conduct of the study. The first monitoring visit is supported by an independent co-monitor of the Clinical Trials Office of the Charité.

The data safety monitoring board (DSMB) consists of three experienced researchers, including a senior biometrician.

The aims of the DSMB are

- to safeguard the interests of the study participants,
- to assess the safety and efficacy of the intervention during the study and
- monitoring the implementation of the study.

The DSMB receives and reviews information on the progress and data generated by the trial.

This includes:

1. assessing data quality, including completeness,
2. monitoring recruitment numbers and loss to follow up,
3. monitoring compliance with the protocol by participants and investigators,
4. the monitoring of adverse events,
5. monitoring compliance with previous DSMB recommendations,
6. considering the ethical implications of all DSMB recommendations.

The DSMB should decide whether to recommend continuing the recruitment of participants for the trial or to stop recruitment either for all or some treatment groups and/or for all treatment groups. participants should be stopped. The recommendations of the DSMB are advisory and not executive.

The composition of the DSMB, its working methods (including decision-making) and reporting are defined in a charter.

### Adverse event reporting and harms

No adverse events associated with the intervention are described in the literature on social prescribing. However, a comprehensive monitoring procedure is established in accordance with ICH-GCP to demonstrate safety and safety, which consists of short-term notifications of individual case reports, periodic reports, safety monitoring by an independent data safety monitoring board (DSMB) and a comprehensive safety analysis of the study.

#### Definitions.

As this is an “other” study according to §15 of the Berlin Professional Code for Physicians with a complex intervention without a drug or medical device, the adverse events (AE) are not classified according to the Common Terminology Criteria for Adverse Events (CTCAE) v5.0. Instead, the following adverse events are predefined for the study:

1) Death (any cause)
2) Emergency room visit (any cause)

Visit to an emergency room. The report should be made regardless of whether the person is subsequently hospitalised or not.

3) Unplanned hospitalisation (any cause)

An unplanned hospitalisation is characterised by a hospital admission without prior appointment.

4) Suicide attempt/suicidality

A suicide attempt is defined as self-perpetrated, harmful behaviour that is intended to lead to death but does not result in death (ICD-11: MB23.R, ICPC-3: PD14).

Suicidality in this context is understood as thoughts, ideas or ruminations about the possibility of ending one’s life, ranging from thinking that one would be better off dead to formulating elaborate plans (ICD-11: MB26.A, ICPC-3: PS05).

5) Other relevant adverse events

To allow for the identification of further adverse events as assessed by the investigators.

#### Reporting.

AE are reported by the investigators (GPs) to the principal investigator within 7 days of becoming known. Training will be provided in advance. The principal investigator will perform a second evaluation. After recruitment of 50 patients or every three months at the latest, a safety report with the reported adverse events is prepared and submitted to the DSMB.

### Frequency and plans for auditing trial conduct

The DFG (German Research Foundation) reserves the right to audit the study if necessary.

### Plans for communicating important protocol amendments to relevant parties (e.g., trial participants, ethical committees)

Protocol amendments must be approved by the ethics committee of the Charité – Universitätsmedizin Berlin and the ethics commission of the state medical association Brandenburg. Changes are communicated to the funding body. The protocol will also be updated in the clinical trial registry (DRKS). Changes are communicated to all study personnel and study materials will be updated.

### Dissemination plans

The results will be disseminated through international peer-reviewed articles in open access journals. The authorship guideline is available from the principal investigator upon reasonable request. The SPIRIT and CONSORT statements, with extensions for pragmatic trials, will be used for reporting of the protocol and trial, respectively. Further dissemination measures will include presentations at local and international conferences,

An anonymized dataset will be made available (*Plans to give access to the full protocol, participant level-data and statistical code {31c}*). The link worker training concept is available through Zenodo [48].

## Discussion

This trial will assess the feasibility of implementing SP in the German primary care setting and will provide information and data necessary to plan a confirmatory trial (e.g., recruitment and trial processes, feasibility of individual randomization vs. cluster randomization, suitability of endpoints, sample size calculation). Implementing SP into routine care could equip GPs with an adequate solution to address psychosocial problems.

### Trial status

Recruitment started on September 16^th^, 2024. Recruitment is expected to close by May 31^st^, 2025. Trial recruitment will continue until the sample size of 300 study participants will be recruited. Data collection is expected to be finished November 30^th^ 2025. Results are planned to be available May 31^st^ 2026.

This study protocol is version 1.2 from February 12^th^, 2024.

## Supporting information

S1 File Prüfplan V1.2 vom 12.02.2025 ohne Logo. (PDF)

S2 File Study Protocol V1.2 from 12/02/2025 without logo automated English translation. (PDF)

S1 Data SocPres_SPIRIT_checklist_V1-0_2025_02_14. (DOCX)

## Abbreviations

CRF: Case Report Form
DFG: German Research Foundation (Deutsche Forschungsgemeinschaft)
DRKS: German Clinical Trials Register (Deutsches Register Klinischer Studien)
DSMB: Data Safety Monitoring Board
GBO: Goal based outcome
GP: General Practitioner
PROMs: Patient reported outcomes
RedCap: Research Electronic Data Capture
SAP: statistical analysis plan
SP: social prescribing
TAU+: treatment-as-usual plus brochure with information about local offers for non-clinical support and services in the community
