# Supplementary material for: Social prescribing to improve health and well-being of patients presenting with non-medical health related social needs in primary care: Study protocol of a multi-center randomized controlled pragmatic feasibility trial
Source: PLoS One. 2025 May 23;20(5):e0322372. doi: 10.1371/journal.pone.0322372 (PMC12101629; doi:10.1371/journal.pone.0322372)
Supplement: S1 File — (PDF) [file pone.0322372.s001.pdf]

Prüfplan

Das „Soziale Rezept“ zur Verbesserung von Gesundheit und Wohlbefinden von Patient:innen, die sich mit nicht-medizinischen gesundheitsbezogenen sozialen Problemen in der hausärztlichen Versorgung vorstellen: eine multizentrische, randomisiert kontrollierte, pragmatische Machbarkeitsstudie

„Soziales Rezept“

V1.2 vom 12.02.2025

(Version ohne Logo)

Inhaltsverzeichnis

1. Allgemeine Informationen zur Studie ... 5
1.1 Projekttitel, Versionsnummer, Versionsdatum, Versionsverlauf ... 5
1.2 Verantwortlichkeiten ... 6
1.3 Prüfzentren und Hauptprüfer ... 7
1.4 Finanzierung ... 8
1.5 Registrierung in einem öffentlich zugänglichen Studienregister ... 8
1.6 Studiensynopse ... 9
2 Hintergrund ... 15
3 Projektziele und Endpunkte ... 16
4 Studiendesign ... 19
4.1 Studiendesign ... 19
4.2 Begründung des Studiendesigns ... 20
4.3 Intervention ... 21
4.4 Kontrolle ... 23
4.5 Maßnahmen zur Reduktion von Bias ... 23
4.5.1 Randomisierung und Allokation ... 23
4.5.2 Verblindung ... 24
4.6 Studienteilnehmende und Zentren ... 24
4.6.1 Ein- und Ausschlusskriterien für Prüfzentren ... 24
4.6.2 Ein- und Ausschlusskriterien für Patient:innen ... 24
4.7 Patientenpfad und Visiten ... 25
4.7.1 t_{-1}: Konsultation in der Praxis ... 28
4.7.2 t_0: Baseline-Datenerhebung und Randomisierung ... 29
4.7.3 t_1 Follow-Up-Befragung nach drei Monaten ... 30
4.7.4 t_2 Follow-Up-Befragung nach sechs Monaten ... 30
4.8 Medizinische Versorgung ... 30
5 Abbruchkriterien ... 30
5.1 Abbruchkriterien für individuelle Studienteilnehmende ... 30
5.2 Abbruchkriterien für Studienteile oder die gesamte Studie ... 31
6 Datenerhebung ... 31
6.1 Datenquellen ... 31
6.1.1 Personenidentifizierende Daten ... 31
6.1.2 Gesundheitsdaten ... 31
6.1.3 Befragungsdaten der Hausärzt:innen ... 31
6.1.4 Rekrutierungsliste ... 31

6.1.5 Unerwünschte Ereignisse ... 31
6.1.6 Befragungsdaten der Patient:innen ... 32
6.1.7 Dokumentationen der Link Worker ... 32
6.2 Daten mit und ohne Quelldaten ... 32
6.3 Archivierung und Zugang zu Quelldaten ... 32
6.4 Instrumente ... 32
7 Datenschutz ... 34
7.1 Datenschutzkonzept ... 34
7.2 Ansprechpartner:innen für Betroffenenrechte ... 34
8 Biometrie ... 34
8.1 Allgemein ... 34
8.2 Fallzahlschätzung ... 35
8.3 Auswertungspopulationen ... 35
8.4 Analysen ... 35
8.5 Sensitivitätsanalysen ... 36
9 Datenmanagement ... 36
9.1 Erfassung und Speicherung der Daten ... 37
9.2 Pseudonymisierung ... 37
9.3 Rechte der Teilnehmenden (Widerruf, Datenlöschung, Recht auf Auskunft, Berichtigung) ... 37
10 Qualitative Prozessevaluation ... 38
10.1 Ziele und Themen der qualitativen Prozessevaluation ... 38
10.2 Rekrutierung für die qualitative Prozessevaluation ... 39
10.3 Datenerhebung, Datenspeicherung und Datenauswertung für die qualitative Prozessevaluation ... 40
11 Begleitende gesundheitsökonomische Evaluation ... 41
12 Qualitätsmanagement und -sicherung ... 41
12.1 Standard Operating Procedures (SOPs) ... 42
12.2 Monitoring ... 42
13 Sicherheit ... 42
13.1 Unerwünschte Ereignisse ... 42
13.1.1 Definition unerwünschter Ereignisse ... 43
13.1.2 Meldung unerwünschter Ereignisse ... 43
13.1.3 Zentrale Dokumentation unerwünschter Ereignisse ... 44
13.1.4 Zweitbewertung unerwünschter Ereignisse ... 44
13.2 Berichterstattung ... 44
13.3 Data Safety Monitoring Board (DSMB) ... 44
14 Ethische Erwägungen ... 45

14.1 Individueller Nutzen ... 45
14.2 Gruppennutzen ... 45
14.3 Nutzen für teilnehmende Prüfzentren ... 46
14.4 Schaden/Risiko ... 46
14.5 Zusammenfassende Bewertung ... 46
14.6 Zuständige Ethikkommissionen ... 47
15 Versicherung der Studienteilnehmenden ... 47
16 Publikationsleitlinie ... 47
17 Referenzen ... 48
18 Abkürzungsverzeichnis ... 51
19 Abbildungsverzeichnis ... 52
20 Tabellenverzeichnis ... 53
21 Unterschriften ... 54

1. Allgemeine Informationen zur Studie

1.1 Projekttitel, Versionsnummer, Versionsdatum, Versionsverlauf

Titel

Das „Soziale Rezept“ zur Verbesserung von Gesundheit und Wohlbefinden von Patient:innen, die sich mit nicht-medizinischen gesundheitsbezogenen sozialen Problemen in der hausärztlichen Versorgung vorstellen: eine multizentrische, randomisiert kontrollierte, pragmatische Machbarkeitsstudie

Kurztitel

„Soziales Rezept“

Versionsnummer

1.2

Versionsdatum

12.02.2025

Version	Änderungsgrund bzw. Kurzbeschreibung (Abschnitt) der Änderungen
V1.0	Ersterstellung
V1.1	Zufriedenheitsmessung auch nach drei Monaten und beide Arme (3); Goal Basiertes Outcome auch durch Link Worker (4.3); Detailliertere Klassifizierung sozialer Probleme (4.6.2); Anpassung Antwortkategorien subjektiver Gesundheitszustand (6.4)
1.2	Zwei weitere Prüfzentren eingefügt: Hausarztpraxis Neukölln (Hauptprüferin Jihan Saeed), Praxis Weichselstraße (Hauptprüferin: Dr. med. Anja Zitscher). Ausschlusskriterium „eine im selben Haushalt lebende Person ist bereits in die Studie eingeschlossen“ eingefügt. Ausschlusskriterium „rechtlich betreut“ spezifiziert in „in Bezug auf die Gesundheit rechtlich betreut“.

1.2 Verantwortlichkeiten

Sponsor	Charité – Universitätsmedizin Berlin<br>Charitéplatz 1<br>10117 Berlin
Studienleitung/Sponsorvertretung	Prof. Dr. med. Wolfram Herrmann<br>Institut für Allgemeinmedizin<br>Charité – Universitätsmedizin Berlin<br>Charitéplatz 1<br>10117 Berlin
Stellvertretende Studienleitung	Dr. med. Hendrik Napierala M.Sc.<br>Institut für Allgemeinmedizin<br>Charité – Universitätsmedizin Berlin<br>Charitéplatz 1<br>10117 Berlin
Verantwortliche Biometrikerin	PD Dr. Stephanie Roll<br>Institut für Sozialmedizin, Epidemiologie und Gesundheitsökonomie<br>Charité – Universitätsmedizin Berlin<br>Charitéplatz 1<br>10117 Berlin

1.3 Prüfzentren und Hauptprüfer

Gemeinschaftspraxis für Allgemeinmedizin
Dr. med. Kathrin Hecker
Landsberger Allee 44
10249 Berlin

Ärzte am Hermannplatz
Gerd Michels
Kottbusser Damm 72
10967 Berlin

MVZ Praxis Korok
Rheinsteinstr. 1
10318 Berlin

Hausarztpraxis Lichtenberg
Iris Boehmer
Möllendorffstr. 45
10367 Berlin

Gesundheitszentrum Haus Nazareth
Dr. med. Burghard Storm
Wrangelstr. 6/7
12165 Berlin

Hausarztpraxis Dashti
Dr. med. Hiwa Dashti
Am Paschenberg 30
16225 Eberswalde

Hausarzt Dr. Zerbaum & Kollegen MVZ
Dr. med. Mario Zerbaum
Petersilienstraße 12
14776 Brandenburg

Hausarztpraxis Neukölln
Jihan Saeed
Hermannstraße 52
12049 Berlin

Praxis Weichselstraße
Dr. med. Anja Zitscher
Weichselstr. 59
12045 Berlin

1.4 Finanzierung

Die Studie wird durch die Deutsche Forschungsgemeinschaft im Programm "Klinische Studien" als Machbarkeitsstudie finanziert.

Antragsnummer: HE 6399/3-1

Projektnummer: 530364906

Deutsche Forschungsgemeinschaft e.V. (DFG)

Kennedyallee 40

53175 Bonn

1.5 Registrierung in einem öffentlich zugänglichen Studienregister

Deutsches Register Klinischer Studien (DRKS)

DRKS-ID: DRKS00034654

1.6 Studiensynopse

Tabelle 1: Synopse

Studienttitel (Deutsch)	Das „Soziale Rezept“ zur Verbesserung von Gesundheit und Wohlbefinden von Patient:innen, die sich mit nicht-medizinischen gesundheitsbezogenen sozialen Problemen in der hausärztlichen Versorgung vorstellen: eine multizentrische, randomisiert kontrollierte, pragmatische Machbarkeitsstudie
Title of trial (English)	Social prescribing to improve health and well-being of patients presenting with non-medical health related social needs in primary care: a multi-center randomized controlled pragmatic feasibility trial
Indikation	Nicht-medizinische gesundheitsrelevante Probleme (International Classification of Primary Care 3rd Edition, ICPC-3: ZC01-ZC99 “Soziale Probleme”)
Ziel	Das Hauptziel dieser Machbarkeitsstudie ist es, die Durchführbarkeit einer randomisiert-kontrollierten Studie zum "Sozialen Rezept" in Deutschland zu bewerten und Informationen und Daten zu liefern, die für die Planung einer konfirmatorischen Studie erforderlich sind (z. B. Rekrutierungs- und Studienverfahren, Durchführbarkeit der individuellen Randomisierung im Vergleich zur Cluster-Randomisierung, Eignung der Endpunkte, Berechnung der Stichprobengröße).
Studiendesign	Multizentrische, zweiarmige, randomisierte (2:1), kontrollierte, parallele, offene, pragmatische, explorative Machbarkeitsstudie mit Mixed-Methods Prozessevaluation
Studienpopulation	N (total) = 215 (Intervention n= 143; Kontrolle n= 72)
Ein-/Ausschlusskriterien	**Praxen:**<br><br>Einschlusskriterien von Hauptprüfer:in je Prüfzentrum: Hausärzt:in (Fachärzt:in für Allgemeinmedizin oder Innere Medizin mit

	hausärztlicher Versorgung, Praktische:r Ärzt:in) mit 80 h Grundausbildung in "Psychosomatischer Grundversorgung"<br><br>Ausschlusskriterien: Praxen mit mehr als 50 % Versorgungsvolumen in spezialisierter Versorgung (z. B. Spezialisierte ambulante Palliativversorgung, Infektiologie, Suchttherapie, Psychotherapie)<br><br>**Teilnehmende:**<br><br>Einschlusskriterien: Einwilligungsfähige erwachsene (18 Jahre und älter) Patient:innen, die sich mit einem oder mehreren nicht-medizinischen gesundheitsbezogenen sozialen Problemen in der hausärztlichen Praxis vorstellen.<br><br>Ausschlusskriterien: Patient:innen, die in Bezug auf die Gesundheit rechtlich betreut sind, eine im selben Haushalt lebende Person ist bereits in die Studie eingeschlossen
Intervention	**Intervention:**<br><br>Die Intervention beginnt mit der Überweisung von Patient:innen mit nicht-medizinischen gesundheitsbezogenen sozialen Problemen durch Hausärzt:innen an eine:n Link Worker. Die Hauptaufgabe der Link Worker liegt darin, die teilnehmenden Patient:innen darin zu unterstützen, sich mit vorhandenen, lokal verfügbaren nicht-klinischen Unterstützungsangeboten in Verbindung zu setzen, die helfen können, die nichtmedizinischen gesundheitsbezogenen Probleme (z.B. Einsamkeit oder finanzielle Sorgen) zu verbessern. In der Beratung werden gemeinsam mit den Teilnehmenden individuelle Aktionspläne erarbeitet und umgesetzt, die auf den Bedürfnissen und Zielen der Teilnehmenden beruhen und konkrete Maßnahmen zur Problemlösung beinhalten. Die Hausärzt:innnen werden mittels Feedback-Formular informiert, an welche Dienste in der Umgebung die Patient:innen weitervermittelt wurden. Der Zeitraum, in dem Link Worker und Teilnehmende Kontakt haben

	und die Anzahl der Kontakte ist in dieser Machbarkeitsstudie nicht begrenzt. Zu erwarten sind 2-5 Treffen in in einem Zeitraum von vier bis zwölf Wochen.<br><br>**Kontrolle:**<br><br>Übliche Versorgung plus Broschüre mit Informationen über lokale nicht-klinische Unterstützungsangebote und Dienstleistungen (“Treatment-as-usual +”).
Endpunkte	**Primäre Machbarkeitsendpunkte:**<br><br>Anteil der Teilnehmenden, die mindestens einen Termin mit dem Link Worker haben (nur Interventionsarm)<br><br>Anteil der Teilnehmenden, die die Studie vor der 6-monatigen Nachbeobachtung abbrechen (Loss to Follow-up, beide Arme)<br><br>**Sekundäre Machbarkeitsendpunkte:**<br><br>Akzeptanz:<br><br>Zufriedenheit mit der Intervention bei Hausärzt:innen (Fragebogen nach Last-Patient-Last-Visit) und<br><br>Teilnehmendenzufriedenheit (3-Monats-Follow-Up und 6-Monats-Follow-up)<br><br>Praktikabilität:<br><br>Eingeschätzter Zeitaufwand der Hausärzt:innen<br><br>Anteil der in Frage kommenden Patient:innen im Vergleich zur Anzahl der in der Praxis behandelten Patient:innen<br><br>Anteil der Patient:innen, die aufgrund einer Sprachbarriere ausgeschlossen wurden<br><br>Anteil der in Frage kommenden Patient:innen, die in die Studie einwilligen

	Anteil der einwilligenden Patient:innen, die randomisiert werden<br><br>Anteil der randomisierten Patient:innen, die vereinbarte Termine mit dem Link Worker wahrnehmen (nur Interventionsarm)<br><br>Bedarf/Nutzung (nur Interventionsarm):<br><br>Von Hausärzt:in eingeschätzter Bedarf für SP der Teilnehmenden<br><br>Von Link Worker eingeschätzter Bedarf für SP der Teilnehmenden<br><br>Anzahl der Termine mit Link Workern pro Teilnehmendem<br><br>Dauer der Termine mit Link Workern<br><br>Form der Interaktion mit dem Link Worker (persönlich, Hausbesuch, in der Hausarztpraxis, telefonisch)<br><br>Von Link Workern vermittelte Angebote<br><br>Inanspruchnahme der empfohlenen vermittelten Angebote<br><br>**Klinische Endpunkte/ Patient Reported Outcome Measures (PROM), nach 3 und 6 Monaten:**<br><br>Gesundheitszustand (WHODAS 2.0) (wichtigster klinischer Endpunkt im Zeitraum nach 6 Monaten)<br><br>Allgemeine Lebenszufriedenheit (L-1)<br><br>Einsamkeit (De Jong Gierveld Loneliness Scale Kurzversion)<br><br>Gesundheitsbezogene Lebensqualität (EQ-5D-5L)<br><br>Wohlbefinden (WHO-5, ICECAP-A)<br><br>Subjektiver Gesundheitszustand<br><br>Zielbasierter Endpunkt (Goal based outcome, GBO, nur Interventionsarm, Baseline-Erhebung bei Festlegung mit Link Worker)

	Inanspruchnahme von Gesundheits- und Sozialwesen (Ausschnitte aus PECUNIA RUM/EHIS-GeDA)<br><br>Auftreten unerwünschter Ereignisse (Anzahl der Todesfälle, Notaufnahmebesuche, ungeplanter Hospitalisierungen, neu aufgetretener Suizidalität, Suizidversuche, andere unerwünschte Ereignisse)
Fallzahl	Da es sich um eine explorative Machbarkeitsstudie ohne konfirmatorische Hypothesenprüfung handelt, wird keine formale Berechnung der Fallzahl durchgeführt. Aufgrund früherer Erfahrungen wird davon ausgegangen, dass die Analyse von insgesamt 215 Teilnehmern (im Verhältnis 2:1, d. h. n=143 im SP-Arm und n=72 im TAU+-Arm) ausreicht, um die Machbarkeits- und Akzeptanzaspekte der Studie deskriptiv zu bestimmen und weitere Informationen für die Planung anschließender Studien zu erhalten.<br><br>Es wird mit einem Drop-Out von ca. 30 % gerechnet, so dass die Follow-up-Daten für sekundäre klinische Endpunkte von ca. 150 Patienten (n = 100 im SP-Arm und n = 50 im TAU+-Arm) nach 6 Monaten zu Verfügung stehen.<br><br>Daraus ergibt sich:<br><br>Screening: n= 600<br><br>Rekrutierung: n= 300<br><br>Randomisierung: n=215<br><br>Auswertung: n=150-215
Statistik	Alle erhobenen Daten werden deskriptiv ausgewertet: Mittelwerte, Standardabweichung, Median und Quartil oder Häufigkeiten und Prozentsätze (insgesamt und getrennt nach Behandlungsgruppen).<br><br>Primäre Endpunkte: Der Anteil der Teilnehmenden in der Interventionsgruppe, die mindestens einen Termin mit dem Link Worker wahrgenommen haben, und der Anteil der Teilnehmenden,

	die die Studie vor der 6-monatigen Nachbeobachtung abbrechen (Abbruchrate), werden deskriptiv analysiert, mit Häufigkeiten und Prozenten, inkl. 95 % Konfidenzintervall pro Behandlungsgruppe. Der Vergleich der Abbrecherquote zwischen den Behandlungsgruppen erfolgt deskriptiv sowie mit Hilfe logistischer Regression, um die mögliche Einflussfaktoren zu bestimmen.<br><br>Sekundäre Endpunkte werden deskriptiv analysiert (Häufigkeiten/Prozente, Mittelwert/Standardabweichung oder Median/Interquartil je nach Skala und Verteilung, jeweils mit entsprechenden 95%-Konfidenzintervallen).<br><br>Vergleiche zwischen den Behandlungsgruppen werden mittels logistischer Regression (binäre Endpunkte) oder Kovarianzanalyse (kontinuierliche Endpunkte, adjustiert für den jeweiligen Baselinewert, sofern verfügbar) durchgeführt, wobei jeweils das Zentrum berücksichtigt wird.<br><br>Sicherheit: Unerwünschte Ereignisse werden deskriptiv analysiert (Häufigkeiten und Prozente pro Behandlungsgruppe).<br><br>Grundsätzlich werden alle Ergebnisse explorativ interpretiert.
Qualitative Prozessevaluation	Die Prozessevaluation besteht aus einer kontinuierlichen quantitativen Analyse der Studiendokumentation sowie aus qualitativen Interviews mit den beteiligten Akteuren (Patient:innen, Hausärzt:innen, Link Worker, Verteter:innen lokaler Angebote, Studienteam). Insgesamt sollen 30-40 Teilnehmende rekrutiert werden, davon zur Hälfte Teilnehmende der beiden Studienarme und zur Hälfte andere beteiligte Akteure. Die Interviews werden nach Thematischen Kodieren ausgewertet und mit den quantitativen Analysen trianguliert.
Studiendauer	**Gesamtstudiendauer:** 24 Monate<br><br>**First patient in to last patient out:** 12 Monate

	**Rekrutierungsdauer:** 6 Monate<br><br>**Interventionsdauer:** 3 Monate
Finanzierung	Deutsche Forschungsgemeinschaft: Antragsnummer HE 6399/3-1; Projektnummer 530364906

2 Hintergrund

Nicht-medizinische gesundheitsrelevante soziale Probleme sind in der Primärversorgung weit verbreitet (1). Sie können einen Einfluss auf das Auftreten von psychischen und somatischen Erkrankungen haben (2–4). Solche sozialen Probleme können jedoch auch durch (chronische) Krankheiten verursacht werden. Sie sind mit erheblichen wirtschaftlichen Kosten verbunden, z. B. durch kurzfristige Arbeitsunfähigkeit und langfristige Abwesenheit vom Arbeitsplatz (5–7). Nicht-medizinische gesundheitsbezogene soziale Probleme werden durch die International Classification of Primary Care 3. Edition (ICPC-3) im Kapitel ZC: "Soziale Probleme" operationalisiert (8) und umfassen Probleme wie Einsamkeit, Probleme in der Familie und am Arbeitsplatz sowie finanzielle Schwierigkeiten (1,9). Diese Probleme betreffen überproportional Menschen mit einem niedrigeren sozioökonomischen Status und verstärken damit gesundheitliche Ungleichheiten (10).

Obwohl nicht-medizinische gesundheitsrelevante soziale Probleme in der Allgemeinmedizin weit verbreitet sind, können Allgemeinmediziner:innen bei ihren Konsultationen nur wenige Probleme ansprechen und/oder klären (11). In Deutschland gibt es jedoch ein umfangreiches Angebot an nicht-klinischen Unterstützungsangeboten und Dienstleistungen in der Nachbarschaft. Dennoch gibt es keine formalisierten Verbindungen zwischen diesen Diensten und der Primärversorgung (12). Daher ist es sowohl für Hausärzt:innen als auch für Patient:innen schwierig, die richtige Anlaufstelle zu finden. Hier bieten sich niedrigschwellige, kooperative Lösungen an (1).

Wissenschaftlich werden mehrere mögliche Lösungsansätze diskutiert: z.B. integrierte Primärversorgungszentren, Sozialarbeiter:innen in der Primärversorgung und Social Prescribing (SP, „Soziales Rezept“)). SP wurde in Großbritannien entwickelt und wurde in den letzten Jahren im National Health System (NHS) flächendeckend implementiert. Weltweit wurden SP-Programme in mehreren Ländern wie Kanada, Österreich und Singapur eingeführt (13). SP bietet Hausärzt:innen eine nicht-medizinische Überweisungsoption, die bestehende Behandlungen begleiten kann, um Gesundheit und Wohlbefinden zu verbessern (14). SP wird durch die Einbindung eines "Link

Workers" umgesetzt, an den Patienten mit nicht-medizinischen gesundheitsrelevanten sozialen Problemen verwiesen werden (= Ausstellung eines "sozialen Rezepts"). Link Worker sind speziell geschulte Fachkräfte oder Ehrenamtliche. Gemeinsam mit den Patient:innen erarbeiten Sie sich einen Aktionsplan und vermitteln diese an vorhanden Angebote vor Ort. Die aktuelle Evidenz beruht überwiegend auf kontrollierten Vorher-Nachher Studien, es gibt nur vereinzelte kontrollierte oder randomisiert-konrtrollierte Studien (16).

Aus der vorhandenen Evidenz ergibt sich die Notwendigkeit randomisierter kontrollierter Studien zur Bewertung der Wirksamkeit von SP - insbesondere außerhalb Großbritanniens. Da es jedoch nur begrenzte Erfahrungen mit der Etablierung von SP in Deutschland gibt, ist eine Machbarkeitsstudie notwendig, um verschiedene Aspekte der Studie zu klären. Wichtige Punkte sind die Akzeptanz der Studie, der Drop-out insbesondere in der Kontrollgruppe, der Umfang der Beratung durch den Link Worker im deutschen Gesundheits- und Sozialsystem und die Durchführbarkeit der Endpunktmessungen.

3 Projektziele und Endpunkte

Das Hauptziel dieser Machbarkeitsstudie ist es, die Durchführbarkeit einer randomisiert-kontrollierten Studie zum "Sozialen Rezept" (Social Prescribing) in Deutschland zu bewerten und Informationen und Daten zu liefern, die für die Planung einer konfirmatorischen Studie erforderlich sind (z.B. Rekrutierungs- und Studienverfahren, Durchführbarkeit der individuellen Randomisierung im Vergleich zur Cluster-Randomisierung, Berechnung der Stichprobengröße).

Die sekundären Ziele der Studie umfassen die Bewertung von Akzeptanz, Praktikabilität und Bedarf/Nutzung der Intervention, da die Studie eine Pilotierung von Social Prescribing in Deutschland darstellt und diese Parameter entscheidend für die Gestaltung zukünftiger Angebote sind. Die Studie soll zudem die Eignung verschiedener Endpunkte überprüfen, die zukünftig über die gesundheitlichen Effekte von Social Prescribing auf der individuellen und gesundheitsökonomischen Ebene informieren können. Darüber hinaus werden im Rahmen der Studie auftretende unerwünschte Ereignisse systematisch erfasst, um Aussagen über die Sicherheit von Social Prescribing treffen zu können.

Die folgende Tabelle gibt die Ziele der Studie mit den zugeordneten quantitativen Endpunkten wieder.

Tabelle 2: Projektziele und Endpunkte

Ziele	Endpunkte
**Hauptziel**	**Primäre Endpunkte**
Bestimmung der Machbarkeit und Durchführbarkeit einer randomisiert-kontrollierten Studie zum "Sozialen Rezept"	Anteil der Teilnehmenden, die mindestens einen Termin mit dem Link Worker haben (nur Interventionsarm, innerhalb von 6 Monaten)
	Anteil der Teilnehmenden, die die Studie vor der 6-monatigen Nachbeobachtung abbrechen (Abbruchrate, beide Arme)
**Sekundäre Ziele**	**Sekundäre Endpunkte**
Bestimmung der Akzeptanz	Zufriedenheit der Hausärzt:innen (Fragebogen nach Last-Patient-Last-Visit für alle teilnehmenden Hausärzt:innen)
	Teilnehmendenzufriedenheit (3-Monats-Follow-Up und 6-Monats-Follow-up)
Bestimmung der Praktikabilität	Eingeschätzter Zeitaufwand der Hausärzt:innen (Fragebogen nach Last-Patient-Last-Visit für alle teilnehmenden Hausärzt:innen)
	Anteil der in Frage kommenden Patient:innen im Vergleich zur Anzahl der in der Praxis behandelten Patient:innen
	Anteil der Patient:innen, die aufgrund einer Sprachbarriere ausgeschlossen wurden
	Anteil der in Frage kommenden Patient:innen, die in die Studie einwilligen
	Anteil der Teilnehmenden, die randomisiert wurden, die mit den Link Workern vereinbarte Termine wahrnehmen (nur Interventionsarm)
Bestimmung des Bedarfs und der Nutzung	Von Hausärzt:in eingeschätzter Bedarf für SP der Teilnehmenden (nur Interventionsarm)
	Von Link Worker eingeschätzter Bedarf für SP der Teilnehmenden (nur Interventionsarm)
	Anzahl der Konsultationen mit Link Worker pro Teilnehmendem (nur Interventionsarm)

	Dauer der Konsultationen mit Link Worker (nur Interventionsarm)
	Form der Interaktion mit Link Worker (persönlich, Hausbesuch, in der Praxis, telefonisch) (nur Interventionsarm)
	Von Link Worker vermittelte Angebote (nur Interventionsarm)
	Inanspruchnahme der empfohlenen vermittelten Angebote/der Broschüre durch die Teilnehmenden
Vergleich Soziales Rezept vs. TAU+ in klinischen Endpunkten/ Patient Reported Outcome Measures (PROM)	Gesundheitszustand (WHODAS 2.0) (3-Monats-Follow-Up und 6-Monats-Follow-up im Vergleich zu Baseline)
	Psychisches Wohlbefinden (WHO-5) (3-Monats-Follow-Up und 6-Monats-Follow-up im Vergleich zu Baseline)
	Allgemeine Lebenszufriedenheit (L-1) (3-Monats-Follow-Up und 6-Monats-Follow-up im Vergleich zu Baseline)
	Einsamkeit (De Jong Gierveld Loneliness Scale) (3-Monats-Follow-Up und 6-Monats-Follow-up im Vergleich zu Baseline)
	Zielbasierter Endpunkt (GBO) (Baseline-Erhebung bei Festlegung mit Link Worker, 3-Monats-Follow-Up, 6 Monats-Follow-Up, nur Interventionsarm)
	Capability Well-being (ICECAP-A) (3-Monats-Follow-Up und 6-Monats-Follow-up im Vergleich zu Baseline)
	Gesundheitsbezogene Lebensqualität (EQ-5D-5L) (3-Monats-Follow-Up und 6-Monats-Follow-up im Vergleich zu Baseline)
	Subjektiver Gesundheitszustand (3-Monats-Follow-Up und 6-Monats-Follow-up im Vergleich zu Baseline)
Vergleich Soziales Rezept vs. TAU+ in	Inanspruchnahmeverhalten von gesundheitsbezogenen Leistungen, insbesondere die Domänen: Krankenhausaufenthalte und stationäre

gesundheitsökonomischen Endpunkten	Betreuungseinrichtungen, Notfallversorgung, ambulante Gesundheitsleistungen und soziale Dienste, Medikation, informelle Pflege, Arbeitsunfähigkeit (3-Monats-Follow-Up und 6-Monats-Follow-up im Vergleich zu Baseline)
Bestimmung der Sicherheit	Anzahl der Todesfälle bei Teilnehmenden
	Anzahl der Notaufnahmebesuche bei Teilnehmenden
	Anzahl der ungeplanten Hospitalisierungen bei Teilnehmenden
	Anzahl der Teilnehmenden mit (neu aufgetretener) Suizidalität
	Anzahl der Suizidversuche bei Teilnehmenden
	Anzahl der Teilnehmenden, bei denen andere unerwünschte Ereignisse aufgetreten sind

4 Studiendesign

4.1 Studiendesign

Die Studie ist als multizentrische, zweiarmige offene, explorative, individuell randomisierte, kontrollierte, pragmatische Machbarkeitsstudie konzipiert. SP wird mit „Treatment-as-usual“ plus Broschüre mit Informationen zu lokalen Angeboten für außerklinische Unterstützung und Dienstleistungen in der Gemeinde (TAU+) verglichen (vgl. Abb. 1).

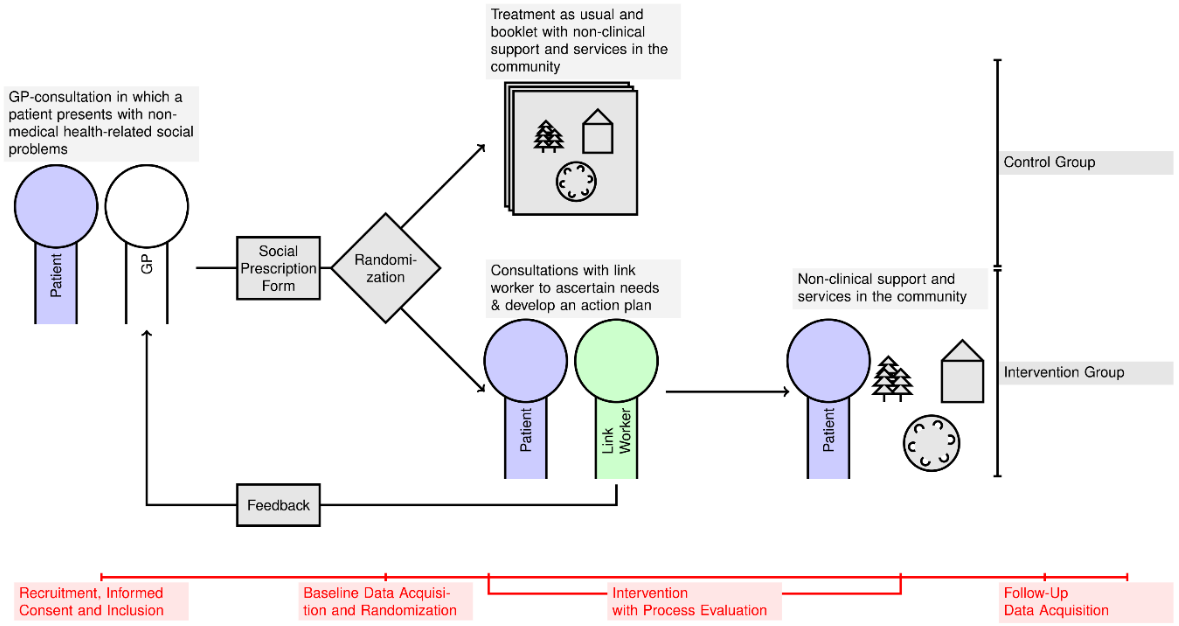

Abbildung 1: Studiendesign

4.2 Begründung des Studiendesigns

Wir haben uns für einen multizentrischen Ansatz entschieden, um die erforderliche Stichprobengröße zu erreichen und eine bessere Verallgemeinerbarkeit der Ergebnisse zu ermöglichen. In dieser Machbarkeitsstudie befinden sich die Studienzentren in Berlin und Brandenburg. Ein randomisiertes Design ist notwendig, um die Machbarkeit einer individuellen Randomisierung in der Primärversorgung zu testen. Wir haben uns für ein Zuteilungsverhältnis von 2:1 entschieden, um sicherzustellen, dass genügend Teilnehmer im Interventionsarm für die wichtigsten Machbarkeitsendpunkte verfügbar sind. Eine Kontrollgruppe ist erforderlich, um Informationen über die Machbarkeit der Randomisierung, des vorgeschlagenen Kontrollgruppendesigns und der Studienprozeduren bereitzustellen, insbesondere im Hinblick auf den Loss-to-follow-up in der Kontrollgruppe. Die Kontaminationen zwischen Interventions- und Kontrollgruppe innerhalb der einzelnen Praxen werden qualitativ analysiert, um die Machbarkeit einer individuellen Randomisierung im Vergleich zur Cluster-Randomisierung zu beurteilen.

Es wurde ein möglichst pragmatischer Ansatz gewählt, da die Absicht der künftigen konfirmatorischen Studie darin bestehen wird, reale klinische Entscheidungen zur Implementierung von SP in die routinemäßige Primärversorgung zu treffen (17).

4.3 Intervention

Die dem Interventionsarm zugeteilten Patient:innen erhalten als Intervention ein Beratungsangebot von einem im Rahmen der Studie am Institut für Allgemeinmedizin angestellten Link Worker.

Link Worker sind Fachkräfte, die mindestens eine Ausbildung oder einen Bachelor-Abschluss in einem Bereich des Gesundheitswesens, der Gesundheitsfürsorge, des Sozialwesens oder der Sozialpflege haben. Die Link Worker werden vor Beginn der Intervention vom Studienteam in Anlehnung an bereits bestehende Link Worker-Schulungen aus Österreich und dem Vereinigten Königreich (18,19) geschult.

Die Hauptaufgabe der Link Worker liegt darin, die Patient:innen darin zu unterstützen, sich mit vorhandenen, lokal verfügbaren nicht-klinischen Unterstützungsangeboten in Verbindung zu setzen, die helfen können, die nichtmedizinischen gesundheitsbezogenen Probleme der Patient:innen (z.B. Einsamkeit oder finanzielle Sorgen) zu lösen. In der Beratung werden gemeinsam mit den Patient:innen individuelle Aktionspläne erarbeitet und umgesetzt, die auf den Bedürfnissen und Zielen der Patient:innen beruhen und konkrete Maßnahmen zur Problemlösung beinhalten. Dafür recherchieren die Link Worker neben ihrer Beratungstätigkeit lokale Angebote, tragen sie in eine elektronische Datenbank ein und vernetzen sich. Den Link Workern wird eine Supervision durch Mitarbeitende des Instituts für Allgemeinmedizin angeboten.

In einem ersten Schritt bewertet der/die Link Worker die Bedürfnisse, vereinbart ein Ziel und entwickelt gemeinsam mit dem Patienten/der Patientin einen Aktionsplan. Dieser Aktionsplan enthält Verweise auf bestehende nicht-klinischen Unterstützungsangeboten und Dienstleistungen in der Nachbarschaft. Der Link Worker unterstützt den Patienten/die Patientin dabei, die vereinbarten Dienstleistungen in Anspruch zu nehmen. Am Ende der Intervention gibt der Link Worker dem/der verschreibenden Hausarzt/Hausärztin ein Feedback (15). Der Kontakt zwischen dem Link Worker und dem Patienten/der Patientin umfasst in der Regel mehrere Konsultationen über mehrere Wochen hinweg.

Der Ersttermin mit dem Link Worker wird direkt im Anschluss an die telefonische Baseline-Befragung durch die Study Nurse mit dem Patienten vereinbart. Der Ersttermin soll spätestens 14 Tage nach der Rekrutierung in den Studienarm erfolgen. Dauer und Anzahl der Termine sind im Rahmen dieser Machbarkeitsstudie nicht limitiert und sind abhängig vom Bedarf der Patient:innen und der Kapazität der Link Worker. Wir rechnen mit durchschnittlich 2-5 Terminen pro Patient:in mit einer Dauer von bis zu 45 Minuten über einen Zeitraum von vier bis 12 Wochen. Die Beratungstermine finden in der Regel in Räumlichkeiten der Prüfzentren statt. Auf Wunsch der Patient:innen können Beratungen bei Bedarf auch telefonisch oder per Hausbesuch erfolgen.

Im Rahmen der Beratung können Link Worker nach ausdrücklicher Zustimmung der Patient:innen (konkludente Einwilligung) Daten der Patient:innen an lokale Angebote weitergeben (z.B. zur Terminvereinbarung). Nach Abschluss der Beratung durch den Link Worker erhalten die behandelnden Hausärzt:innen über ein standardisiertes Formular eine Rückmeldung über die Beratung.

Die folgende Tabelle beschreibt die Intervention des Sozialen Rezepts (Social Prescribing) gemäß des TIDieR Frameworks.

Tabelle 3: Intervention nach dem TIDieR Framework

Aspekt		Begründung
**Kurzname**	Soziales Rezept	
**Warum**	Durch Social Prescribing können Patienten mit nicht-medizinischen, gesundheitsbezogenen sozialen Problemen an nicht-klinische Hilfsangebote und Dienste in der Gemeinde verwiesen werden.	Dabei wird auf bereits vorhandene Dienste zurückgegriffen.
**Was**	Für jede Gemeinde/Nachbarschaft wird der Link Worker eine Datenbank einrichten, die Informationen und Kontaktdaten zu den für die soziale Verschreibung erforderlichen Diensten enthält. Diese Datenbank baut auf Informationen auf, die in den im Kontrollarm verwendeten Broschüren bereitgestellt werden. Die Datenbank wird während der Intervention kontinuierlich erweitert.	Die Datenbank soll dabei helfen, eine nachhaltige Intervention aufzubauen.
	1. Der Hausarzt stellt ein individuelles Soziales Rezept aus, in dem die Probleme des Patienten dargestellt und die Hauptprobleme hervorgehoben werden.<br>2. Der Link Worker hat mehrere Termine mit dem Patienten. Zunächst wird der Bedarf des Patienten ermittelt, gemeinsam wird ein Hauptziel festgelegt und ein Aktionsplan entwickelt, an welche lokalen Unterstützungsmöglichkeiten der Patient vermittelt werden soll.<br>3. Der Link Worker sorgt dafür, dass der Patient zu den vereinbarten Unterstützungsmöglichkeiten vor Ort kommt.<br>4. Der Link Worker informiert den Hausarzt anhand eines standardisierten Feedback-Formulars darüber, wohin der Patient überwiesen wurde.	Dieses Konzept basiert auf der Definition von SP von Muhl et al. (14) und bestehenden SP-Programmen in Großbritannien.
**Wer**	Der Link Worker hat eine Ausbildung oder einen Bachelorabschluss aus dem Gesundheits- oder Sozialbereich und zusätzlich eine Schulung als Link Worker erhalten	Link Worker benötigen ein grundlegendes Verständnis der Gesundheits- und Sozialfürsorge.
**Wie**	Individuelle Konsultationen vorzugsweise persönlich oder alternativ telefonisch.	Da die Bedürfnisse sehr individuell sind, bedarf es maßgeschneiderter Leistungen.
**Wo**	Vorzugsweise in der Hausarztpraxis, alternativ als Hausbesuch oder telefonisch.	Dies ermöglicht mehr Flexibilität und entspricht den Bedürfnissen der Patienten.
**Wann und wie viel**	Das erste Beratungsgespräch findet spätestens zwei Wochen nach Studieneinschluss statt. Das erste Beratungsgespräch	Für die gefährdete Patientengruppe ist die Nachverfolgung besonders

	Aspekt	Begründung
	dauert maximal 45 Minuten. Der Link Worker und der Patient vereinbaren so viele Termine wie nötig, in der Regel in einem Zeitraum von bis zu vier Wochen nach Studieneinschluss, maximal bis zu zwölf Wochen.	wichtig, wenn sie die passenden lokalen Dienste erreichen. Daher muss eine Nachverfolgung ein integraler Bestandteil sein.

Die Durchführung der Intervention wird in der *SocPres_SOP Durchführung der Intervention* ausführlich beschrieben.

4.4 Kontrolle

Die Teilnehmenden der Kontrollgruppe erhalten eine Broschüre mit lokal verfügbaren nicht-klinischen Unterstützungsangeboten, die von der Study Nurse nach der Randomisierung in die Kontrollgruppe an die Teilnehmenden verschickt wird.

Dieser Prozess wird in der *SocPres_SOP Durchführung der Intervention* ausführlich beschrieben.

4.5 Maßnahmen zur Reduktion von Bias

4.5.1 Randomisierung und Allokation

Die Randomisierung in dieser Studie erfolgt, um die Machbarkeit der Randomisierung für die konfirmatorische Hauptstudie zu überprüfen. Das heißt die Randomisierung erfolgt nicht mit dem Ziel einer Testung von vergleichenden Hypothesen.

Der Randomisierungscode wird von einer unabhängigen Person des Instituts für Sozialmedizin, Epidemiologie und Gesundheitsökonomie (die nicht anderweitig an der Studie beteiligt ist) als Blockrandomisierung (mit variabler Blocklänge) stratifiziert nach Prüfzentrum (Hausarztpraxis) mit einem Zuteilungsverhältnis von 2:1 erstellt.

Alle Patient:innen, die ihre Zustimmung zur Teilnahme geben und die Einschlusskriterien erfüllen, werden nach der Baseline-Erhebung randomisiert. Die Randomisierungscodes werden von der für die Zuteilung verantwortlichen Study Nurse aus der REDCap-Datenbank entnommen, wobei die Zuteilung zur nächsten Patient:in verdeckt wird. Die Zuteilung erfolgt dementsprechend ohne jeglichen Einfluss der Studienleitung, der Prüfärzt:innen oder der Link Worker.

Das Vorgehen wird in der *SocPres_SOP Randomisierung und Allokation* beschrieben.

4.5.2 Verblindung

Aufgrund des Studiendesigns ist eine Verblindung der Prüfärzt:innen, der Link Worker und der Patient:innen nicht möglich. Wegen der 2:1 Randomisierung ist auch keine Verblindung der statistischen Analysen möglich.

4.6 Studienteilnehmende und Zentren

Für die Studie werden 300 erwachsene Patient:innen mit nicht-medizinischen gesundheitsbezogenen Problemen (s. Abschnitt 4.5.2) von ihren behandelnden Hausärzt:innen rekrutiert. Die Rekrutierung erfolgt in neun Prüfzentren.

4.6.1 Ein- und Ausschlusskriterien für Prüfzentren

Einschlusskriterien: Der Hauptprüfer des jeweiligen Prüfzentrums (Hausarztpraxis) besitzt folgende Qualifikationen:

- Hausärzt:in (Fachärzt:in für Allgemeinmedizin oder Innere Medizin mit hausärztlicher Versorgung, Praktische:r Ärzt:in)
- 80 h Grundausbildung in "Psychosomatischer Grundversorgung"

Ausschlusskriterien:

- Praxen mit mehr als 50 % Versorgungsvolumen in spezialisierter Versorgung (z. B. Spezialisierte ambulante Palliativversorgung, Infektiologie, Suchttherapie, Psychotherapie)

Die Ein- und Ausschlusskriterien auf Praxisebene sollen die Generalisierbarkeit auf die hausärztliche Versorgung sicherstellen.

4.6.2 Ein- und Ausschlusskriterien für Patient:innen

Einschlusskriterien:

- Einwilligungsfähige, erwachsene (18 Jahre und älter) Patient:innen
- Vorliegen ein oder mehrerer nicht-medizinische gesundheitsbezogene sozialer Probleme (s.u.)

Ausschlusskriterien:

- Patient:innen die in Bezug auf die Gesundheit rechtlich betreut sind
- eine im selben Haushalt lebende Person ist bereits in die Studie eingeschlossen

Nicht-medizinische gesundheitsbezogene soziale Probleme sind über das Kapitel ZC der International Classification of Primary Care - 3rd Revision (ICPC-3) definiert (8).

Die Klassifizierung umfasst Probleme in folgenden Bereichen:

- Finanzielle Probleme (inkl. Armut)
- Probleme mit Sozialleistungen (inkl. fehlender Bezug von Sozialleistungen, Probleme bei Arbeitsunfähigkeit)
- Probleme auf der Arbeit
- Arbeitslosigkeit als Problem
- Einsamkeit, soziale Isolation
- Probleme in der Beziehung mit dem Partner, mit einem Kind, mit den Eltern oder anderen Familienmitgliedern, mit sonstigen Personen (inkl. Mobbing)
- Verlust/Tod des Partners, eines Kindes, eines Elternteils oder anderen Familienmitglieds
- Probleme aufgrund der Krankheit des Partners, eines Kindes, eines Elternteils oder anderen Familienmitglieds
- Probleme bezogen auf Bildung, Schule, Ausbildung, oder Studium (inkl. Analphabetismus, fehlender Schulabschluss)
- Probleme aufgrund von Diskriminierung
- Probleme aufgrund des Aufenthaltsstatus/Aufenthaltstitel
- Probleme mit Gewalterfahrung
- Wohnungsprobleme (inkl. Wohnungslosigkeit oder drohender Wohnungslosigkeit)
- Rechtliche Probleme (z.B. drohende Gefängnisstrafe o.ä.)
- Probleme mit dem Gesundheitssystem (z.B. Pflege
- Andere soziale Probleme

4.7 Patientenpfad und Visiten

Die Visitenübersicht in Tabelle 4 und das CONSORT-Flussdiagramm (Abbildung 2) zeigen den individuellen Patientenpfad durch die Studie. Im Folgenden werden die einzelnen Zeitpunkte aus Tabelle 4 beschrieben.

Tabelle 4: Visitenübersicht

Visite	t_{-1}	t_0	s_1	$s_2, s_3, \ldots, s_n$	t_1	t_2
	**Konsultation in der Praxis**	**Baseline-Erhebung/ Randomisierung**	**Erste Link Worker Sitzung**	**Follow up Link Worker Sitzungen**	**3-Monats-Follow-Up**	**6-Monats-Follow-Up**
**Einschluss**						
Prüfung der Ein- und Ausschlusskriterien	X					
Aufklärung	X					
Einwilligung	X					
Soziodemografische Daten		X				
Randomisierung		X				
**Behandlung je Studienarm**						
TAU+ (Broschüre)		X				
Soziales Rezept		X	X	X	X	X
**Erhebungsinstrumente**						
Machbarkeitsendpunkte	X	X	X	X	X	X
WHODAS 2.0		X			X	X
WHO-5		X			X	X
L-1		X			X	X
ICECAP-A		X			X	X
EQ-5D-5L		X			X	X
De Jong Gierveld Loneliness Scale		X			X	X
Zielbasierter Endpunkt (Interventionsarm)			X		X	X

Inanspruchnahmeverhalten (PECUNIA-RUM)		X			X	X
Subjektiver Gesundheitszustand		X			X	X
Sicherheit		X	X	X	X	X

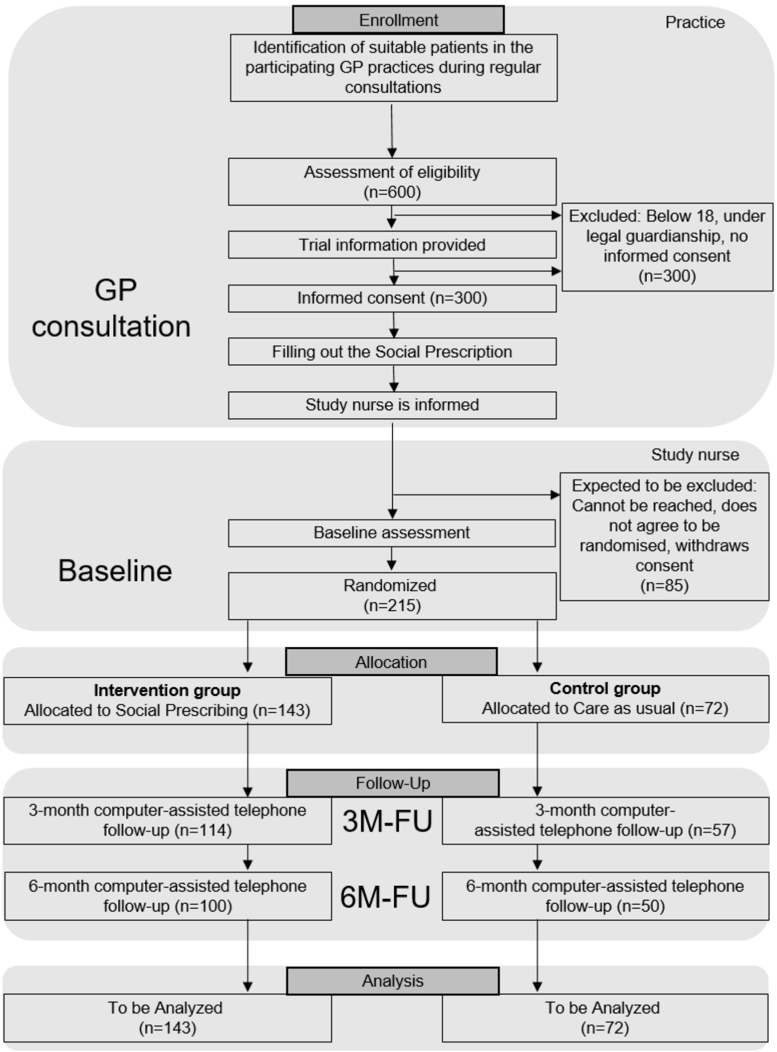

Abbildung 2: CONSORT-Flussdiagramm

4.7.1 t_{-1}: Konsultation in der Praxis

1. Ausgangspunkt ist, wenn in einem der Prüfzentren, in der normalen Arzt-Patienten-Konsultation mit einem der Prüfer ein nichtmedizinisches gesundheitsrelevantes soziales Problem zur Sprache kommt. Solche Probleme können beispielsweise sein:
    - Einsamkeit
    - finanzielle Probleme
    - Probleme auf der Arbeit
    - Probleme in der Familie

- Probleme mit der Wohnung/Wohnraum

2. In diesem Falle kann der Prüfer dem Patienten die Teilnahme an der Studie anbieten. Der Prüfer informiert den Patienten über die Studie und dokumentiert dies in seiner prüferspezifischen Rekrutierungsliste.
3. Wenn der Patient Interesse an einer Teilnahme an der Studie hat, kontrolliert der Prüfer die Ein- und Ausschlusskriterien der Studie. und klärt anhand der Studieninformation mündlich und schriftlich über die Studie auf. Der Patient erhält eine Teilnehmendeninformation ausgehändigt.
4. Der Patient erhält so viel Bedenkzeit wie er möchte. Das heißt er kann direkt in dieser Konsultation seine Einwilligung erklären oder zu einem späteren Zeitpunkt.
5. Wenn der Patient in die Teilnahme einwilligt, unterschreiben Patient und Prüfer die Einwilligungserklärung in doppelter Ausführung. Das Original verbleibt im Prüfzentrum. Der Patient erhält das Zweitexemplar der unterschriebenen Einwilligungserklärung.
6. Die Daten der eingeschlossenen Patienten werden dann von dem Prüfer an die Charité übermittelt. Es stehen verschiedene sichere Übermittlungswege bereit.

Die Details der Abläufe zu t_{-1} werden in der *SocPres_SOP Praxiskonsultation* ausführlich beschrieben.

4.7.2 t_0: Baseline-Datenerhebung und Randomisierung

1. Nach Eingang der Daten eines neuen Studienteilnehmers wird das Studienteam an der Charité automatisch informiert und eine Study Nurse übernimmt die Betreuung dieses Studienteilnehmers.
2. Die Study Nurse sollte innerhalb von 1-2 Wochen nach Einschluss Kontakt zu dem Studienteilnehmenden aufnehmen.
3. Zur Baseline-Datenerhebung führt die Study Nurse ein computergestütztes Telefoninterview (CATI) mit dem Studienteilnehmenden durch. Wenn dieses nicht möglich ist aufgrund von Einschränkungen auf Seiten des Teilnehmenden, kann die Baseline-Datenerhebung ausnahmsweise auch im Hausbesuch, in der Charité oder bei einem Praxisbesuch durch die Study Nurse erfolgen.
4. Nach Abschluss der Baseline-Datenerhebung führt die Study Nurse die zufällige Zuordnung zu einem Studienarm durch.
5. Werden Studienteilnehmende dem Interventionsarm zugeordnet, vereinbart die Study Nurse den ersten Termin mit dem Link Worker möglichst innerhalb von 7 Tagen nach der Randomisierung.

Werden Studienteilnehmende dem Kontrollarm zugeordnet, versendet sie an den Studienteilnehmer die Broschüre mit lokalen Unterstützungsangeboten.

Die Details der Abläufe zu t_0 werden in der *SocPres_SOP Datenerhebungen* ausführlich beschrieben.

4.7.3 t_1 Follow-Up-Befragung nach drei Monaten

Die erste Follow-Up Befragung erfolgt frühestens drei Monate nach der Randomisierung des Studienteilnehmers. Sie wird von der Study Nurse wieder als CATI oder im Ausnahmefall auch im Hausbesuch, in der Charité oder bei einem Praxisbesuch durchgeführt. Die Befragung erfolgt möglichst zeitnah an dem Dreimonatstermin, spätestens bis vier Monate nach Randomisierung.

Die Details der Abläufe zu t_1 werden in der *SocPres_SOP Datenerhebungen* ausführlich beschrieben.

4.7.4 t_2 Follow-Up-Befragung nach sechs Monaten

Die zweite Follow-Up Befragung erfolgt frühestens sechs Monate nach der Randomisierung des Studienteilnehmers. Sie wird von der Study Nurse wieder als CATI oder im Ausnahmefall auch im Hausbesuch, in der Charité oder bei einem Praxisbesuch durchgeführt. Die Befragung erfolgt möglichst zeitnah an dem Sechsmonatstermin, spätestens bis sieben Monate nach Randomisierung.

Die Details der Abläufe zu t_2 werden in der *SocPres_SOP Datenerhebungen* ausführlich beschrieben.

4.8 Medizinische Versorgung

Die medizinische Versorgung der Teilnehmenden durch ihre Hausärzt:innen bleibt unberührt von der Randomisierung in einen der beiden Studienarme und erfolgt ohne jegliche Einschränkungen über den gesamten Zeitraum der Studie hinweg.

5 Abbruchkriterien

5.1 Abbruchkriterien für individuelle Studienteilnehmende

1. Studienteilnehmende können jederzeit ohne Angabe von Gründen die Teilnahme an der Studie beenden.
2. Prüfer:innen können die Studie für eine teilnehmende Person abbrechen, wenn das Risiko für diese Person den individuellen Nutzen überwiegt.

5.2 Abbruchkriterien für Studienteile oder die gesamte Studie

1. Bei wiederholten Verstößen gegen den Prüfplan (bspw. Allokation) kann die Sponsorvertretung einzelne Prüfzentren schließen.
2. Das Data Safety and Monotoring Board (DSMB, s.u.) kann empfehlen, die Rekrutierung von Teilnehmenden entweder für alle oder für einige Behandlungsgruppen und/oder einige Untergruppen von Teilnehmenden zu stoppen. Die Sponsorvertretung entscheidet über einen Abbruch.

6 Datenerhebung

6.1 Datenquellen

6.1.1 Personenidentifizierende Daten

Von den Hausärzt:innen bereitgestellte personenidentifizierende Daten (persönliche Daten und Kontaktdaten der Teilnehmenden) werden vom Praxispersonal sicher an die Charité übertragen.

6.1.2 Gesundheitsdaten

Die Prüfzentren erfassen und übertragen zudem weitere Gesundheitsdaten der Teilnehmenden (Dauerdiagnosen, Grund des sozialen Rezepts).

6.1.3 Befragungsdaten der Hausärzt:innen

Nach Ende der Studie im jeweiligen Prüfzentrum, werden die beteiligten Hausärzt:innen per Online-Fragebogen befragt.

6.1.4 Rekrutierungsliste

Die für die Machbarkeitsendpunkte relevanten Angaben in den von den Prüfern ausgefüllten Rekrutierungslisten werden regelmäßig durch die Charité abgefragt.

6.1.5 Unerwünschte Ereignisse

Unerwünschte Ereignisse werden seitens der Hausärzt:innen innerhalb von sieben Tagen an das Studienteam gemeldet. Dafür stehen verschiedene sichere Übermittlungswege bereit, welche in der dazugehörigen SOP *SocPres_SOP Meldung, Bewertung und Umgang mit unerwünschten*

Ereignissen genauer beschrieben werden. Unerwünschte Ereignisse werden in einer zentralen Datenbank gespeichert (siehe Unerwünschte Ereignisse).

6.1.6 Befragungsdaten der Patient:innen

Mittels der von den Praxen in die personenidentfizierende Datenbank (PiD) eingetragenen Kontaktdaten werden die für die Studie rekrutierten Patient:innen von Study Nurses kontaktiert. Alle Befragungsdaten von Teilnehmenden werden durch ein computergestütztes Telefoninterview (CATI) nach Studieneinschluss der teilnehmenden Person (Baseline), nach 3 Monaten (3-Monats-Follow-Up) und nach 6 Monaten erhoben (6-Monats-Follow-Up) und direkt in das eCRF eingetragen.

6.1.7 Dokumentationen der Link Worker

Die Link Worker dokumentieren nach den Konsultationen mit den Teilnehmenden Gesprächsinhalte, vermittelte Angebote und Dauer der Konsultation in das eCRF in REDCap.

Dieser Prozess wird in der *SocPres_SOP Durchführung der Intervention* ausführlich beschrieben.

6.2 Daten mit und ohne Quelldaten

Folgende Daten beziehen sich auf Quelldaten: Persönliche Daten, Kontaktdaten und Dauerdiagnosen der Patient:innen (Quelldaten: Patient:innenakte/Praxisverwaltungssoftware) und unerwünschte Ereignisse (Quelldaten: Arztbriefe bei Behandlung in Notaufnahme oder Hospitalisierung, Dokumentation in der Praxisverwaltungssoftware). Für alle anderen Daten ist das eCRF das Quelldokument.

6.3 Archivierung und Zugang zu Quelldaten

Die Prüfer gewähren bis zu zehn Jahre nach Abschluss der Studien studienspezifische Zugang zu Quelldaten und Einwilligungserklärungen für Monitoring, Audits und Kontrollen der Ethikkommissionen.

6.4 Instrumente

Soziodemografische Daten: Angelehnt an die Europäische Gesundheitsumfrage (EHIS) und das Diversity Minimal Item Set (DiMiS) werden soziodemografische Daten (z.B. Alter, Geschlecht, schulische Ausbildung, Beruf, geschlechtliche Identität und Migrationshintergrund) erhoben (20). Darüber hinaus werden von den behandelnden Hausärzt:innen gegebenenfalls vorhandene Dauerdiagnosen der Teilnehmenden erfasst.

Gesundheitszustand (WHODAS 2.0): Der WHO Disability Assessment Schedule 2.0 ist ein generisches, von Patienten berichtetes Ergebnismaß für den Gesundheitszustand. Die Domänen von WHODAS 2.0 basieren auf dem konzeptionellen Rahmen der Internationalen Klassifikation der Funktionsfähigkeit, Behinderung und Gesundheit (ICF) der WHO, die seit 2001 der internationale Standard für die Beschreibung und Messung von Gesundheit und Behinderung ist. Alle sechs ICF-Bereiche (Kognition, Mobilität, Selbstversorgung, soziale Interaktion, Lebensaktivitäten und soziale Teilhabe) werden durch das WHODAS 2.0-Instrument abgedeckt. Es zeigt eine hohe interne Konsistenz, eine hohe Test-Retest-Reliabilität sowie eine hohe Validität im Vergleich zu anderen Instrumenten (21,22). Eine computergestützte adaptive Form mit 12 Items ist in deutscher Sprache verfügbar.

Psychisches Wohlbefinden (WHO-5): Der World Health Organization-Five Well-Being Index (WHO-5) ist ein kurzes, selbstberichtetes Maß für das aktuelle psychische Wohlbefinden. Die Skala hat eine ausreichende Validität als Ergebnismaß in klinischen Studien (23), und es liegen normative Daten für eine deutsche Version vor (24).

Allgemeine Lebenszufriedenheit (Kurzskala L-1 zur Erfassung der allgemeinen Lebenszufriedenheit): Die Kurzskala L-1 zur Erfassung der allgemeinen Lebenszufriedenheit besteht aus der im SOEP (25,26) etablierten Itemformulierung. Die Skala enthält nur ein Item mit folgendem Wortlaut: "Wie zufrieden sind Sie gegenwärtig insgesamt mit Ihrem Leben". Das Instrument wurde mit einer guten Reliabilität in deutscher Sprache validiert und es liegen normative Daten vor (25).

Einsamkeit (De Jong Gierveld Loneliness Scale): Die Kurzfassung der De Jong Gierveld Loneliness Scale umfasst 6 Items und umfasst zwei Subskalen zur emotionalen und sozialen Einsamkeit (27). Der Fragebogen ist valide und reliabel. Es existiert eine validierte deutsche Übersetzung.

Capability Well-being (ICECAP-A): Das ICEpop CAPability measure for Adults ist ein Maß für das Wohlbefinden der allgemeinen erwachsenen Bevölkerung (ab 18 Jahren) zur Verwendung bei der gesundheitsökonomischen Bewertung. Es existiert eine deutsche validierte Übersetzung (28).

Gesundheitsbezogene Lebensqualität (EQ-5D-5L): Der European Quality of Life 5 Dimensions 5 Level Version ist ein allgemeines Instrument, mit dem die Lebensqualität von Patient:innen unabhängig von ihrer Erkrankung bewertet werden kann. Er wurde umfassend, auch im deutschen Versorgungskontext, validiert (29).

Subjektiver Gesundheitszustand: Der Gesundheitszustand wird in Anlehnung an die Europäische Gesundheitsumfrage EHIS wie folgt erfragt: Wir würden Sie Ihren Gesundheitszustand im Allgemeinen beschreiben?

Zielbasierter Endpunkt (Goal based Outcome, GBO): GBOs sind personenzentrierte Endpunkte. Link Worker und Teilnehmer einigen sich auf ein Hauptziel, das erreicht werden soll. In den Follow Ups wird dann geklärt, ob dieses Ziel erreicht wurde oder wie weit die Person von der Erreichung entfernt ist. Der GBO wird nur in der Interventionsgruppe gemessen.

Inanspruchnahmeverhalten (relevante Abschnitte aus PECUNIA RUM bzw. EHIS-GeDA): Das PECUNIA Instrument zur Messung der Ressourcennutzung (PECUNIA RUM) misst die Ressourcennutzung in allen für die Kostenkalkulation aus gesellschaftlicher Sicht relevanten Sektoren der erwachsenen Bevölkerung: Gesundheits- und Sozialfürsorge, Bildung, (Straf-)Justiz, Produktivitätsverluste und informelle Pflege (30,31). In der vorliegenden Studie werden ausgewählte Items dieses Messinstruments verwendet, die sich auf die in der Studie untersuchten Themen zur Gesundheits- und Sozialfürsorge, sowie im Kontext von Produktivitätsverlusten beziehen, insbesondere Krankenhausaufenthalte und stationäre Betreuungseinrichtungen, Notfallversorgung, ambulante Gesundheitsleistungen und soziale Dienste, Medikation, informelle Pflege, Arbeitsunfähigkeit. Wenn möglich wird die für Deutschland kulturell adaptierte Version aus EHIS-GeDA genutzt.

7 Datenschutz

7.1 Datenschutzkonzept

Für die Studie wurde ein Datenschutzkonzept erstellt und durch das Clinical Trial Office der Charité Universitätsmedizin Berlin geprüft und beraten.

7.2 Ansprechpartner:innen für Betroffenenrechte

Die Kontaktdaten der Ansprechpartner:innen für Betroffenenrechte werden den an der Studie Teilnehmenden in der Studieninformation mitgeteilt.

8 Biometrie

8.1 Allgemein

In dieser Machbarkeitsstudie werden alle Ergebnisse explorativ interpretiert.

Die Einzelheiten der statistischen Analysen (einschließlich der Definition der Analysepopulationen, Subgruppen, Sensitivitätsanalysen usw.) werden in einem statistischen Analyseplan (SAP) vor Auswertung der Daten festgelegt. Es ist keine Zwischenanalyse geplant.

8.2 Fallzahlschätzung

Da es sich um eine explorative Machbarkeitsstudie ohne konfirmatorische Hypothesenprüfung handelt, wird keine formale Berechnung des Stichprobenumfangs durchgeführt. Aufgrund früherer Erfahrungen gehen wir davon aus, dass die Analyse von insgesamt 215 Teilnehmern (im Verhältnis 2:1: 143 im SP-Arm und 72 im TAU+-Arm) ausreicht, um die Machbarkeits- und Akzeptanzaspekte der Studie deskriptiv zu bestimmen und weitere Informationen für die Planung der anschließenden Studie zu erhalten.
Es wird mit einem Drop-Out von etwa 30 % gerechnet, so dass die Follow-up-Daten für sekundäre klinische Endpunkte von 150 Patienten (n = 100 im SP-Arm und n = 50 im TAU+-Arm) nach 6 Monaten zu Verfügung stehen.

8.3 Auswertungspopulationen

Die Analysen unter Verwendung des Full Analysis Sets (FAS) werden nach dem Intention-to-treat-Prinzip durchgeführt mit allen verfügbaren Daten (fehlende Daten werden nicht imputiert).

Die Per-Protocol-Population wird definiert als Patient:innen, die das Protokoll einhalten (Details werden im SAP definiert).

8.4 Analysen

Primäre Endpunkte: Der Anteil der Teilnehmer in der Interventionsgruppe, die mindestens einen Termin mit dem Link Worker wahrgenommen haben, und der Anteil der Teilnehmer, die die Studie vor der 6-monatigen Nachbeobachtung abbrechen (Abbruchrate), werden deskriptiv analysiert, mit Häufigkeiten und Prozenten, inkl. 95 % Konfidenzintervall pro Behandlungsgruppe. Der Vergleich der Abbrecherquote zwischen den Behandlungsgruppen erfolgt deskriptiv sowie mit Hilfe logistischer Regression, um die mögliche Einflussfaktoren zu bestimmen.

Sekundäre Endpunkte werden deskriptiv analysiert (Häufigkeiten/Prozente, Mittelwert/Standardabweichung oder Median/Interquartil je nach Skala und Verteilung, jeweils mit entsprechenden 95%-Konfidenzintervallen).

Vergleiche zwischen den Behandlungsgruppen werden mittels logistischer Regression (binäre Endpunkte) oder Kovarianzanalyse (kontinuierliche Endpunkte, adjustiert für den jeweiligen Baselinewert, sofern verfügbar) durchgeführt, wobei jeweils das Zentrum berücksichtigt wird.

Sicherheit: Unerwünschte Ereignisse werden deskriptiv analysiert (Häufigkeiten und Prozente pro Behandlungsgruppe).

8.5 Sensitivitätsanalysen

Relevante Endpunkte werden zusätzlich mit der Per-Protocol-Population analysiert.

Subgruppenanalysen werden für relevante Endpunkte durchgeführt. Dabei werden u. a. folgende Subgruppen verwendet:

- Geschlecht
- zugrunde liegende(s) psychosoziale(s) Problem(e)
- Altersgruppen.

Dies erfolgt ebenfalls deskriptiv sowie mit logistischer Regression oder Kovarianzanalyse mit zusätzlichem Faktor Interaktion (Behandlungsgruppe*Subgruppe) im Modell. Details der Definitionen der Subgruppen werden im SAP beschrieben.

Bei relevanten Unterschieden zwischen den Gruppen zu Baseline wird für diese Variablen zusätzlich adjustiert.

Clustering nach Zentrum/Arzt oder Link Worker wird mit verschiedenen Modellen (z. B. feste/zufällige Effekte) berücksichtigt.

9 Datenmanagement

Das Datenmanagement wird nach den Vorgaben des Qualitätsmanagementhandbuches des Instituts für Sozialmedizin, Epidemiologie und Gesundheitsökonomie durchgeführt. Die Daten werden nur für den jeweiligen Zweck gespeichert und verarbeitet. Die geltenden Datenschutzgesetze werden beachtet.

9.1 Erfassung und Speicherung der Daten

In der Einwilligungserklärung zur Teilnahme an der Studie gestattet die/der Studienteilnehmende, dass sie/er mit den im Rahmen dieser Erhebung erfolgenden Aufzeichnung, Verarbeitung und Speicherung ihrer /seiner persönlichen und medizinischen Daten einverstanden ist.

Die im Rahmen der Studie erhobenen Daten werden in der REDCap Online Datenbank (REDCap=Research Electronic Data Capture, Version 14.0 oder höher) erfasst und gespeichert. Das für die Erhebung der Daten verwendete REDCap läuft auf virtuellen Maschinen der Charité IT Infrastruktur. Der REDCap Webserver (erreichbar über https://redcap.charite.de) befindet sich in der sog. DMZ (demilitarized zone) hinter einer Firewall.

Personenidentifizierende Daten (PiD) werden durch das Praxispersonal in REDCap eingegeben. Die Forschungsdaten (FD) werden getrennt von den PiD in einem weiteren unabhängigen REDCap Projekt erhoben und gespeichert, es werden keine PiD an das Befragungsprojekt übermittelt.

Die Datenbanken sind nur für das Datenmanagement und autorisiertes Studienpersonal mit individualisiertem Login über eine 2-Faktor Authentifizierung zugänglich.

Die Studiendaten und Studienunterlagen werden für 10 Jahre nach Studienende archiviert und alle personenidentifizierenden Unterlagen und Daten anschließend gelöscht. Alle Forschungsdaten werden 10 Jahre nach Studienende anonymisiert.

9.2 Pseudonymisierung

Die Daten werden in REDCap unter Verwendung von Pseudonymen erfasst, diese bestehen aus fortlaufenden Nummern ohne Initialen oder Geburtsdaten der Teilnehmenden. Die PiD Datenbank ist die Re-Identifizierungsliste für die vom Studienpersonal in REDCap eingegebenen pseudonymisierten FD.

9.3 Rechte der Teilnehmenden (Widerruf, Datenlöschung, Recht auf Auskunft, Berichtigung)

Einer Weiterverarbeitung der Daten kann jederzeit von den Teilnehmenden widersprochen werden.

Die Teilnehmenden haben das Recht, die unmittelbare Löschung der Daten, die im Rahmen der Studie zu ihnen erhoben wurden, zu beantragen.

Für alle erhobenen Studiendaten besteht das Recht auf Auskunft. Die Teilnehmenden haben jederzeit Anspruch auf Auskunft und Berichtigung von zu Ihrer Person erhobenen Studiendaten.

10 Qualitative Prozessevaluation

Die Studie wird durch eine qualitative Prozessevaluation ergänzt, in der semi-strukturierte episodische Interviews mit an der Studie beteiligten Stakeholdern (Patient:innen, Hausärzt:innen, Verteter:innen lokaler Angebote und Angehörige des Studienteams) durchgeführt werden.

10.1 Ziele und Themen der qualitativen Prozessevaluation

In Tabelle 5 sind Ziele und Themen der qualitative Prozessevaluation dargestellt.

Tabelle 5: Ziele und Themen der qualitativen Prozessevaluation

Ziele Qualitative Prozessevaluation	Themen Qualitative Prozessevaluation	jeweils für:
Mit den qualitativen semi-strukturierten episodischen Interviews sollen die Perspektiven der beteiligten Akteure (Patient:innen, Hausärzt:innen, Link Worker und Vertreter:innen lokaler Angebote darstellen, um die Durchführbarkeit der Studie qualitativ zu bewerten, Erkenntnisse für zukünftige Studien und Versorgungsangebote zu gewinnen und Hinweise auf Kontaminationen zu ermitteln.	Akzeptanz der Studienprozeduren (u.a. Randomisierung) und der Intervention	Patient:innen
		Hausärzt:innen
		Link Worker
		Vertreter:innen lokaler Angebote
		Studienteam
	Praktikabilität der Studienprozeduren und Intervention	Patient:innen
		Hausärzt:innen
		Link Worker
		Vertreter:innen lokaler Angebote
		Studienteam
	Bedarf für und Nutzung der Intervention	Patient:innen
		Hausärzt:innen
		Link Worker

		Vertreter:innen lokaler Angebote
		Studienteam
	Potenziale für die Umsetzung der Intervention	Patient:innen (Interventionsarm)
		Hausärzt:innen
		Link Worker
		Vertreter:innen lokaler Angebote
		Studienteam
	Hürden für die Umsetzung der Intervention	Patient:innen (Interventionsarm)
		Hausärzt:innen
		Link Worker
		Vertreter:innen lokaler Angebote
		Studienteam
	Gefühl der Einbindung in die Nachbarschaft	Patient:innen

10.2 Rekrutierung für die qualitative Prozessevaluation

Für die an der Prozessevaluation teilnehmenden Patient:innen gelten weiterhin die Ein- und Ausschlusskritierien der Hauptstudie. Patient:innen können optional auf der Einwilligungserklärung der Hauptstudie ihre Einwilligung zur Kontaktaufnahme für die Rekrutierung der qualitativen Prozessevaluation geben. Ausgewählte Patient:innen werden im Zeitraum von bis zu 1 Jahr nach Abschluss der Intervention von den qualitativ Forschenden kontaktiert und bei Interesse aufgeklärt.

Teilnehmende Patient:innen erhalten eine unterschriebene Einwilligungserklärung und Studieninformation für die qualitative Prozessevaluation.

Ausgewählte Hausärzt:innen, Link Worker, Verteter:innen lokaler Angebote und Angehörige des Studienteams werden während und nach Abschluss der Studie über im Rahmen der Studie etablierte Kommunikationskanäle nach ihrem Interesse zur Teilnahme an der qualitativen Prozessevaluation befragt.

Einschlusskriterien:

- Teilnehmende sind einwilligungsfähige Erwachsene (18 Jahre und älter)

Ausschlusskriterien:

- Teilnehmende, die in Bezug auf die Gesundheit rechtlich betreut sind

Wir streben einen Einschluss von n=30-40 Teilnehmenden in die qualitative Prozessevaluation an, davon eine Hälfte Teilnehmende der beiden Studienarme und eine Hälfte andere Stakeholder der Studie. Das Sampling erfolgt schrittweise angelehnt an theoretisches Sampling bis eine theoretische Sättigung erreicht ist.

10.3 Datenerhebung, Datenspeicherung und Datenauswertung für die qualitative Prozessevaluation

Auf Basis der festgelegten Ziele und Themen der qualitativen Prozessevaluation werden Interviewleitfäden von Forschenden des Instituts für Allgemeinmedizin der Charité entwickelt. Neben den Fragen des Interviewleitfadens werden soziodemografische Angaben (Alter, Geschlecht, schulische Ausbildung und Beruf) erfragt. Die Forschenden führen die Interviews, nehmen sie auf und transkribieren sie. Das Interview findet in Präsenz am Campus Mitte der Charité, in der Hausarztpraxis, bei den Teilnehmenden zu Hause oder auf Wunsch der Teilnehmenden an einem neutralen Ort ihrer Wahl statt. Auf Wunsch ist auch ausnahmsweise eine telefonische Befragung möglich. Die Aufnahmen und transkribierten Interviews werden auf einem geschützten Charité-internen Laufwerk pseudonymisiert abgelegt. Die Interviews werden nach Thematischem Kodieren ausgewertet (32).

Im Rahmen des Mixed-Methods-Ansatzes werden die qualitativen Interviews parallel zu den quantitativen Endpunkten erfasst. Die Ergebnisse der Evaluation werden anhand der quantitativen und qualitativen Analysen u.a. von Akzeptanz, Praktikabilität und Bedarf/Nutzung trianguliert.

Die konkreten Handlungsabläufe der qualitativen Prozessevaluation sind in der *SocPres_SOP Qualitative Prozessevaluation* ausführlich beschrieben.

11 Begleitende gesundheitsökonomische Evaluation

Die Machbarkeitsstudie wird durch eine gesundheitsökonomische Evaluation begleitet, welche die quantitativ erhobenen Daten der Studie nutzt. Ziel dieser ist zum einen, die Akzeptanz der Erhebungsinstrumente zum Wohlbefinden (ICECAP-A), zur Lebensqualität (EQ-5D-5L) sowie zur Ressourceninanspruchnahme (PECUNIA RUM/EHIS-GeDA) zu überprüfen. Darüber hinaus wird aus der Dokumentation der Link Worker extrahiert, welche Angebote Patient:innen vermittelt wurden und wie die Patient:innen – Link Worker Interaktion verlief. Ebenfalls werden die erhobenen Leistungsinanspruchnahmen im Gruppenvergleich ausgewertet, um Leistungsbereiche mit einem signifikantem Gruppenunterschied zu identifizieren. Die explorativen Auswertungen verwenden das 5% und 10% Signifikanzniveau. Die Erkenntnisse werden verwendet, um die Erhebungsinstrumente für eine Folgestudie zu überarbeiten, d.h. Aspekte im PECUNIA RUM Instrument, welche im Anwendungskontext „Soziales Rezept“ weniger bedeutend sind, zu streichen und das Spektrum der außerhalb des Gesundheitswesens vermittelten Angebote für eine standardisierte Dokumentation und Ermittlung von Standardkosten aufzunehmen.

Ergänzend zu diesen Forschungsprozess bezogenen Ergebnissen werden die erhobenen Daten zur Leistungsinanspruchnahme sowie zur Lebensqualität und zum Wohlbefinden inhaltlich ausgewertet. Für die monetäre Bewertung der Ressourcennutzung werden die Standardkosten für alle (privat erworbenen oder verschriebenen) Dienstleistungen und Güter in Euro berechnet. Die Kosteneffektivität der Intervention wird über den Net Benefit Ansatz bestimmt. Die statistische Auswertung der Daten wird je nach Verteilung der Daten mittels parametrischer oder nicht-parametrischer Verfahren erfolgen. Abschließend werden aus gesundheitsökonomischer Sicht relevante Subgruppen identifiziert und Aussagen zur erreichten Power der Ergebnisse getätigt, um die Fallzahlschätzungen für eine potentielle Folgestudie zu unterstützen.

12 Qualitätsmanagement und -sicherung

Die Studie erfolgt nach den Richtlinien der ICH-GCP. Am 17.05.2024 erfolgte die Pflichtberatung durch die QA-Unit mit Bescheinigung vom 15.07.2024. Die in diesem Zusammenhang nach den Vorgaben der QA-Unit der Charité notwendige Sponsorvollmacht wurde dem Studienleiter am 22.07.2024 durch den Dekan der Charité - Universitätsmedizin Prof. Dr. Joachim Spranger erteilt.

12.1 Standard Operating Procedures (SOPs)

Der Studie liegt ein Qualitätsmanagement mit SOPs entsprechend der Studiendurchführung nach Berufsrecht zugrunde, welches die nötigen Prozesse zur Wahrnehmung der Aufgaben der Studienleitung bzw. Sponsoraufgaben und insbesondere zur Sponsor Oversight vorgibt.

12.2 Monitoring

Das Monitoring (Studienüberwachung) soll nach ICH-GCP sicherstellen, dass

a) „die Rechte und das Wohlergehen der Versuchspersonen geschützt sind.

b) die gemeldeten Studiendaten genau, vollständig und anhand von Quelldokumenten überprüfbar sind.

c) die Durchführung der Studie in Übereinstimmung mit dem aktuell genehmigten Protokoll/den Änderungen, der guten klinischen Praxis und den geltenden behördlichen Vorschriften erfolgt.“ (ICH-GCP, 5.18.1)

Im Rahmen der Risikobewertung wurden das Risiko für das Auftreten unerwünschter Ereignisse und Risiken für Rechte und Wohlergehen der Versuchspersonen als sehr niedrig eingeschätzt. Aufgrund der geringen Vorerfahrung der Prüfzentren wurden Risiken für Fehler bei der Studiendokumentation und Abweichungen vom Protokoll durch die Prüfzentren jedoch als durchaus möglich eingestuft. Nach ADAMON entschieden wir uns deshalb für ein risikoadaptiertes Vorgehen in der Stufe K2 (33).

Zusammengefasst besteht das Monitoring aus zentralem Monitoring (Central), sowie aus Monitoring vor Ort (On Site). Außerdem wird das Monitoring durch die Studienkoordination, eine unabhängige spezielle Monitorin und ein Co-Monitoring durch das Clinical Trials Office (CTO) der Charité durchgeführt. Das genaue Vorgehen ist im SocPres_Monitoringplan beschrieben.

13 Sicherheit

13.1 Unerwünschte Ereignisse

In der Literatur zu Social Prescribing sind keine unerwünschten Ereignisse im Zusammenhang mit der Intervention beschrieben. Zum Nachweis der Unbedenklichkeit und Sicherheit wird jedoch im Einklang mit ICH-GCP ein umfassendes Überwachungsverfahren etabliert, welches aus kurzfristigen Meldungen zu Einzelfallberichten, periodischen Berichten, der Sicherheitsüberwachung durch ein unabhängiges Gremium (DSMB, s.u.) und einer umfassenden Sicherheitsanalyse der Studie besteht.

13.1.1 Definition unerwünschter Ereignisse

Da es sich um eine sonstige Studie nach §15 Berufsordnung für Ärzte mit einer komplexen Intervention ohne Arzneimittel oder Medizinprodukt handelt, wird von einer Klassifikation der unerwünschten Ereignisse nach Common Terminology Criteria for Adverse Events (CTCAE) v5.0 abgesehen. Stattdessen werden für die Studie folgende unerwünschte Ereignisse vordefiniert:

1) Tod (jede Ursache)

2) Notaufnahmebesuch (jede Ursache)

Aufsuchen einer Notaufnahme. Die Meldung soll unabhängig davon erfolgen, ob die Person im Anschluss stationär aufgenommen wird oder nicht.

3) Ungeplante Hospitalisierung (jede Ursache)

Eine ungeplante Hospitalisierung ist dadurch charakterisiert, dass eine Krankenhauseinweisung ohne vorherige Terminvereinbarung erfolgt.

4) Suizidversuch/Suizidalität

Der Suizidversuch wird als ein selbst ausgeführtes, schädigendes und mit der zu Sterben beabsichtigtes Verhalten, welches aber nicht zum Tod führte (ICD-11: MB23.R, ICPC-3: PD14), definiert.

Suizidalität wird in diesem Kontext als Gedanken, Ideen oder Grübeleien über die Möglichkeit, das eigene Leben zu beenden, die von der Überlegung, dass man besser tot wäre, bis hin zur Formulierung ausgeklügelter Pläne reichen, verstanden (ICD-11: MB26.A, ICPC-3: PS05).

5) Sonstige relevante unerwünschte Ereignisse

Damit Prüfer:innen die Möglichkeit bekommen weitere aus Ihrer Sicht als relevant eingestufte unerwünschte Ereignisse zu melden, gibt es eine gesonderte Kategorie.

13.1.2 Meldung unerwünschter Ereignisse

Die Meldung unerwünschter Ereignisse erfolgt mittels des Formulars *SocPres_MeldeformularEreignisse* über sichere Kommunikationswege durch die Prüfer:innen an die Studienleitung innerhalb von 7 Tagen nach Bekanntwerden. Das genaue Vorgehen ist in der *SOP SocPres_SOP: Meldung von, Bewertung von und Umgang mit unerwünschten Ereignissen* festgelegt.

13.1.3 Zentrale Dokumentation unerwünschter Ereignisse

Alle unerwünschten Ereignisse werden durch die Studienleitung zentral in einer Datenbank erfasst: *SocPres_LineListing* und bis zur abschließenden Bewertung nachverfolgt.

13.1.4 Zweitbewertung unerwünschter Ereignisse

Die Zweitbewertung unerwünschter Ereignisse erfolgt mittels des Formulars *SocPres_BewertungsbogenEreignisse* durch die Studienleitung innerhalb von 7 Tagen nach Übermittlung durch die Prüfer:innen. Das Vorgehen ist in der SOP *SocPres_SOP Meldung von, Bewertung von und Umgang mit unerwünschten Ereignissen* festgelegt.

13.2 Berichterstattung

Nach Rekrutierung von jeweils 50 Patient:innen oder spätestens alle drei Monate wird ein Sicherheitsbericht mit den gemeldeten unerwünschten Ereignisse erstellt, der dem DSMB vorgelegt wird.

13.3 Data Safety Monitoring Board (DSMB)

Die Ziele des DSMB sind

- die Wahrung der Interessen der Studienteilnehmenden,
- die Bewertung der Sicherheit und Wirksamkeit der Intervention während der Studie und
- die Überwachung der Durchführung der Studie.

Das DSMB erhält und überprüft Informationen über den Fortschritt und die anfallenden Daten der Studie.

Dies beinhaltet:

1. die Bewertung der Datenqualität, einschließlich der Vollständigkeit,
2. die Überwachung der Rekrutierungszahlen und des Loss to follow up,
3. die Überwachung der Einhaltung des Prüfplans durch Teilnehmende und Prüfer:innen,
4. die Überwachung von unerwünschten Ereignissen,
5. die Überwachung der Einhaltung früherer DSMB-Empfehlungen,
6. die ethischen Implikationen aller Empfehlungen des DSMB zu berücksichtigen.

Das DSMB soll entscheiden, ob empfohlen wird, die Rekrutierung von Teilnehmern für die Studie fortzusetzen oder die Rekrutierung entweder für alle oder für einige Behandlungsgruppen und/oder

einige Untergruppen von Teilnehmenden gestoppt werden soll. Die Empfehlungen des DSMB sind beratend und nicht ausführend.

Die Zusammensetzung des DSMB, die Arbeitsweise (inkl. Entscheidungsfindung) und die Berichterstattung sind in dem *SocPres_DSMB-Charter* festgelegt.

14 Ethische Erwägungen

Die Intervention gilt als risikoarm für die Patient:innen, da in früheren Studien keine unerwünschten Ereignisse gemeldet wurden, auch wenn diese möglicherweise nicht adäquat erfasst wurden.

14.1 Individueller Nutzen

Der potenzielle Nutzen besteht in einer Verbesserung der individuellen Gesundheit und des Wohlbefindens.

Teilnehmende in der Interventionsgruppe profitieren durch die Möglichkeit mehrere Termine mit einem Link Worker in Anspruch zu nehmen und über eine personenzentrierte Interaktion eigene Bedürfnisse herauszufinden, zu artikulieren und Lösungsansätze in die Umsetzung zu bringen. Durch die Unterstützung sollen Hürden reduziert und die Wahrscheinlichkeit erhöht werden, dass die Teilnehmenden Angebote in Anspruch nehmen können. Es ist zu erwarten, dass die Kommunikation mit dem Link Worker selbst einen individuellen therapeutischen Nutzen hat (Motivational Interviewing), jedoch insbesondere auch Vermittlung an Angebote vor Ort (z.B. durch die Zunahme sozialer Kontakte im Rahmen einer Teilnahme beim Chor zur Verringerung von Einsamkeit oder die Inanspruchnahme von Schuldnerberatung bei finanziellen Problemen).

Teilnehmende in der Kontrollgruppe profitieren durch die Möglichkeit ihre medizinischen und sozialen Bedürfnisse kommunizieren zu können. Außer der Informationsweitergabe zu Angeboten vor Ort ("Signposting"), damit die Teilnehmenden sich selbst um Beratung oder Nutzung von Angeboten kümmern können, ist für diese Gruppe jedoch kein individueller Nutzen zusätzlich zur regulären Versorgung zu erwarten.

14.2 Gruppennutzen

Langfristig dienen die Ergebnisse der Studie der Verbesserung der sozialen und gesundheitlichen Versorgung von Menschen mit nicht-medizinischen gesundheitsrelevanten Bedürfnissen. Die

qualitativ hochwertigen evidenzbasierten Ergebnisse sollen dazu dienen politische Entscheidungstragende über mögliche Maßnahmen zur Reduktion gesundheitlicher Ungleichheiten und der Verbesserung des Gesundheitsstatus, des Wohlbefindens und weiterer patient:innenrelevanter Endpunkte zu informieren. Durch die Ergebnisse wird ggf. eine Implementierung des Sozialen Rezepts als integriertes Versorgungsmodell in die reguläre Versorgung ermöglicht. Dadurch könnte das Konzept allen Menschen mit nicht-medizinischen gesundheitsrelevanten Problemen, die einen Zugang zu hausärztlicher Versorgung haben, zugutekommen. Potenziell ist auch eine Ausweitung außerhalb der hausärztlichen Versorgung (z.B. durch Apotheken, Notaufnahmen, aber auch städtische Angebote, wie die Arbeitsagentur, das Bürgeramt oder die Bibliothek) möglich. Dies wird in anderen Ländern bereits praktiziert.

Außerdem besteht das Potenzial, dass die Intervention eine geringere Inanspruchnahme der Gesundheitsversorgung, insbesondere der hausärztlichen Versorgung, aber auch der Inanspruchnahme von Notaufnahmen zur Folge hat. Dies hat das Potenzial das Gesundheitssystem zu entlasten.

14.3 Nutzen für teilnehmende Prüfzentren

Die teilnehmenden Prüfzentren erhalten pro rekrutierte:n Patient:in eine Aufwandsentschädigung von 50 EUR.

14.4 Schaden/Risiko

Für Studienteilnehmer:innen in Kontroll- und Interventionsgruppe bedeutet die Teilnahme ein Zeitaufwand, u.a. für die Befragungen.

Für Teilnehmer:innen in der Interventionsgruppe wäre denkbar, dass die Gespräche mit dem Link Worker emotional aufwühlend sein könnten und daher z.B. zu einer Dekompensation führen könnten. Jedoch wurden solche unterwünschten Wirkungen in den bisherigen Studien und in der flächendeckenden Implementation in Großbritannien nicht berichtet. Im Gegenteil werden die Gespräche eher entlastend gewertet.

14.5 Zusammenfassende Bewertung

Zusammenfassend kommen wir zu der Bewertung, dass der Nutzen das Risiko überwiegt.

14.6 Zuständige Ethikkommissionen

Das zustimmende Erstvotum erfolgt bei der federführenden Ethikkommission der Charité Universitätsmedizin Berlin. Das zusätzliche Votum gemäß den rechtlichen Vorgaben für die beiden Prüfzentren in Brandenburg erfolgt bei der Ethikkommission der Landesärztekammer Brandenburg als beteiligte Ethikkommission.

15 Versicherung der Studienteilnehmenden

In Rücksprache mit dem Geschäftsbereich Recht der Charité – Universitätsmedizin Berlin besteht für die Studie Versicherungsschutz im Rahmen der bestehenden Betriebs-Haftpflichtversicherung der Charité der HDI-Gerling AG (Versicherungsschein-Nr.: 56-99629701016) (*Schreiben des Versicherungsreferats vom 19.07.2024*).

16 Publikationsleitlinie

Folgende Hauptpublikationen sind geplant:

- Publikation zur Machbarkeit der Intervention mit den primären Endpunkten
- Publikation zur klinischen Wirksamkeit anhand klinischer Endpunkte
- Publikation zu Sicherheit und unerwünschter Ereignisse
- Publikation zur Prozessevaluation

Der Studienleiter und stellvertretende Studienleiter sind bei allen Publikationen als Autoren beteiligt. Die Biometrikerin ist bei allen Publikationen mit quantitativen Daten beteiligt. Die Hauptprüfer werden an allen Publikationen mit quantitativen Patientendaten als Autoren beteiligt, falls Sie die Kriterien der „Leitlinien zur Sicherung guter wissenschaftlicher Praxis“ der DFG erfüllen.

Es wird ein Publikationsplan erstellt und regelmäßig aktualisiert, der die Regelungen zu Autorenschaften enthält. Die Regelungen orientieren sich an den „Leitlinien zur Sicherung guter wissenschaftlicher Praxis“ der DFG und den Empfehlungen des International Committee of Medical Journal Editors.

17 Referenzen

1. Zimmermann T, Mews C, Kloppe T, Tetzlaff B, Hadwiger M, von dem Knesebeck O, et al. [Social problems in primary health care - prevalence, responses, course of action, and the need for support from a general practitioners’ point of view]. Z Evidenz Fortbild Qual Im Gesundheitswesen. 2018 Apr;131–132:81–9.

2. Holt-Lunstad J, Smith TB, Layton JB. Social Relationships and Mortality Risk: A Meta-analytic Review. PLOS Med. 2010 Jul 27;7(7):e1000316.

3. Valtorta NK, Kanaan M, Gilbody S, Ronzi S, Hanratty B. Loneliness and social isolation as risk factors for coronary heart disease and stroke: systematic review and meta-analysis of longitudinal observational studies. Heart. 2016;102(13):1009–16.

4. Lorant V, Croux C, Weich S, Deliège D, Mackenbach J, Ansseau M. Depression and socio-economic risk factors: 7-year longitudinal population study. Br J Psychiatry. 2007;190:293–8.

5. North FM, Syme SL, Feeney A, Shipley M, Marmot M. Psychosocial work environment and sickness absence among British civil servants: the Whitehall II study. Am J Public Health. 1996;86(3):332–40.

6. Shiels C, Gabbay MB, Ford FM. Patient factors associated with duration of certified sickness absence and transition to long-term incapacity. Br J Gen Pract. 2004;54(499):86–91.

7. Herrmann WJ, Haarmann A, Bærheim A. Arbeitsunfähigkeitsregelungen als Faktor für Inanspruchnahme ärztlicher Versorgung in Deutschland. Z Für Evidenz Fortbild Qual Im Gesundheitswesen. 2015 Jan 1;109(8):552–9.

8. ICPC-3 Consortium of the World Organization of Family Doctors. Chapter ZC Social problems influencing health status. In: van Boven K, Ten Napel H, editors. ICPC-3 International Classification of Primary Care: User Manual and Classification. CRC Press; 2021.

9. Herrmann WJ, Oeser P, Buspavanich P, Lech S, Berger M, Gellert P. Loneliness and depressive symptoms differ by sexual orientation and gender identity during physical distancing measures in response to COVID-19 pandemic in Germany. Appl Psychol Health Well-Being. 2023;15(1):80–96.

10. Marmot M. Achieving health equity: from root causes to fair outcomes. The Lancet. 2007 Sep 29;370(9593):1153–63.

11. Zantinge EM, Verhaak PFM, Kerssens JJ, Bensing JM. The workload of GPs: consultations of patients with psychological and somatic problems compared. Br J Gen Pract. 2005;55(517):609–14.

12. Popay J, Kowarzik U, Mallinson S, Mackian S, Barker J. Social problems, primary care and pathways to help and support: addressing health inequalities at the individual level. Part II: lay perspectives. J Epidemiol Community Health. 2007;61(11):972–7.

13. Morse DF, Sandhu S, Mulligan K, Tierney S, Polley M, Chiva Giurca B, et al. Global developments in social prescribing. BMJ Glob Health. 2022 May;7(5):e008524.

14. Muhl C, Mulligan K, Bayoumi I, Ashcroft R, Godfrey C. Establishing internationally accepted conceptual and operational definitions of social prescribing through expert consensus: a Delphi study. BMJ Open. 2023 Jul 1;13(7):e070184.

15. Islam MM. Social Prescribing—An Effort to Apply a Common Knowledge: Impelling Forces and Challenges. Front Public Health. 2020;8:515469.

16. Napierala H, Krüger K, Kuschick D, Heintze C, Herrmann WJ, Holzinger F. Social Prescribing: Systematic Review of the Effectiveness of Psychosocial Community Referral Interventions in Primary Care. Int J Integr Care. 2022 Aug 19;22(3):11.

17. Zwarenstein M. ‘Pragmatic’ and ‘explanatory’ attitudes to randomised trials. J R Soc Med. 2017 May 1;110(5):208–18.

18. NHS England. Workforce development framework: social prescribing link workers [Internet]. [cited 2023 Feb 9]. Available from: https://www.england.nhs.uk/long-read/workforce-development-framework-social-prescribing-link-workers/

19. Gesundheit Österreich GmbH. Informationen und Unterstützungstools zu Social Prescribing [Internet]. [cited 2024 Aug 25]. Available from: https://goeg.at/SocialPrescribing_Dokumente

20. Stadler G, Chesaniuk M, Haering S, Roseman J, Straßburger VM, Martina S, et al. Diversified innovations in the health sciences: Proposal for a Diversity Minimal Item Set (DiMIS). Sustain Chem Pharm. 2023 Jun 1;33:101072.

21. Federici S, Bracalenti M, Meloni F, Luciano JV. World Health Organization disability assessment schedule 2.0: An international systematic review. Disabil Rehabil. 2017 Nov;39(23):2347–80.

22. Ustün TB, Chatterji S, Kostanjsek N, Rehm J, Kennedy C, Epping-Jordan J, et al. Developing the World Health Organization Disability Assessment Schedule 2.0. Bull World Health Organ. 2010 Nov 1;88(11):815–23.

23. Topp CW, Østergaard SD, Søndergaard S, Bech P. The WHO-5 Well-Being Index: A Systematic Review of the Literature. Psychother Psychosom. 2015;84(3):167–76.

24. Brähler E, Mühlan H, Albani C, Schmidt S. Teststatistische Prüfung und Normierung der deutschen Versionen des EUROHIS-QOL Lebensqualität-Index und des WHO-5 Wohlbefindens-Index. Diagnostica. 2007 Apr;53(2):83–96.

25. Richter D, Metzing M, Weinhardt M, Schupp J. SOEP scales manual [Internet]. Berlin: Deutsches Institut für Wirtschaftsforschung (DIW); 2013 [cited 2023 Feb 9]. (SOEP Survey Papers; vol. 138). Available from: https://www.econstor.eu/bitstream/10419/85279/1/770557678.pdf

26. GESIS-Leibniz-Institut Für Sozialwissenschaften. ALLBUS/GGSS 1980-2012 (Kumulierte Allgemeine Bevölkerungsumfrage der Sozialwissenschaften/ Cumulated German General

Social Survey 1980-2012) [Internet]. GESIS Data Archive; 2014 [cited 2023 Feb 6]. Available from: https://search.gesis.org/research_data/ZA4578?doi=10.4232/1.11898

27. De Jong Gierveld J, Van Tilburg T. The De Jong Gierveld short scales for emotional and social loneliness: tested on data from 7 countries in the UN generations and gender surveys. Eur J Ageing. 2010 Jun;7(2):121–30.

28. Polley M, Richards R. A guide to selecting patient reported outcome measures (PROMs) for social prescribing [Internet]. University of Westminster, London; [cited 2022 Sep 28]. Available from: https://www.london.gov.uk/sites/default/files/a_guide_to_selecting_outcomes_measures_in_social_prescribing_final.pdf

29. Richmond Group. Social prescribing: Options for outcome measurement A Summary [Internet]. [cited 2022 Sep 28]. Available from: https://richmondgroupofcharities.org.uk/sites/default/files/dtrt_summary_of_learning_about_outcomes_measurement_for_social_prescribing.pdf

30. Jolliffe R, Seers H, Jackson S, Caro E, Weeks L, Polley MJ. The Responsiveness, Content Validity, and Convergent Validity of the Measure Yourself Concerns and Wellbeing (MYCaW) Patient-Reported Outcome Measure. Integr Cancer Ther. 2015 Jan 1;14(1):26–34.

31. Law D, Jacob J. Goals and goal based outcomes (GBOs) Some Useful Information [Internet]. Third Edition. CAMHS Press; 2015 [cited 2022 Sep 28]. Available from: https://www.corc.uk.net/media/1219/goalsandgbos-thirdedition.pdf

32. Wright B, Marshall D, Adamson J, Ainsworth H, Ali S, Allgar V, et al. Social Stories^{TM} to alleviate challenging behaviour and social difficulties exhibited by children with autism spectrum disorder in mainstream schools: design of a manualised training toolkit and feasibility study for a cluster randomised controlled trial with nested qualitative and cost-effectiveness components. NIHR Journals Library; 2016.

33. Linton MJ, Mitchell PM, Al-Janabi H, Schlander M, Richardson J, Iezzi A, et al. Comparing the German Translation of the ICECAP-A Capability Wellbeing Measure to the Original English Version: Psychometric Properties across Healthy Samples and Seven Health Condition Groups. Appl Res Qual Life. 2020 Jul 1;15(3):651–73.

34. Janssen MF, Pickard AS, Golicki D, Gudex C, Niewada M, Scalone L, et al. Measurement properties of the EQ-5D-5L compared to the EQ-5D-3L across eight patient groups: a multi-country study. Qual Life Res Int J Qual Life Asp Treat Care Rehabil. 2013 Sep;22(7):1717–27.

35. Brosteanu O, Schwarz G, Houben P, Paulus U, Strenge-Hesse A, Zettelmeyer U, et al. Risk-adapted monitoring is not inferior to extensive on-site monitoring: Results of the ADAMON cluster-randomised study. Clin Trials Lond Engl. 2017 Dec;14(6):584–96.

18 Abkürzungsverzeichnis

CATI	Computergestützte Telefoninterviews
eCRF	elektronische Case Report Form
DSMB	Data Safety and Monitoring Board
EHIS	Europäisches Gesundheitsinterview
FD	Forschungsdatenbank
GCP	Good Clinical Practice
GeDA	Gesundheit Deutschland Aktuell
PiD	Personenidentifizierende Datenbank
PROM	Patient Reported Outcomes
REDCap	Research Electronic Data Capture
SAP	Statistischer Analyseplan
SOP	Standard Operating Procedure
SP	Social Prescribing
TAU+	Treatment-As-Usual plus Broschüre mit Informationen über lokale Angebote für nicht-klinische Unterstützungsangebote

19 Abbildungsverzeichnis

Abbildung 1: Studiendesign 20
Abbildung 2: CONSORT-Flussdiagramm 28

20 Tabellenverzeichnis

Tabelle 1: Synopse 9
Tabelle 2: Projektziele und Endpunkte 17
Tabelle 3: Intervention nach dem TIDieR Framework 22
Tabelle 4: Visitenübersicht 25
Tabelle 5: Ziele und Themen der qualitativen Prozessevaluation 38

21 Unterschriften

Datum, Ort	Prof. Dr. Wolfram Herrmann, Studienleitung
Datum, Ort	PD Dr. Stephanie Roll, Biostatistikerin
