# Supplementary material for: Social prescribing to improve health and well-being of patients presenting with non-medical health related social needs in primary care: Study protocol of a multi-center randomized controlled pragmatic feasibility trial
Source: PLoS One. 2025 May 23;20(5):e0322372. doi: 10.1371/journal.pone.0322372 (PMC12101629; doi:10.1371/journal.pone.0322372)
Supplement: S2 File — (PDF) [file pone.0322372.s002.pdf]

Prüfplan

Das „Soziale Rezept“ zur Verbesserung von Gesundheit und Wohlbefinden von Patient:innen, die sich mit nicht-medizinischen gesundheitsbezogenen sozialen Problemen in der hausärztlichen Versorgung vorstellen: eine multizentrische, randomisiert kontrollierte, pragmatische Machbarkeitsstudie

„Soziales Rezept“

V1.2 vom 12.02.2025

Version without logo and automated translation into English

Table of contents

1. General information about the study 5
   1.1 Project title, version number, version date, version history 5
   1.2 Responsibilities 6
   1.3 Test centers and principal investigators 7
   1.4 Financing 8
   1.5 Registration in a publicly accessible study register 8
   1.6 Study Synopsis 9

2 Background 14

3 Project goals and endpoints 15

4 Study design 19
   4.1 Study design 19
   4.2 Justification of the study design 20
   4.3 Intervention 20
   4.4 Control 22
   4.5 Measures to reduce bias 22
      4.5.1 Randomization and allocation 22
      4.5.2 Blinding 23
   4.6 Study participants and centers 23
      4.6.1 Inclusion and exclusion criteria for trial sites 23
      4.6.2 Inclusion and exclusion criteria for patients 23
   4.7 Patient pathway and visits 24
      4.7.1 t_{-1} : Consultation in practice 27
      4.7.2 t_0 : Baseline data collection and randomization 28
      4.7.3 t_1 Follow-up survey after three months 29
      4.7.4 t_2 Follow-up survey after six months 29
   4.8 Medical care 29

5 Termination Criteria 29
   5.1 Termination criteria for individual study participants 29
   5.2 Termination criteria for parts of the study or the entire study 29

6 Data collection 30
   6.1 Data sources 30
      6.1.1 Personally Identifiable Data 30
      6.1.2 Health data 30
      6.1.3 Survey data from general practitioners 30
      6.1.4 Recruitment list 30

6.1.5 Adverse events ... 30
6.1.6 Patient survey data ... 30
6.1.7 Documentation of the Link Worker ... 31
6.2 Data with and without source data ... 31
6.3 Archiving and access to source data ... 31
6.4 Instruments ... 31
7 Data Protection ... 33
7.1 Data protection concept ... 33
7.2 Contact persons for data subject rights ... 33
8 Biometrics ... 33
8.1 General ... 33
8.2 Sample size estimate ... 33
8.3 Evaluation populations ... 34
8.4 Analyses ... 34
8.5 Sensitivity analyses ... 34
9 Data Management ... 35
9.1 Collection and storage of data ... 35
9.2 Pseudonymization ... 36
9.3 Rights of participants (revocation, data deletion, right to information, correction) .. 36
10 Qualitative Process Evaluation ... 36
10.1 Objectives and topics of qualitative process evaluation ... 36
10.2 Recruitment for qualitative process evaluation ... 38
10.3 Data collection, data storage and data analysis for qualitative process evaluation 38
11 Accompanying health economic evaluation ... 39
12 Quality management and assurance ... 40
12.1 Standard Operating Procedures (SOPs) ... 40
12.2 Monitoring ... 40
13 Security ... 41
13.1 Adverse events ... 41
13.1.1 Definition of adverse events ... 41
13.1.2 Reporting of adverse events ... 42
13.1.3 Central documentation of adverse events ... 42
13.1.4 Secondary assessment of adverse events ... 42
13.2 Reporting ... 42
13.3 Data Safety Monitoring Board (DSMB) ... 42
14 Ethical Considerations ... 43
14.1 Individual Benefit ... 43

14.2 Group Benefits 44
14.3 Benefits for participating trial sites 44
14.4 Damage/Risk 44
14.5 Summary assessment 44
14.6 Competent ethics committees 44
15 Insurance of study participants 45
16 Publication Guideline 45
17 References 46
18 List of Abbreviations 49
19 List of Figures 50
20 List of Tables 51
21 signatures 52

1. General information about the study

1.1 project title, version number, version date, version history

title

The ‘Social Prescription’ to improve health and wellbeing of patients presenting to primary care with non-medical health-related social problems: a multicentre, randomised controlled, pragmatic feasibility study

short title

"Social prescribing"

version number

1.2

version date

February 12, 2025

version	Reason for change or short description (section) of the changes
V1.0	initial creation
V1.1	Satisfaction measurement also after three months and both arms (3); Goal-based outcome also by link worker (4.3); More detailed classification of social problems (4.6.2); Adaptation of response categories of subjective health status (6.4)
1.2	Two additional study centers added: Neukölln GP practice (main examiner Jihan Saeed), Weichselstrasse practice (main examiner: Dr. med. Anja Zitscher). Exclusion criterion “a person living in the same household is already included in the study” added. Exclusion criterion “legally supervised” specified as “ legally supervised with regard to health”.

1.2 responsibilities

Sponsor
Charité – Universitätsmedizin Berlin
Charitéplatz 1
10117 Berlin

Study Director/ Sponsor Representative Prof. Dr. med. Wolfram Herrmann
Institute for General Practice and Family Medicine
Charité – Universitätsmedizin Berlin
Charitéplatz 1
10117 Berlin

Deputy Head of Study Dr. med. Hendrik Napierala M.Sc.
Institute for General Practice and Family Medicine
Charité – Universitätsmedizin Berlin
Charitéplatz 1
10117 Berlin

Responsible biometrician PD Dr. Stephanie Roll
Institute for Social Medicine, Epidemiology and Health Economics
Charité – Universitätsmedizin Berlin
Charitéplatz 1
10117 Berlin

1.3 test centers and principal investigators

Gemeinschaftspraxis für Allgemeinmedizin
Dr. med. Kathrin Hecker
Landsberger Allee 44
10249 Berlin

Ärzte am Hermannplatz
Gerd Michels
Kottbusser Damm 72
10967 Berlin

MVZ Praxis Korok
Rheinsteinstr. 1
10318 Berlin

Hausarztpraxis Lichtenberg
Iris Boehmer
Möllendorffstr. 45
10367 Berlin

Gesundheitszentrum Haus Nazareth
Dr. med. Burghard Storm
Wrangelstr. 6/7
12165 Berlin

Hausarztpraxis Dashti
Dr. med. Hiwa Dashti
Am Paschenberg 30
16225 Eberswalde

Hausarzt Dr. Zerbaum & Kollegen MVZ
Dr. med. Mario Zerbaum
Petersilienstraße 12
14776 Brandenburg

Hausarztpraxis Neukölln
Jihan Saeed
Hermannstraße 52
12049 Berlin

Praxis Weichselstraße
Dr. med. Anja Zitscher
Weichselstr. 59
12045 Berlin

1.4 financing

The study is funded by the German Research Foundation in the "Clinical Studies" program as

feasibility study funded.

Application number: HE 6399/3-1

Project number: 530364906

German Research Foundation (DFG)

Kennedyallee 40

53175 Bonn

1.5 Registration in a publicly accessible study register

German Clinical Trials Register (DRKS)

DRKS-ID: DRKS00034654

1.6 study synopsis

Table 1: Synopsis

degree title (German)	The 'Social Prescription' to improve health and wellbeing of patients presenting to primary care with non-medical health-related social problems: a multicentre, randomised controlled, pragmatic feasibility study
Title of trial (English)	Social prescribing to improve health and well-being of patients presenting with non-medical health related social needs in primary care: a multi- center randomized controlled pragmatic feasibility trial
indication	Non-medical health-related Problems (International Classification of Primary Care 3rd Edition, ICPC-3: ZC01-ZC99 " Social problems ")
Goal	The main objective of this feasibility study is to assess the feasibility of conducting a randomized controlled trial on the "social prescription" in Germany and to provide information and data necessary for planning a confirmatory study (e.g., recruitment and study procedures, feasibility of individual randomization versus cluster randomization, suitability of endpoints, sample size calculation).
study design	Multicenter, two-arm, randomized (2:1), controlled, parallel, open, pragmatic, exploratory feasibility study with mixed-methods process evaluation
study population	N (total) = 215 (intervention n= 143; control n= 72)
inclusion/ exclusion criteria	**Practices:**<br><br>Inclusion criteria of principal investigator per trial center: General practitioner (specialist in General Practice and Family Medicine or internal medicine with primary care, general practitioner Doctor with 80 hours of basic training in "Psychosomatic Basic Care"

	Exclusion criteria: Practices with more than 50% of the care volume in specialized care (e.g. specialized outpatient palliative care, infectiology, addiction therapy, psychotherapy)<br><br>**Participants:**<br><br>patients (18 years and older) presenting to primary care with one or more non-medical health-related social problems.<br><br>Exclusion criteria: Patients who are under legal guardianship in relation to health, a person living in the same household is already included in the study
intervention	**Intervention:**<br><br>The intervention begins with the referral of patients with non-medical health-related social problems by general practitioners to a link worker . The main task of the link worker is to support the participating patients in connecting with existing, locally available non-clinical support services that can help improve the non-medical health-related problems (e.g. loneliness or financial worries). During the consultation, individual action plans are developed and implemented together with the participants. These are based on the needs and goals of the participants and include concrete measures to solve the problem. The general practitioners are informed via a feedback form which services in the area the patients have been referred to. The period in which link workers and participants have contact and the number of contacts is not limited in this feasibility study. 2-5 meetings are to be expected over a period of four to twelve weeks.<br><br>**Control:**<br><br>Usual care plus brochure with information about local non-clinical supports and services (“Treatment- as - usual +”).

endpoints	**Primary feasibility endpoints:**<br>Proportion of participants who have at least one appointment with the link worker (intervention arm only)<br>Proportion of participants who discontinue the study before 6 months of follow-up (loss to follow- up , both arms)<br>**Secondary feasibility endpoints:**<br>Acceptance:<br>Satisfaction with the intervention among general practitioners (last patient last visit questionnaire) and<br>participant satisfaction (3-month follow-up and 6-month follow-up)<br>Practicability:<br>Estimated time required by general practitioners<br>Proportion of eligible patients compared to the number of patients treated in the practice<br>Proportion of patients who were excluded due to a language barrier<br>Proportion of eligible patients who consent to the study<br>Proportion of consenting patients who are randomized<br>Proportion of randomized patients who attend scheduled appointments with the link worker (intervention arm only)<br>Need/Use (intervention arm only):<br>From GP: assessed need for SP of participants<br>Participants' SP needs assessed by Link Worker<br>Number of appointments with link workers per participant<br>Duration of appointments with link workers

	Form of interaction with the link worker (in person, home visit, in the GP's office, by telephone)<br><br>Offers mediated by link workers<br><br>Use of the recommended mediated offers<br><br>**Clinical Endpoints/ Patient Reported Outcome Measures (PROM), after 3 and 6 months:**<br><br>Health status (WHODAS 2.0) (main clinical endpoint after 6 months)<br><br>General life satisfaction (L-1)<br><br>Loneliness (De Jong Gierveld loneliness Scale short version)<br><br>Health-related quality of life (EQ-5D-5L)<br><br>well-being (WHO-5, ICECAP-A)<br><br>Subjective health status<br><br>Goal-based endpoint (Goal based outcome , GBO, intervention arm only, baseline assessment when determined with Link Worker)<br><br>Use of health and social services (excerpts from PECUNIA RUM/EHIS- GeDA)<br><br>Occurrence of adverse events (number of deaths, emergency room visits, unplanned hospitalizations, new-onset suicidality, suicide attempts, other adverse events)
number of cases	Since this is an exploratory feasibility study without confirmatory hypothesis testing, no formal sample size calculation will be performed. Based on previous experience, it is assumed that the analysis of a total of 215 participants (in a ratio of 2:1, i.e. n=143 in the SP arm and n=72 in the TAU+ arm) is sufficient to descriptively determine the feasibility and acceptability aspects of the study and to obtain further information for the planning of subsequent studies.

	A drop-out of approximately 30% is expected, so that follow -up data for secondary clinical endpoints of approximately 150 patients (n = 100 in the SP arm and n = 50 in the TAU+ arm) will be available after 6 months.<br><br>This results in:<br><br>Screening: n= 600<br><br>Recruitment: n= 300<br><br>Randomization: n=215<br><br>Analysis: n=150-215
statistics	All collected data are evaluated descriptively: means, standard deviation, median and quartile or frequencies and percentages (overall and separately for treatment groups).<br><br>Primary endpoints: The proportion of participants in the intervention group who attended at least one appointment with the link worker and the proportion of participants who discontinued the study before the 6-month follow-up (dropout rate) will be analyzed descriptively, with frequencies and percentages, including 95% confidence intervals per treatment group. The comparison of the dropout rate between the treatment groups will be done descriptively and using logistic regression to determine the possible influencing factors.<br><br>Secondary endpoints are analyzed descriptively (frequencies/percentages, mean/standard deviation or median/interquartile depending on scale and distribution, each with corresponding 95% confidence intervals).<br><br>Comparisons between treatment groups will be performed using logistic regression (binary endpoints) or analysis of covariance (continuous endpoints adjusted for baseline , if available), taking center into account.

	Safety: Adverse events are analyzed descriptively (frequencies and percentages per treatment group).<br>Basically, all results are interpreted exploratively.
Qualitative process evaluation	The process evaluation consists of a continuous quantitative analysis of the study documentation as well as qualitative interviews with the actors involved (patients, family doctors , link workers , representatives of local services, study team). A total of 30-40 participants are to be recruited, half of whom will be participants in the two study arms and half other actors involved. The interviews will be evaluated using thematic coding and triangulated with the quantitative analyses.
duration of study	**Total study duration :** 24 months<br>**First patient in to last patient out:** 12 months<br>**Recruitment period :** 6 months<br>**Duration of intervention :** 3 months
financing	German Research Foundation: application number HE 6399/3-1; project number 530364906

2 background

Non-medical health-related social problems are common in primary care (1) . They can have an impact on the occurrence of mental and physical illnesses (2–4) . However, such social problems can also be caused by (chronic) diseases. They are associated with significant economic costs, e.g. short-term work incapacity and long-term absence from work (5–7) . Non-medical health-related social problems are operationalized by the International Classification of Primary Care 3rd Edition (ICPC-3) in chapter ZC: "Social Problems" (8) and include problems such as loneliness, problems in the family and at work, and financial difficulties (1,9) . These problems disproportionately affect people with a lower socioeconomic status and thus reinforce health inequalities (10) .

Although non-medical health-related social problems are widespread in general practice, general practitioners can address and/or clarify only a few problems during their consultations (11) . However, in Germany, there is an extensive range of non-clinical support services and services available in the neighborhood. Nevertheless, there are no formalized links between these services and primary care (12) . Therefore, it is difficult for both general practitioners and patients to find the right contact point. Low-threshold, cooperative solutions are available here (1) .

Several possible solutions are being discussed scientifically: e.g. integrated primary care centers, social workers in primary care and social Prescribing (SP). SP was developed in the UK and has been implemented across the board in the National Health System (NHS) in recent years. Worldwide, SP programs have been introduced in several countries such as Canada, Austria and Singapore (13) . SP offers GPs a non-medical referral option that can accompany existing treatments to improve health and well-being (14) . SP is implemented through the involvement of a "link worker" to whom patients with non-medical health-related social problems are referred (= issuing a "social prescription"). Link workers are specially trained professionals or volunteers. Together with the patients, they develop an action plan and refer them to existing local services. The current evidence is based predominantly on controlled before-and-after studies; there are only a few controlled or randomized controlled studies (16) .

The available evidence indicates the need for randomized controlled trials to evaluate the effectiveness of SP - especially outside the UK. However, since there is only limited experience with establishing SP in Germany, a feasibility study is necessary to clarify various aspects of the study. Important points are the acceptance of the study, drop-out, especially in the control group, the extent of advice provided by the link worker in the German health and social system, and the feasibility of the endpoint measurements.

3 project goals and endpoints

The main objective of this feasibility study is to evaluate the feasibility of a randomized controlled trial on the "Social Prescription" (Social To evaluate the current clinical trial design (prescribing) in Germany and to provide information and data necessary for planning a confirmatory trial (e.g. recruitment and study procedures, feasibility of individual randomisation compared to cluster randomisation, sample size calculation).

The secondary objectives of the study include the assessment of acceptability, practicability and need/use of the intervention, as the study is a pilot of social Prescribing in Germany and these parameters are crucial for the design of future offers. The study will also examine the suitability of various endpoints that will be used in the future to determine the health effects of social Prescribing at the individual and health economic level. In addition, adverse events occurring within the study will be systematically recorded in order to make statements about the safety of social Prescribing to be able to meet.

The following table presents the objectives of the study with the associated quantitative endpoints.

Table 2: Project objectives and endpoints

Goals	endpoints
**main goal**	**Primary endpoints**
Determination of the feasibility and feasibility of a randomized controlled trial on the "social prescription"	Proportion of participants who have at least one appointment with the link worker (intervention arm only, within 6 months)
	Proportion of participants who discontinue the study before 6 months of follow-up (discontinuation rate, both arms)
**Secondary goals**	**Secondary endpoints**
Determination of Acceptance	Satisfaction of GPs (questionnaire after last patient last visit for all participating GPs)
	participant satisfaction (3 -month follow-up and 6-month follow-up)
Determination of practicality	Estimated time expenditure of the general practitioners (questionnaire after last patient last visit for all participating general practitioners)
	Proportion of eligible patients compared to the number of patients treated in the practice
	Proportion of patients who were excluded due to a language barrier
	Proportion of eligible patients who consent to the study
	Proportion of randomized participants who attend appointments agreed with link workers (intervention arm only)
Determination of needs and use	From GP: assessed need for SP of participants (intervention arm only)
	Participants' SP needs assessed by link worker (intervention arm only)
	Number of consultations with Link Worker per participant (intervention arm only)
	Duration of consultations with link workers (intervention arm only)

	Form of interaction with link worker (in person, home visit, in the practice, by telephone) (intervention arm only)
	by Link Worker (intervention arm only)
	Use of the recommended mediated offers/ brochure by the participants
Comparison of Social Prescription vs. TAU+ in clinical endpoints/ Patient Reported Outcome Measures (PROM)	Health status (WHODAS 2.0) (3-month follow-up and 6-month follow-up compared to baseline)
	Mental well-being (WHO-5) (3-month follow-up and 6-month follow-up compared to baseline)
	General life satisfaction (L-1) (3-month follow-up and 6-month follow-up compared to baseline)
	Loneliness (De Jong Gierveld Loneliness Scale) (3-month follow-up and 6-month follow-up in Comparison to baseline)
	Goal-based endpoint (GBO) (baseline assessment when determined with Link Worker , 3-month follow-up, 6-month follow-up, intervention arm only)
	Capability Well-being (ICECAP-A) (3-month follow-up and 6-month follow-up in Comparison to baseline)
	Health-related quality of life (EQ-5D-5L) (3-month follow-up and 6-month follow-up compared to baseline)
	Subjective health status (3-month follow-up and 6-month follow-up compared to baseline)
Comparison of Social Prescription vs. TAU+ in Health Economic Endpoints	Utilization behavior of health-related services, in particular the domains: hospital stays and inpatient care facilities, emergency care, outpatient health services and social services, medication, informal care, incapacity for work (3-month follow-up and 6-month follow-up compared to baseline)

Determination of Safety	number of deaths among participants
	Number of emergency room visits among participants
	Number of unplanned hospitalizations among participants
	Number of participants with (newly occurring) suicidality
	number of suicide attempts among participants
	Number of participants who experienced other adverse events

4 study design

4.1 study design

The study is designed as a multicenter, two-arm, open-label, exploratory, individually randomized, controlled, pragmatic feasibility study. SP will be compared with “treatment as usual ” plus a brochure with information on local out - of-hospital support and community services (TAU+) (see Figure 1).

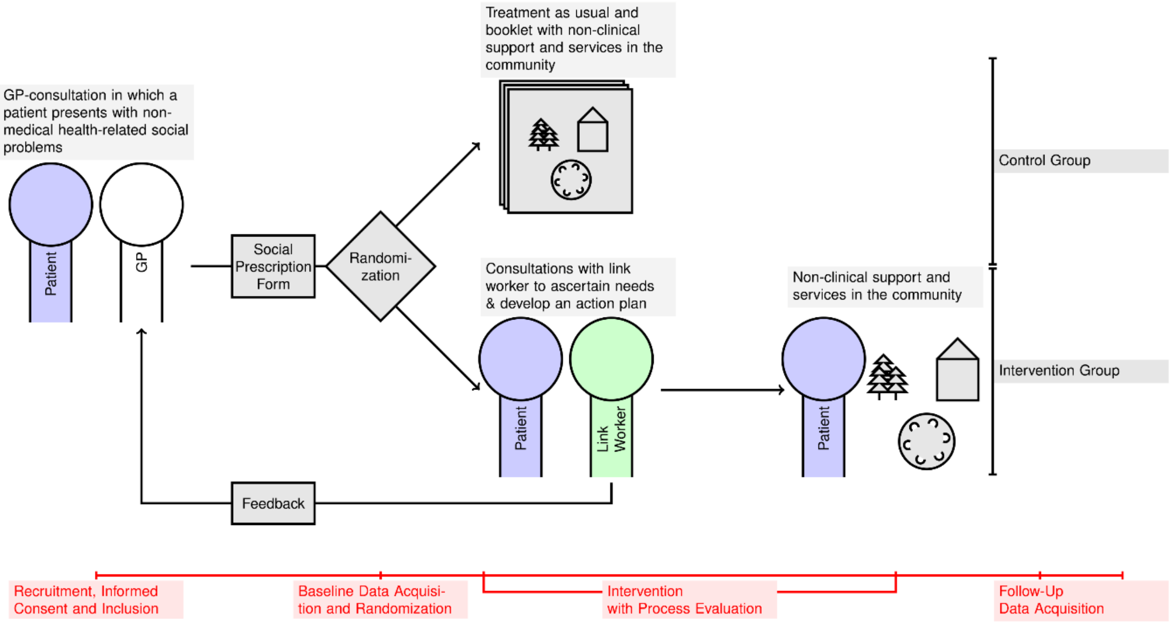

Figure 1: Study design

4.2 justification of the study design

We chose a multicenter approach to achieve the required sample size and to enable better generalizability of the results. In this feasibility study, the study centers are located in Berlin and Brandenburg. A randomized design is necessary to test the feasibility of individual randomization in primary care. We chose an allocation ratio of 2:1 to ensure that enough participants are available in the intervention arm for the key feasibility endpoints. A control group is required to provide information on the feasibility of the randomization, the proposed control group design, and the study procedures, particularly with regard to loss to follow- up in the control group. Contamination between the intervention and control groups within each practice will be analyzed qualitatively to assess the feasibility of individual randomization compared to cluster randomization.

The approach chosen was as pragmatic as possible, since the intention of the future confirmatory study will be to make real clinical decisions on the implementation of SP into routine primary care (17) .

4.3 intervention

, patients assigned to the intervention arm will receive counseling from a link worker employed at the Institute of General Practice and Family Medicine as part of the study .

Link workers are professionals who have at least a vocational qualification or a bachelor's degree in a field of health, healthcare, social work or social care. The link workers will be trained by the study team before the start of the intervention based on existing link worker training courses from Austria and the United Kingdom (18,19) .

The main task of the link workers is to support patients in connecting with existing, locally available non-clinical support services that can help solve the patients ' non-medical health-related problems (e.g. loneliness or financial worries). During the consultation, individual action plans are developed and implemented together with the patients. These are based on the patients ' needs and goals and include concrete measures to solve the problem. In addition to their consultation work, the link workers research local offers, enter them into an electronic database and network. The link workers are offered supervision by employees of the Institute of General Practice and Family Medicine.

In a first step, the link worker assesses the needs, agrees a goal and develops an action plan together with the patient. This action plan contains references to existing non-clinical support services and services in the neighbourhood. The link worker supports the patient in using the agreed services. At

the end of the intervention, the link worker provides feedback to the prescribing GP (15) . The contact between the link worker and the patient usually includes several consultations over several weeks.
The first appointment with the link worker is arranged with the patient immediately after the telephone baseline survey by the study nurse. The first appointment should take place no later than 14 days after recruitment into the study arm. The duration and number of appointments are not limited within the scope of this feasibility study and depend on the needs of the patients and the capacity of the link workers . We expect an average of 2-5 appointments per patient lasting up to 45 minutes over a period of four to 12 weeks. The consultation appointments usually take place on the premises of the study centers. At the patient's request, consultations can also be carried out by telephone or home visit if necessary.
As part of the consultation, link workers can pass on patient data to local providers (e.g. to make appointments) after the patient has given their express consent (implied consent) . After the consultation by the link worker has been completed, the treating GPs receive feedback on the consultation via a standardized form.
The following table describes the intervention of the Social Prescription (Social Prescribing) according to the TIDieR Framework.

Table 3: Intervention according to the TIDieR Framework

aspect		justification
**short name**	social affairs Recipe	
**Why**	Through social Prescribing allows patients with non-medical, health-related social problems to be referred to non-clinical support and services in the community.	This makes use of existing services.
**What**	For each community/neighbourhood, the Link Worker will set up a database containing information and contact details on the services required for social prescribing. This database builds on information provided in the leaflets used in the control arm. The database becomes continuously during the intervention expanded .	The database is intended to help develop a sustainable intervention.
	1. The GP issues an individual social prescription outlining the patient's problems and highlighting the main issues.<br>2. The link worker has several appointments with the patient. First, the patient's needs are determined, a main goal is set together and an action plan is developed as to which local support options the patient should be referred to.<br>3. The link worker ensures that the patient receives the agreed support options on site.<br>4. The link worker informs the GP where the patient has been referred using a standardised feedback form.	This concept is based on the definition of SP by Muhl et al. (14) and existing SP programs in the UK.

aspect		justification
**Who**	The link worker has an apprenticeship or a bachelor's degree in the health or social sector and has also received training as a link worker	Link workers require a basic understanding of health and social care.
**How**	Individual consultations preferably in person or alternatively by telephone.	Since the needs are very individual, tailor-made services are required.
**Where**	Preferably in the family doctor's office, alternatively as a home visit or by telephone.	This allows more flexibility and meets the needs of patients.
**When and how much**	The first consultation takes place no later than two weeks after study entry. The first consultation lasts a maximum of 45 minutes. The link worker and the patient arrange as many appointments as necessary, usually within a period of up to four weeks after study entry, up to a maximum of twelve weeks.	For the vulnerable patient group, tracking is particularly important when they reach the appropriate local services. Therefore, a tracking a integral be part of it .

The implementation of the intervention will take place in the *SocPres_SOP Implementation of the intervention* is described in detail.

4.4 control

Participants in the control group will receive a brochure with locally available non-clinical support services, which will be sent to the participants by the study nurse after randomization to the control group.

This process is described in the *SocPres_SOP Implementation of the intervention* is described in detail.

4.5 measures to reduce bias

4.5.1 Randomization and Allocation

Randomization in this study is done to test the feasibility of randomization for the main confirmatory study. That is, randomization is not done with the aim of testing comparative hypotheses.

The randomization code is created by an independent person from the Institute of Social Medicine, Epidemiology and Health Economics (who is not otherwise involved in the study) as a block randomization (with variable block length) stratified by study center (general practitioner's practice) with an allocation ratio of 2:1.

All patients who consent to participate and meet the inclusion criteria will be randomized after the baseline assessment. The randomization codes will be taken from the REDCap database by the study

nurse responsible for allocation , with allocation to the next patient being concealed . Allocation will therefore take place without any influence from the study management, the investigators or the link workers .

The procedure is described in the *SocPres_SOP Randomization and Allocation* .

4.5.2 blinding

Due to the study design, blinding of the investigators , link workers and patients is not possible. Due to the 2:1 randomization, blinding of the statistical analyses is also not possible.

4.6 study participants and centers

For the study , 300 adult patients with non-medical health-related problems (see section 4.5.2) will be recruited by their treating general practitioners . Recruitment will take place in nine study centers.

4.6.1 Inclusion and exclusion criteria for trial centers

Inclusion criteria: The principal investigator of the respective study center (general practitioner's practice) has the following qualifications:

- General practitioner (specialist in General Practice and Family Medicine or internal medicine with primary care, general practitioner doctor)
- 80 hours of basic training in "Psychosomatic Basic Care"

Exclusion criteria:

- Practices with more than 50% of their care volume in specialized care (e.g. specialized outpatient palliative care, infectious diseases, addiction therapy, psychotherapy)

The inclusion and exclusion criteria at practice level are intended to ensure generalisability to primary care.

4.6.2 Inclusion and exclusion criteria for patients

Inclusion criteria:

- patients (18 years and older) who are capable of consenting
- Presence of one or more non-medical health-related social problems (see below)

Exclusion criteria:

- patients who are legally cared for in relation to health
- a person living in the same household is already included in the study

Non-medical health-related social problems are defined by chapter ZC of the International Classification of Primary Care – 3rd Revision (ICPC-3) (8) .

The classification includes problems in the following areas:

- Financial problems (including poverty)
- Problems with social benefits (including lack of receipt of social benefits, problems with incapacity to work)
- problems at work
- Unemployment as a problem
- loneliness, social isolation
- Problems in the relationship with a partner, with a child, with parents or other family members, with other people (including bullying)
- Loss/death of a partner, child, parent or other family member
- Problems due to the illness of a partner, child, parent or other family member
- Problems related to education, school, training, or studies (including illiteracy, lack of school qualifications)
- problems due to discrimination
- Problems due to residence status/residence permit
- problems with experiences of violence
- housing problems (including homelessness or threatened homelessness)
- Legal problems (e.g. threatened prison sentence or similar)
- Problems with the health system (e.g. care
- Other social problems

4.7 patient pathway and visits

The visit overview in Table 4 and the CONSORT flow diagram (Figure 2) show the individual patient path through the study. The individual time points from Table 4 are described below.

Table 4: Visit overview

visit	t_{-1}	t_0	s_1	$s_2, s_3, \ldots, s_n$	t_1	t_2
	**consultation in the practice**	**baseline survey/randomization**	**First Link Worker Session**	**Follow up Link Worker sessions**	**3-month follow-up**	**6-month follow-up**
**inclusion**						
review of inclusion and exclusion criteria	X					
enlightenment	X					
consent	X					
Sociodemographic data		X				
randomization		X				
**treatment per study arm**						
TAU+ (brochure)		X				
Social prescribing		X	X	X	X	X
**survey instruments**						
feasibility endpoints	X	X	X	X	X	X
WHODAS 2.0		X			X	X
WHO-5		X			X	X
L-1		X			X	X
ICECAP-A		X			X	X
EQ-5D-5L		X			X	X
De Jong Gierveld Loneliness Scale		X			X	X
Target-based endpoint (intervention arm)			X		X	X
utilization behavior (PECUNIA-RUM)		X			X	X

Subjective health status		X			X	X
Security		X	X	X	X	X

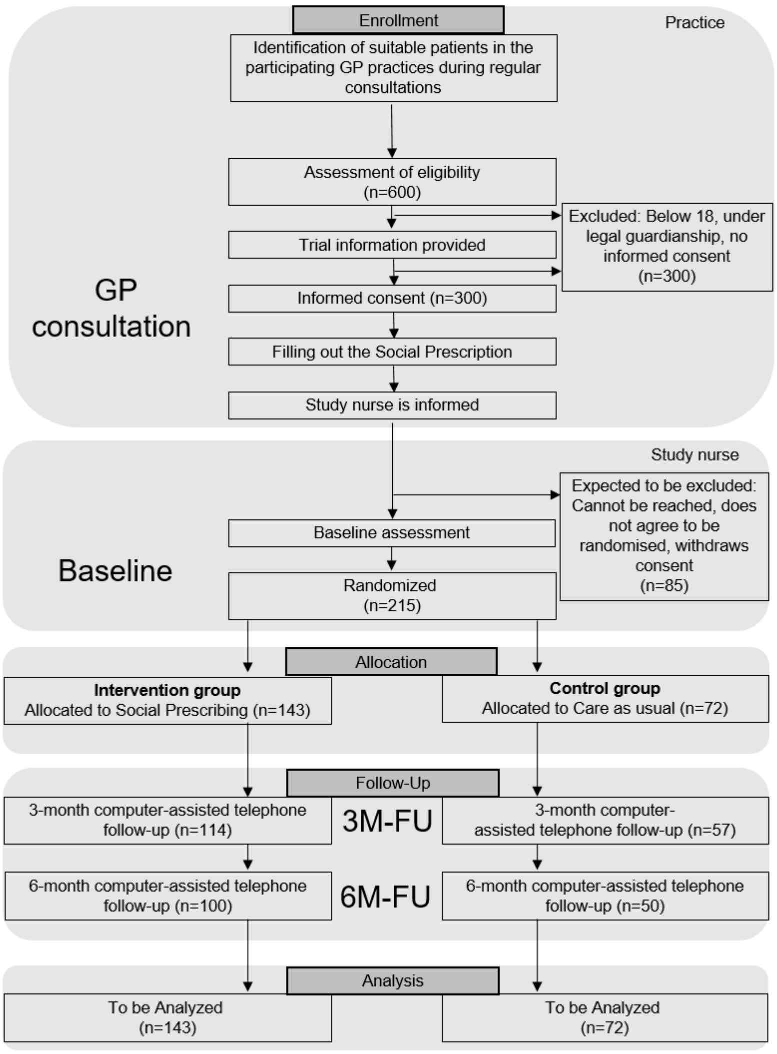

Abbildung 2: CONSORT-Flussdiagramm

4.7.1 t $_{-1}$: consultation in the practice

1. The starting point is when a non-medical health-related social problem is raised in a normal doctor-patient consultation with one of the investigators in one of the trial centers. Such problems can be, for example:
    - loneliness
    - financial problems
    - problems at work

- problems in the family
- problems with the apartment/living space

2. In this case, the investigator can offer the patient participation in the study. The investigator informs the patient about the study and documents this in his investigator-specific recruitment list.
3. If the patient is interested in participating in the study, the investigator checks the study's inclusion and exclusion criteria and provides verbal and written information about the study using the study information. The patient is given a participant information sheet.
4. The patient is given as much time to think about it as he or she wants. This means that he or she can give his or her consent directly during this consultation or at a later date.
5. If the patient agrees to participate, the patient and the investigator sign the consent form in duplicate. The original remains at the trial center. The patient receives the second copy of the signed consent form.
6. The data of the included patients are then transmitted by the investigator to the Charité. Various secure transmission channels are available.

The details of the procedures at t_{-1} are described in detail in the *SocPres_SOP practice consultation* .

4.7.2 t_0 : Baseline data collection and randomization

1. Once the data of a new study participant has been received, the study team at the Charité is automatically informed and a study nurse takes over the care of this study participant.
2. The study nurse should contact the study participant within 1-2 weeks after inclusion.
3. To collect baseline data, the study nurse conducts a computer-assisted telephone interview (CATI) with the study participant. If this is not possible due to limitations on the part of the participant, the baseline data collection can, as an exception, also be carried out by the study nurse during a home visit, at the Charité or during a practice visit.
4. After completion of baseline data collection, the study nurse randomly assigns subjects to a study arm.
5. If study participants are assigned to the intervention arm, the study nurse will arrange the first appointment with the link worker within 7 days of randomization if possible. If study participants are assigned to the control arm, she will send the study participant the brochure with local support services.

The details of the procedures at t_0 are described in detail in the *SocPres_SOP data collection* .

4.7.3 t_1 follow-up survey after three months

The first follow-up survey will take place no earlier than three months after the randomization of the study participant. It will be conducted by the study nurse as a CATI or, in exceptional cases, during a home visit, at the Charité or during a doctor's office visit . The survey will take place as soon as possible at the three-month appointment, but no later than four months after randomization.

The details of the processes at t_1 are described in detail in the *SocPres_SOP data collection* .

4.7.4 t_2 Follow-up survey after six months

The second follow-up survey will take place at least six months after the randomization of the study participant. It will be conducted by the study nurse again as a CATI or, in exceptional cases , during a home visit, at the Charité or during a doctor's office visit . The survey will take place as soon as possible at the six-month appointment, but no later than seven months after randomization.

The details of the processes at t_2 are described in detail in the *SocPres_SOP data collection* .

4.8 Medical care

The medical care of the participants by their family doctors remains unaffected by randomization into one of the two study arms and is provided without any restrictions throughout the entire period of the study.

5 termination criteria

5.1 termination criteria for individual study participants

1. Study participants can terminate their participation in the study at any time without giving reasons.
2. Investigators may stop the study for a participant if the risk to that person outweighs the individual benefit.

5.2 Termination criteria for parts of the study or the entire study

1. In case of repeated violations of the trial plan (e.g. allocation), the sponsor representative can close individual trial centers.

2. The Data Safety and Monitoring Board (DSMB, see below) may recommend stopping recruitment of participants for either all or some treatment groups and/or some subgroups of participants. The sponsor representative decides on termination.

6 data collection

6.1 data sources

6.1.1 Personally Identifiable Data

by the family doctors are securely transferred to the Charité by the practice staff.

6.1.2 health data

The trial centers also collect and transmit other health data of the participants (long-term diagnoses, reason for the social prescription).

6.1.3 survey data from family doctors

After the end of the study at the respective trial center, the participating general practitioners will be surveyed via online questionnaire.

6.1.4 recruitment list

The information relevant to the feasibility endpoints in the recruitment lists completed by the investigators is regularly queried by the Charité.

6.1.5 Adverse events

Adverse events are reported by the GP to the study team within seven days. Various secure transmission channels are available for this purpose, which are described in more detail in the associated SOP *SocPres_SOP Reporting, evaluation and handling of adverse events* . Adverse events are stored in a central database (see Adverse events).

6.1.6 patient survey data

Study nurses contact the patients recruited for the study using the contact details entered by the practices in the personal identification database (PiD) . All survey data from participants are collected through a computer-assisted telephone interview (CATI) after the participant has been included in the

study (baseline), after 3 months (3-month follow-up) and after 6 months (6-month follow-up) and entered directly into the eCRF .

6.1.7 documentation of the Link Worker

After the consultations with the participants, the link workers document the content of the conversation, the offers made and the duration of the consultation in the eCRF in REDCap .

This process is described in the *SocPres_SOP Implementation of the intervention* is described in detail.

6.2 data with and without source data

The following data refers to source data: Personal data, contact details and long-term diagnoses of the patients (source data: patient file /practice management software) and adverse events (source data: doctor's letters for treatment in the emergency room or hospitalization, documentation in the practice management software). For all other data, the eCRF is the source document.

6.3 archiving and access to source data

The investigators grant study-specific access to source data and consent forms for monitoring, audits and controls by the ethics committees for up to ten years after completion of the studies.

6.4 instruments

Sociodemographic data: Based on the European Health Interview and Examination Survey (EHIS) and the Diversity Minimal Item Set (DiMiS), sociodemographic data (e.g. age, gender, school education, occupation, gender identity and migration background) are collected (20) . In addition , the treating general practitioners record any existing long-term diagnoses of the participants.

Health status (WHODAS 2.0): The WHO Disability Assessment Schedule 2.0 is a generic patient-reported outcome measure of health status. The domains of WHODAS 2.0 are based on the conceptual framework of the WHO International Classification of Functioning, Disability and Health (ICF), which has been the international standard for describing and measuring health and disability since 2001. All six ICF domains (cognition, mobility, self-care, social interaction, life activities and social participation) are covered by the WHODAS 2.0 instrument. It shows high internal consistency, high test -retest reliability and high validity compared to other instruments (21,22) . A computer-based adaptive form with 12 items is available in German.

Psychological well-being (WHO-5): The World Health Organization -Five Well- Being Index (WHO-5) is a brief, self-reported measure of current psychological well-being. The scale has sufficient validity as an outcome measure in clinical trials (23) , and normative data are available for a German version (24) .

General life satisfaction (short scale L-1 to measure general life satisfaction): The short scale L-1 for assessing general life satisfaction consists of the item formulation established in the SOEP (25,26) . The scale contains only one item with the following wording: "How satisfied are you with your life overall at present?" The instrument has been validated in German with good reliability and normative data are available (25) .

Loneliness (De Jong Gierveld loneliness Scale): The short version of the De Jong Gierveld loneliness Scale comprises 6 items and includes two subscales on emotional and social loneliness (27) . The questionnaire is valid and reliable. A validated German translation exists.

Capability Well- being (ICECAP-A): The ICEpop CAPability measure for Adults is a measure of well-being of the general adult population (aged 18 years and over) for use in health economic evaluation. A validated German translation exists (28) .

Health-related quality of life (EQ-5D-5L) : The European Quality of Life 5 Dimensions 5 Level Version is a general instrument that can be used to assess the quality of life of patients regardless of their disease. It has been extensively validated, including in the German health care context (29) .

Subjective health status: The health status is questioned based on the European Health Interview and Examination Survey (EHIS) as follows: How would you describe your health status in general?

Goal-based outcome (GBO) : GBOs are person-centered endpoints. Link workers and participants agree on a main goal to be achieved. Follow- ups then clarify whether this goal has been achieved or how far the person is from achieving it. The GBO is only measured in the intervention group.

Utilization behavior (relevant sections from PECUNIA RUM or EHIS- GeDA) : The PECUNIA instrument for measuring resource utilization (PECUNIA RUM) measures resource utilization in all sectors of the adult population that are relevant for cost calculation from a societal perspective: health and social care, education, (criminal) justice, productivity losses and informal care (30,31) . In the present study, selected items from this measurement instrument are used that relate to the topics

examined in the study on health and social care, as well as in the context of productivity losses, in particular hospital stays and inpatient care facilities, emergency care, outpatient health services and social services, medication, informal care, incapacity for work. If possible, the culturally adapted version of EHIS- GeDA for Germany is used.

7 data protection

7.1 data protection concept

A data protection concept was drawn up for the study and reviewed and advised by the Clinical Trial Office of the Charité Universitätsmedizin Berlin.

7.2 contact persons for data subject rights

The contact details of the contact persons for data subjects' rights will be communicated to the study participants in the study information.

8 biometrics

8.1 Generally

In this feasibility study, all results are interpreted exploratively.

The details of the statistical analyses (including definition of analysis populations, subgroups, sensitivity analyses, etc.) will be specified in a statistical analysis plan (SAP) before data analysis. No interim analysis is planned.

8.2 case number estimate

Since this is an exploratory feasibility study without confirmatory hypothesis testing, no formal sample size calculation will be performed. Based on previous experience, we assume that the analysis of a total of 215 participants (in a ratio of 2:1: 143 in the SP arm and 72 in the TAU+ arm) is sufficient to descriptively determine the feasibility and acceptability aspects of the study and to obtain further information for planning the subsequent study.
A drop-out of approximately 30% is expected, so that follow -up data for secondary clinical endpoints of 150 patients (n = 100 in the SP arm and n = 50 in the TAU+ arm) will be available after 6 months.

8.3 evaluation populations

Analyses using the Full Analysis Set (FAS) are performed according to the intention - to - treat principle with all available data (missing data are not imputed).

The per-protocol population is defined as patients who adhere to the protocol (details are defined in the SAP).

8.4 analyses

Primary endpoints: The proportion of participants in the intervention group who attended at least one appointment with the link worker and the proportion of participants who discontinued the study before the 6-month follow-up (dropout rate) will be analyzed descriptively, with frequencies and percentages, including 95% confidence intervals per treatment group. The comparison of the dropout rate between the treatment groups will be done descriptively and using logistic regression to determine possible influencing factors.

Secondary endpoints are analyzed descriptively (frequencies/percentages, mean/standard deviation or median/interquartile depending on scale and distribution, each with corresponding 95% confidence intervals).

Comparisons between treatment groups will be performed using logistic regression (binary endpoints) or analysis of covariance (continuous endpoints adjusted for baseline , if available), taking center into account.

Safety: Adverse events are analyzed descriptively (frequencies and percentages per treatment group).

8.5 sensitivity analyses

Relevant endpoints will additionally be analyzed using the per-protocol population.

Subgroup analyses are carried out for relevant endpoints. The following subgroups are used, among others:

- Gender
- underlying psychosocial problem(s)
- age groups.

This is also done descriptively and with logistic regression or analysis of covariance with an additional factor interaction (treatment group*subgroup) in the model. Details of the definitions of the subgroups are described in the SAP.

If there are relevant differences between the groups at baseline, these variables are additionally adjusted.

Clustering by center/physician or link worker is taken into account with different models (e.g. fixed/random effects).

9 data management

Data management is carried out in accordance with the specifications of the quality management manual of the Institute for Social Medicine, Epidemiology and Health Economics. The data is only stored and processed for the respective purpose. The applicable data protection laws are observed.

9.1 collection and storage of data

In the consent form to participate in the study, the study participant agrees to the recording, processing and storage of his/her personal and medical data as part of this survey.

The data collected as part of the study are recorded and stored in the REDCap online database (REDCap = Research Electronic Data Capture, version 14.0 or higher). The REDCap used to collect the data runs on virtual machines in the Charité IT infrastructure. The REDCap web server (accessible via https://redcap.charite.de) is located in the so-called DMZ (demilitarized zone) behind a firewall.

Personally identifiable data (PiD) are entered into REDCap by the practice staff . The research data (FD) are collected and stored separately from the PiD in another independent REDCap project; no PiD is transmitted to the survey project.

The databases are only accessible to data management and authorized study personnel with individualized login via 2-factor authentication.

The study data and study documents will be archived for 10 years after the end of the study and all personally identifiable documents and data will then be deleted. All research data will be anonymized 10 years after the end of the study.

9.2 pseudonymization

The data are recorded in REDCap using pseudonyms, which consist of consecutive numbers without initials or birth dates of the participants. The PiD database is the re-identification list for the pseudonymized FD entered by the study staff in REDCap .

9.3 Rights of participants (revocation, data deletion, right to information, correction)

Participants can object to further processing of their data at any time.

Participants have the right to request the immediate deletion of the data collected about them as part of the study.

There is a right to information about all study data collected. Participants have the right to information about and correction of study data collected about them at any time.

10 Qualitative process evaluation

The study is complemented by a qualitative process evaluation in which semi-structured episodic interviews are conducted with stakeholders involved in the study (patients, general practitioners , representatives of local services and members of the study team).

10.1 Goals and Topics of Qualitative Process Evaluation

Table 5 presents the objectives and topics of the qualitative process evaluation.

Table 5: Objectives and topics of qualitative process evaluation

**Objectives Qualitative Process Evaluation**	**Topics Qualitative Process Evaluation**	**each for:**
The qualitative semi-structured episodic interviews are intended to present the perspectives of the actors involved (patients, general practitioners , link workers and representatives of local services) in order to	Acceptance of study procedures (including randomization) and the intervention	patients
		family doctors
		Link Worker
		representatives of local offers
		study team

qualitatively assess the feasibility of the study, gain insights for future studies and care services and identify indications of contamination.	Practicability of study procedures and intervention	patients
		family doctors
		Link Worker
		representatives of local offers
		study team
	Need for and use of the intervention	patients
		family doctors
		Link Worker
		representatives of local offers
		study team
	Potential for the implementation of the intervention	patients (intervention arm)
		family doctors
		Link Worker
		representatives of local offers
		study team
	hurdles to the implementation of the intervention	patients (intervention arm)
		family doctors
		Link Worker
		representatives of local offers

		study team
	feeling of being part of the neighborhood	patients

10.2 Recruitment for qualitative process evaluation

exclusion criteria of the main study continue to apply to patients participating in the process evaluation . Patients can optionally give their consent to be contacted for recruitment for the qualitative process evaluation on the consent form of the main study. Selected patients will be contacted by the qualitative researchers within a period of up to 1 year after completion of the intervention and, if they are interested, will be informed. Participating patients will receive a signed consent form and study information for the qualitative process evaluation.

Selected general practitioners , link workers , representatives of local services and members of the study team will be asked about their interest in participating in the qualitative process evaluation during and after the study via communication channels established within the study.

Inclusion criteria:

- Participants are adults capable of giving consent (18 years and older)

Exclusion criteria:

- Participants who are legally cared for in relation to their health

We aim to include n=30-40 participants in the qualitative process evaluation, half of whom will be participants in both study arms and half other study stakeholders. Sampling will be carried out step by step based on theoretical sampling until theoretical saturation is reached.

10.3 Data collection, data storage and data analysis for qualitative process evaluation

Based on the defined goals and topics of the qualitative process evaluation, interview guidelines are developed by researchers at the Charité Institute of General Practice and Family Medicine. In addition to the questions in the interview guide, socio-demographic information (age, gender, school education and occupation) is requested. The researchers conduct the interviews, record them and transcribe them. The interview takes place in person at the Charité's central campus, in the general practitioner's office, at the participants' homes or, at the participants' request, at a neutral location of their choice.

In exceptional cases, a telephone interview is also possible upon request. The recordings and transcribed interviews are stored pseudonymously on a protected Charité internal drive. The interviews are evaluated using thematic coding (32) .

As part of the mixed methods approach, the qualitative interviews are recorded in parallel with the quantitative endpoints. The results of the evaluation are triangulated based on the quantitative and qualitative analyses of, among others, acceptance, practicability and need/use.

The concrete procedures for qualitative process evaluation are described in detail in the *SocPres_SOP Qualitative Process Evaluation* .

11 Accompanying health economic evaluation

The feasibility study is accompanied by a health economic evaluation, which uses the quantitatively collected data from the study. The aim of this is, on the one hand, to check the acceptance of the survey instruments on well-being (ICECAP-A), quality of life (EQ-5D-5L) and resource utilization (PECUNIA RUM/EHIS- GeDA). In addition, the link worker documentation is used to extract which offers were provided to patients and how the patient -link worker interaction went. The service utilization recorded is also evaluated in a group comparison in order to identify service areas with a significant group difference. The exploratory evaluations use the 5% and 10% significance levels. The findings will be used to revise the survey instruments for a follow-up study, i.e. to delete aspects in the PECUNIA RUM instrument that are less important in the application context of "social prescription" and to include the spectrum of offers provided outside the healthcare system for standardized documentation and determination of standard costs.

In addition to the results related to this research process, the data collected on service utilization as well as on quality of life and well-being are evaluated in terms of content. For the monetary assessment of resource use, the standard costs for all (privately purchased or prescribed) services and goods are calculated in euros. The cost-effectiveness of the intervention is determined using the net benefit approach. The statistical evaluation of the data will be carried out using parametric or non-parametric methods, depending on the distribution of the data. Finally, relevant subgroups from a health economic perspective will be identified and statements will be made on the power of the results achieved in order to support the sample size estimates for a potential follow-up study.

12 quality management and assurance

The study is being conducted in accordance with the ICH-GCP guidelines. The mandatory consultation was carried out by the QA Unit on May 17, 2024, with a certificate dated July 15, 2024. The sponsorship authorization required in this context according to the specifications of the Charité QA Unit was granted to the study director on July 22, 2024 by the Dean of the Charité - Universitätsmedizin Prof. Dr. Joachim Spranger.

12.1 Standard Operating Procedures (SOPs)

The study is based on quality management with SOPs in accordance with the study implementation under professional law, which specifies the necessary processes for carrying out the tasks of the study management or sponsor tasks and in particular for sponsor oversight .

12.2 monitoring

According to ICH-GCP, monitoring (study supervision) should ensure that

(a) ‘the rights and well-being of the subjects are protected.

(b) the study data reported are accurate, complete and verifiable using source documents.

(c) the study is conducted in accordance with the currently approved protocol/amendments, good clinical practice and applicable regulatory requirements.” (ICH-GCP, 5.18.1)

As part of the risk assessment, the risk of adverse events and risks to the rights and well-being of the trial subjects were assessed as very low. However, due to the limited prior experience of the trial centers, risks of errors in study documentation and deviations from the protocol by the trial centers were considered to be quite possible. According to ADAMON, we therefore decided on a risk-adapted approach at level K2 (33) .

In summary, monitoring consists of central monitoring (Central) and on-site monitoring (On Site). In addition, monitoring is carried out by the study coordination, an independent special monitor and co-monitoring by the Clinical Trials Office (CTO) of the Charité. The exact procedure is described in the SocPres_Monitoringplan .

13 Security

13.1 Adverse events

In the literature on social Prescribing does not describe any adverse events associated with the intervention. However, to demonstrate safety, a comprehensive monitoring procedure will be established in accordance with ICH-GCP , which will consist of short-term reporting of individual case reports, periodic reports, safety monitoring by an independent committee (DSMB, see below) and a comprehensive safety analysis of the study.

13.1.1 definition of adverse events

Since this is another study according to Section 15 of the Professional Code of Conduct for Doctors with a complex intervention without a drug or medical device, a classification of adverse events according to Common Terminology Criteria for Adverse Events (CTCAE) v5.0. Instead, the following adverse events are predefined for the study:

1) Death (any cause)

2) Emergency room visit (any cause)

Visit an emergency room. The report should be made regardless of whether the person is subsequently admitted to hospital or not.

3) Unplanned hospitalization (any cause)

An unplanned hospitalization is characterized by a hospital admission without a prior appointment.

4) Suicide attempt/suicidality

A suicide attempt is defined as a self-performed, harmful behavior with the intention of causing death, but which does not result in death (ICD-11: MB23.R, ICPC-3: PD14).

In this context, suicidality is understood as thoughts, ideas or ruminations about the possibility of ending one's own life, ranging from the consideration that one would be better off dead to the formulation of elaborate plans (ICD-11: MB26.A, ICPC-3: PS05).

5) Other relevant adverse events

To give investigators the opportunity to report further adverse events that they consider relevant, there is a separate category.

13.1.2 reporting of adverse events

Adverse events are reported using the form *SocPres_ReportingFormEvents* via secure communication channels by the investigators to the study management within 7 days of becoming known. The exact procedure is specified in the *SOP SocPres_SOP : Reporting, evaluation and handling of adverse events* .

13.1.3 Central documentation of adverse events

All adverse events are recorded centrally by the study management in a database: *SocPres_LineListing* and tracked until the final evaluation.

13.1.4 second assessment of adverse events

The secondary assessment of adverse events is carried out using the form *SocPres_EvaluationFormEvents* by the study management within 7 days of submission by the investigators . The procedure is defined in the SOP *SocPres_SOP Reporting, assessment and handling of adverse events* .

13.2 reporting

After recruitment of 50 patients each , or at least every three months, a safety report with the reported adverse events is prepared and submitted to the DSMB.

13.3 Data Safety Monitoring Board (DSMB)

The objectives of the DSMB are

- protecting the interests of study participants,
- the evaluation of the safety and effectiveness of the intervention during the study and
- monitoring the implementation of the study.

The DSMB receives and reviews information on the progress and data generated during the study.

This includes:

1. the assessment of data quality, including completeness,

2. monitoring recruitment numbers and loss to follow up ,
3. monitoring compliance with the study protocol by participants and examiners ,
4. monitoring of adverse events,
5. monitoring compliance with previous DSMB recommendations,
6. to consider the ethical implications of all DSMB recommendations.

The DSMB should decide whether to recommend continuing recruitment of participants into the study or to stop recruitment for either all or some treatment groups and/or some subgroups of participants . The DSMB's recommendations are advisory and not executive.

The composition of the DSMB, its working methods (including decision-making) and reporting are defined in the *SocPres_DSMB Charter* .

14 Ethical considerations

The intervention is considered to be low risk for patients because no adverse events were reported in previous studies, although these may not have been adequately recorded.

14.1 Individual Benefit

The potential benefit is an improvement in individual health and well-being.

Participants in the intervention group benefit from the opportunity to attend several appointments with a link worker and to use person-centered interaction to find out and articulate their own needs and implement solutions. The support is intended to reduce hurdles and increase the likelihood that participants will be able to take advantage of the services on offer. It is expected that communication with the link worker itself will have an individual therapeutic benefit (motivational interviewing), but also in particular referral to local services (e.g. through increased social contacts as part of participation in a choir to reduce loneliness or the use of debt counseling for financial problems).

Participants in the control group benefit from the opportunity to communicate their medical and social needs. However, apart from the dissemination of information about local services (" signposting ") so that participants can seek advice or use services themselves, no individual benefit in addition to regular care is to be expected for this group.

14.2 group benefit

In the long term, the results of the study will serve to improve social and health care for people with non-medical health-related needs. The high-quality, evidence-based results are intended to inform political decision-makers about possible measures to reduce health inequalities and improve health status, well-being and other patient-relevant endpoints. The results may enable the implementation of the social prescription as an integrated care model in regular care. This could mean that the concept could benefit all people with non-medical health-related problems who have access to primary care. It is also potentially possible to expand it outside of primary care (e.g. through pharmacies, emergency rooms, but also municipal services such as the employment agency, the citizens' office or the library). This is already being practiced in other countries.

There is also the potential that the intervention will result in lower use of healthcare, particularly primary care, but also the use of emergency departments. This has the potential to relieve the burden on the healthcare system.

14.3 Benefits for participating trial centers

The participating study centers will receive per recruited:n Patient:in an expense allowance of 50 EUR.

14.4 damage/risk

For study participants in the control and intervention groups, participation requires a time investment, including for the surveys.

For participants in the intervention group, it is conceivable that the conversations with the link worker could be emotionally upsetting and could therefore lead to decompensation, for example. However, such undesirable effects have not been reported in previous studies and in the widespread implementation in Great Britain. On the contrary, the conversations are seen as more of a relief.

14.5 Summary Assessment

In summary, we conclude that the benefits outweigh the risks.

14.6 Responsible Ethics Committees

The initial approval vote is given by the leading ethics committee of the Charité Universitätsmedizin Berlin. The additional vote in accordance with the legal requirements for the two trial centers in

Brandenburg is given by the ethics committee of the Brandenburg State Medical Association as the participating ethics committee.

15 insurance of study participants

In consultation with the Legal Department of the Charité – Universitätsmedizin Berlin, insurance coverage for the study is provided under the Charité’s existing business liability insurance from HDI-Gerling AG (insurance policy no.: 56-99629701016) (*letter from the Insurance Department dated July 19, 2024*).

16 publication guidelines

The following main publications are planned:

- Publication on the feasibility of the intervention with the primary endpoints
- Publication on clinical efficacy based on clinical endpoints
- publication on safety and adverse events
- publication on process evaluation

The principal investigator and deputy principal investigator are involved as authors in all publications. The biostatistician is involved in all publications with quantitative data. The principal investigators are involved as authors in all publications with quantitative patient data if they meet the criteria of the DFG's "Guidelines for Safeguarding Good Scientific Practice".

A publication plan is created and regularly updated, which contains the regulations on authorship . The regulations are based on the "Guidelines for Safeguarding Good Scientific Practice" of the DFG and the recommendations of the International Committee of Medical Journal Editors.

17 references

1. Zimmermann T, Mews C, Kloppe T, Tetzlaff B, Hadwiger M, von dem Knesebeck O, et al. [Social problems in primary health care - prevalence, responses, course of action, and the need for support from a general practitioners' point of view]. Z Evidence training torment in the healthcare system. 2018 Apr;131-132:81-9.

2. Holt-Lunstad J, Smith TB, Layton JB. Social Relationships and Mortality Risk: A Meta-analytic Review. PLOS Med. 2010 Jul 27;7(7):e1000316.

3. Valtorta NK, Kanaan M, Gilbody S, Ronzi S, Hanratty B. Loneliness and social isolation as risk factors for coronary heart disease and stroke: systematic review and meta-analysis of longitudinal observational studies. Heart. 2016;102(13):1009–16.

4. Lorant V, Croux C, Weich S, Deliège D, Mackenbach J, Ansseau M. Depression and socio-economic risk factors: 7-year longitudinal population study. Br J Psychiatry. 2007;190:293–8.

5. North FM, Syme SL, Feeney A, Shipley M, Marmot M. Psychosocial work environment and sickness absence among British civil servants: the Whitehall II study. At J Public Health. 1996;86(3):332–40.

6. Shiels C, Gabbay MB, Ford FM. Patient factors associated with duration of certified sickness absence and transition to long-term incapacity. Br J Gen Pract. 2004;54(499):86–91.

7. Herrmann WJ, Haarmann A, Bærheim A. Incapacity for work regulations as a factor for the utilization of medical care in Germany. Z Für Evidenz Fortbild Qual Im Gesundheitswesen. 2015 Jan 1;109(8):552–9.

8. ICPC-3 Consortium of the World Organization of Family Doctors. Chapter ZC Social problems influencing health status. In: van Boven K, Ten Napel H, editors. ICPC-3 International Classification of Primary Care: User Manual and Classification. CRC Press; 2021.

9. Herrmann WJ, Oeser P, Buspavanich P, Lech S, Berger M, Gellert P. Loneliness and depressive symptoms differ by sexual orientation and gender identity during physical distancing measures in response to COVID-19 pandemic in Germany. Appl Psychol Health Well-Being. 2023;15(1):80–96.

10. Marmot M. Achieving health equity: from root causes to fair outcomes. The Lancet. 2007 Sep 29;370(9593):1153–63.

11. Zantinge EM, Verhaak PFM, Kerssens JJ, Bensing JM. The workload of GPs: consultations of patients with psychological and somatic problems compared. Br J Gen Pract. 2005;55(517):609–14.

12. Popay J, Kowarzik U, Mallinson S, Mackian S, Barker J. Social problems, primary care and pathways to help and support: addressing health inequalities at the individual level. Part II: lay perspectives. J Epidemiol Community Health. 2007;61(11):972–7.

13. Morse DF, Sandhu S, Mulligan K, Tierney S, Polley M, Chiva Giurca B, et al. Global developments in social prescribing. BMJ Glob Health. 2022 May;7(5):e008524.

14. Muhl C, Mulligan K, Bayoumi I, Ashcroft R, Godfrey C. Establishing widely accepted conceptual and operational definitions of social prescribing through expert consensus: a Delphi study. BMJ Open. 2023 Jul 1;13(7):e070184.

15. Islam MM. Social Prescribing—An Effort to Apply a Common Knowledge: Impelling Forces and Challenges. Frontline Public Health. 2020;8:515469.

16. Napierala H, Krüger K, Kuschick D, Heintze C, Herrmann WJ, Holzinger F. Social Prescribing: Systematic Review of the Effectiveness of Psychosocial Community Referral Interventions in Primary Care. Int J Integr Care. 2022 Aug 19;22(3):11.

17. Zwarenstein M. 'Pragmatic' and 'explanatory' attitudes to randomized trials. JR Soc Med. 2017 May 1;110(5):208–18.

18. NHS England. Workforce development framework: social prescribing link workers [Internet]. [cited 2023 Feb 9]. Available from: https://www.england.nhs.uk/long-read/workforce-development-framework-social-prescribing-link-workers/

19. Gesundheit Österreich GmbH. Information and support tools on social prescribing [Internet]. [cited 2024 Aug 25]. Available from: https://goeg.at/SocialPrescribing_Dokumente

20. Stadler G, Chesaniuk M, Haering S, Roseman J, Straßburger VM, Martina S, et al. Diversified innovations in the health sciences: Proposal for a Diversity Minimal Item Set (DiMIS). Sustain Chem Pharm. 2023 Jun 1;33:101072.

21. Federici S, Bracalenti M, Meloni F, Luciano JV. World Health Organization disability assessment schedule 2.0: An international systematic review. Disable Rehabil. 2017 Nov;39(23):2347–80.

22. Ustün TB, Chatterji S, Kostanjsek N, Rehm J, Kennedy C, Epping-Jordan J, et al. Developing the World Health Organization Disability Assessment Schedule 2.0. Bull World Health Organ. 2010 Nov 1;88(11):815–23.

23. Topp CW, Østergaard SD, Søndergaard S, Bech P. The WHO-5 Well-Being Index: A Systematic Review of the Literature. Psychother Psychosom 2015;84(3):167–76.

24. Brähler E, Mühlan H, Albani C, Schmidt S. Test statistical testing and standardization of the German versions of the EUROHIS-QOL quality of life index and the WHO-5 well-being index. Diagnostica. 2007 Apr;53(2):83–96.

25. Richter D, Metzing M, Weinhardt M, Schupp J. SOEP scales manual [Internet]. Berlin: German Institute for Economic Research (DIW); 2013 [cited 2023 Feb 9]. (SOEP Survey Papers; vol. 138). Available from: https://www.econstor.eu/bitstream/10419/85279/1/770557678.pdf

26. GESIS-Leibniz Institute for Social Sciences. ALLBUS/GGSS 1980-2012 (Cumulated German General Social Survey 1980-2012) [Internet]. GESIS Data Archive; 2014 [cited 2023 Feb 6]. Available from: https://search.gesis.org/research_data/ZA4578?doi=10.4232/1.11898

27. De Jong Gierveld J, Van Tilburg T. The De Jong Gierveld short scales for emotional and social loneliness: tested on data from 7 countries in the UN generations and gender surveys. Your J Ageing. 2010 Jun;7(2):121–30.

28. Polley M, Richards R. A guide to selecting patient reported outcome measures (PROMs) for social prescribing [Internet]. University of Westminster, London; [cited 2022 Sep 28]. Available from: https://www.london.gov.uk/sites/default/files/a_guide_to_selecting_outcomes_measures_in_social_prescribing_final.pdf

29. Richmond Group. Social prescribing: Options for outcome measurement A Summary [Internet]. [cited 2022 Sep 28]. Available from: https://richmondgroupofcharities.org.uk/sites/default/files/dtrt_summary_of_learning_about_outcomes_measurement_for_social_prescribing.pdf

30. Jolliffe R, Seers H, Jackson S, Caro E, Weeks L, Polley MJ. The Responsiveness, Content Validity, and Convergent Validity of the Measure Yourself Concerns and Wellbeing (MYCaW) Patient-Reported Outcome Measure. Integr Cancer Ther. 2015 Jan 1;14(1):26–34.

31. Law D, Jacob J. Goals and goal based outcomes (GBOs) Some Useful Information [Internet]. Third edition. CAMHS Press; 2015 [cited 2022 Sep 28]. Available from: https://www.corc.uk.net/media/1219/goalsandgbos-thirdedition.pdf

32. Wright B, Marshall D, Adamson J, Ainsworth H, Ali S, Allgar V, et al. Social Stories ™ to alleviate challenging behavior and social difficulties exhibited by children with autism spectrum disorder in mainstream schools: design of a manualized training toolkit and feasibility study for a cluster randomized controlled trial with nested qualitative and cost-effectiveness components. NIHR Journals Library; 2016.

33. Linton MJ, Mitchell PM, Al-Janabi H, Schlander M, Richardson J, Iezzi A, et al. Comparing the German Translation of the ICECAP-A Capability Wellbeing Measure to the Original English Version: Psychometric Properties across Healthy Samples and Seven Health Condition Groups. Appl Res Qual Life. 2020 Jul 1;15(3):651–73.

34. Janssen MF, Pickard AS, Golicki D, Gudex C, Niewada M, Scalone L, et al. Measurement characteristics of the EQ-5D-5L compared to the EQ-5D-3L across eight patient groups: a multi-country study. Qual Life Res Int J Qual Life Asp Treat Care Rehabil. 2013 Sep;22(7):1717–27.

35. Brosteanu O, Schwarz G, Houben P, Paulus U, Strenge-Hesse A, Zettelmeyer U, et al. Risk-adapted monitoring is not inferior to extensive on-site monitoring: Results of the ADAMON cluster-randomized study. Clin Trials Lond Engl. 2017 Dec;14(6):584–96.

18 list of abbreviations

CATI	Computer-Assisted Telephone Interviews
eCRF	electronic case report form
DSMB	Data Safety and Monitoring Board
EHIS	European Health Interview
FD	Research Database
GCP	Good Clinical Practice
GeDA	Health Germany Current
PiD	Personal Identification Database
PROM	Patient Reported Outcomes
REDCap	Research Electronic Data Capture
SAP	Statistical analysis plan
SOP	Standard Operating Procedure
SP	Social Prescribing
TAU+	Treatment-As- Usual plus brochure with information on local non-clinical support services

19 list of figures

Figure 1: Study design 20
Figure 2: CONSORT flow chart 27

20 list of tables

Table 1: Synopsis 9
Table 2: Project objectives and endpoints 17
Table 3: Intervention according to the TIDieR Framework 21
Table 4: Visit overview 25
Table 5: Objectives and topics of the qualitative process evaluation 36

21 signatures

______________________ ______________________________________

Date, Place Prof. Dr. Wolfram Herrmann, Head of Study

______________________ ______________________________________

Date, Place PD Dr. Stephanie Roll, Biostatistician
